# Apolipoprotein A-I attenuates peritoneal fibrosis associated with peritoneal dialysis by inhibiting oxidative stress and inflammation

**DOI:** 10.3389/fphar.2023.1106339

**Published:** 2023-07-28

**Authors:** Jing Lu, Jie Gao, Jing Sun, Haiping Wang, Huijuan Sun, Qian Huang, Yao Zhang, Shuo Zhong

**Affiliations:** ^1^ Department of Nephrology, Shandong Provincial Hospital Affiliated to Shandong First Medical University, Jinan, China; ^2^ Jinzhou First People’s Hospital, Dalian, China

**Keywords:** peritoneal dialysis, peritoneal fibrosis, apolipoprotein A-I, D-4F, oxidative stress, inflammation peritoneal dialysis, inflammation

## Abstract

Apolipoprotein A-I (apoA-I), 90% of which is present in high-density lipoprotein (HDL), is the main constituent of HDL, has anti-inflammatory and anti-oxidant properties, and has received extensive attention in anti-atherosclerosis. Yet little is known about apoA-I ’s role in peritoneal dialysis. In this study, by analyzing PD patients (*n* = 81), we found that decreased apoA/HDL-C ratio is significantly associated with rapid decline in peritoneal function. Further studies were performed in animal experiments to determine the ascendancy of apolipoprotein A-I mimetic peptide (D-4F) on peritoneum, we found that D-4F administration reduced peritoneal fibrosis and peritoneal endothelial mesenchymal transformation (EMT) induced by high glucose peritoneal dialysate, such as N-cadherin, Fibronectin, Vimentin, and α-smooth muscle actin (α-SMA) expression decreased. In mechanism, D-4F can significantly inhibit Smad2/3 phosphorylation, which is the major pathway leading to fibrosis. Furthermore, D-4F treatment inhibited NADPH oxidase and thiobarbituric acid reactive substances (TBARS) expression, increased the activity of certain enzymes, such as superoxide dismutase (SOD) and glutathione peroxidase (GSH-Px). Finally, treatment with D-4F inhibits the expression of interleukins-6(IL-6), Interleukin-1β(IL-1β), and tumor necrosis factor-α(TNF-α). Taken together, based on the above research evidence, apoA-I and its peptide mimic may regulate the oxidative stress, TGF- β1/Smads signaling pathway and inflammatory response to reduce peritoneal fibrosis due to peritoneal dialysis.

## Introduction

For patients with end-stage renal disease (ESRD), peritoneal dialysis (PD) is an effective renal replacement therapy comparable to hemodialysis in terms of sufficiency, mortality rate and other result parameters. While PD improves the quality of life for ESRD patients, ultrafiltration failure is a serious complication of long-term PD therapy, which restricts patients’ effective duration of dialysis. (Bitar et al, 2022). During peritoneal dialysis, patients’ peritoneum will be exposed to high-concentration glucose dialysate for an extended period of time, which is the primary reason for peritoneal fibrosis and failure of ultrafiltration, leading to conversion to hemodialysis or death, for which there is no effective treatment. Chronic inflammation, oxidative stress, and the fibrotic process itself are implicated in the pathophysiology of peritoneal fibrosis (Hishida et al, 2019; Roumeliotis et al, 2020). oxidative stress and Inflammation often precede the development of peritoneal fibrosis, although there is a bidirectional relationship in which one induces the other (Balzer, 2020). The major causes of oxidative stress in PD are the non-physiologic content of the solutions, high glucose levels, high osmotic pressure, low pH, etc., which promote oxidative reactions in the cells of the peritoneum (Liakopoulos et al, 2017). In addition, when the dialysate is heated for sterilization, it will lead to glucose degradation. The accumulation of its breakdown products and their contact with the peritoneum further promotes the formation of glycosylation end products and pro-oxidant molecules, which in turn results in an increase in fibrosis of the peritoneum (Roumeliotis et al, 2020).Moreover, these factors can lead to both local inflammation of the peritoneum and a microinflammatory state in PD patients, which play a significant role in fibrosis of the peritoneum. A hallmark of inflammation is the production of a greater number of inflammatory mediators, such as tumor necrosis factor-α (TNF-α), c-reactive protein and diver interleukins (ILs) (Zhou et al, 2016). Therefore, the search for biological molecules that are antioxidant and anti-inflammation is likely to be one of the effective ways to prevent and treat peritoneal fibrosis.

In the general population, HDL exerts protective effects by affecting reverse cholesterol transport, besides the anti-inflammatory and antioxidant effects (Rosenson et al, 2012; Kontush, 2020; Xepapadaki et al, 2020; Jia et al, 2021). It has been shown that the reduction in HDL levels in patients with ESRD is due to decreased synthesis, increased decomposition, and exception clearing of HDL (Zhong et al, 2019; Noels et al, 2021). Subsequent analyses demonstrated several changes in the composition of HDL lipoprotein granules in patients with CKD, including apoA-I, apoA-II and apoM levels decreasing and serum amyloid a (SAA), apoC-II, apoC-III and apoA-IV levels increasing, the changes in these components may be associated with HDL- C dysfunction in PD patients (Zhong et al, 2019). However, because apoA-I or HDL-C levels may not reflect key characteristics of lipoproteins, it is unclear whether low apoA-I or HDL levels or changes in their associated fraction ratios affect peritoneal ultrafiltration function in PD patients. Therefore, one of the goals of this project was to examine the relationship between changes in plasma HDL-C, apoA-I and other lipid components and changes in peritoneal ultrafiltration function in patients with PD.

ApoA-I mimics peptide 4F is a polypeptide with 18 amino acids, in which 4 amino acids are replaced with phenylalanine, so it is referred to as 4F. The properties of 4F are the same as those of apolipoprotein A-I, such as lipid-binding properties. In addition, ApoA-I mimetic peptide 4F has the advantage of small molecular weight for clinical application and promotion as well as easy synthesis, and it has been synthesized from all-L amino acids (L-4F) and all-D amino acids (D-4F) (Van Lenten et al, 2009). At present, D-4F has been shown in animal experiments to have an anti-inflammatory, antioxidant, reverse lipid transport, and angiogenesis regulation similar to that of apoA-I (Li et al, 2004; Qin et al, 2012; Yao et al, 2015).However, it remains unknown whether Apo-I mimetic peptide 4F can exert anti-inflammatory and antioxidant effects under chronic inflammatory conditions induced by PD fluid.

Therefore, the purpose of this present study was to investigates the levels of lipid constituents in plasma of PD patients and their ratio to HDL and analyze their correlation with the function of the peritoneum. In addition, the effect and mechanism of D-4F on peritoneal fibrosis induced by high glucose PD fluid in rats was analyzed.

## Materials and methods

### Research design and population

This was a retrospective observational cohort study that was conducted on 81 patients who undergoing continuous ambulatory peritoneal dialysis (CAPD) in the provincial hospital affiliated to Shandong First Medical University from October 2011 to August 2021. Study inclusion criteria were as follows: over 18 years of age and maintained PD therapy for at least 1 month. Patients who met the exclusion criteria were those with previous peritoneal infection, previous history of kidney transplantation, ≥3 months of previous hemodialysis, individuals with coronary atherosclerotic heart disease, known history of familial hyperlipidemia, chronic liver disease and severe liver dysfunction, concurrent pregnancy or malignancy, blood transfusion therapy within 3 months prior to study, and incomplete lipid data. All patients were classified into low transport group (D/P 0.34–0.64) and high transport group (D/P 0.65–1.03) based on the results of each peritoneal equilibration test (PET), 153 times in total. Moreover, 39 patients with peritoneal dialysis >24 months performed PET in both 3 and 24 months were selected and grouped according to the above criteria. The patient’s past medical records were collected, including LDL-C, HDL-C level, apolipoprotein A level, apolipoprotein B level, PET D/*p* values, and other clinical information. This research protocol was approved by the Affiliated Provincial Hospital Clinical Research Ethics Committee of Shandong First Medical University.

### Animal models and animal groups

The experimental animal program was carried out in conformity to the principles of the animal ethics committee of Shandong First Medical University Affiliated Provincial Hospital. 18 male rats from Shandong Provincial Hospital Animal Center, aged 6–8 weeks, were randomly divided into 3 groups: 1) control group; 2) PDF group; and 3) PDF + D-4F group. 4.25% glucose PDF (Baxter Healthcare Ltd., Deerfield, IL, United States) injection was used as the peritoneal dialysis fluid in rats, and 6 weeks of daily intraperitoneal injection in PDF rats were used to successfully established peritoneal dialysis animal model. Control group received the same quantity of saline (NS). The D-4F + PDF group received oral D-4F at 20 mg/kg/D 2 weeks after the start of the trial, the NS was used as a control for the rats in the PDF group. After 4 weeks of oral administration of D-4F, serum was taken, all died, and the parietal peritoneum was taken.

### Reagent

4F was synthesized using all D-amino acids in sequence (Ac-DWFKAFYDKVAEKFKEAF-NH2) and was purchased from Nanjing Peptide Biotechnology Co., Ltd. Antibodies against Fibronectin (no.15613-1-AP), Collagen-I (no.14695-1-AP), N-cadherin (no.22018-1-AP), α-SMA (no.14395-1-AP), TGF-β1 (no.21898-1-AP) were purchased from Proteintech. E-cadherin (no.3195T), Antibodies against phospho-Smad2/3 (no.13820T), phospho-NF-κB p65 (no. 3033T) were purchased from Cell Signaling Technology. Antibodies against Vimentin (no.ab925470, NOX2 (no.ab129068), NOX4 (no.ab133303), phospho-Erk1/2 (no.ab201015) were purchased from Abcam. Antibodies against GAPDH (no.AC002) was purchased from Abclonal. Secondary antibodies conjugated to goat anti-rabbit and goat anti-mouse horseradish peroxidase (ZB-2301 and ZB-2305, respectively) were purchased from Zhongshan Jinqiao. Thiobarbital acid reactant colorimetric (TBARS) test kit (E-BC-K318-M), Superoxide dismutase (SOD) typing test kit (e-bc-k022-m) and glutathione peroxidase (GSH-PX) colorimetric Kit (e-bc-k096-m) were purchased from elabscience.

### Western blot analyses

The appropriate amount of peritoneal tissue should be taken for total protein extraction, and the concentration of total protein extracted should be determined by the BCA protein method. The same amount of protein (20 μg) is removed and added to 8%–12% SDS-PAGE gel for separation of proteins and transferred to polyvinyl fluoride membrane (Millipore). Dilutions of the following antibodies were made and cocultured with the membrane overnight at 4°C: E-cadherin (1:1,000), Fibronectin (1:2000),N-cadherin (1:2,000), α-SMA (1:1,000), vimentin (1:1,000), phospho-Smad2 (1:1,000), phospho-Smad3 (1:2,000), TGF-β1 (1:1,000), phospho-NF-κB p65 (1:1,000), NOX2 (1:5000), NOX4 (1:1000), phospho-Erk1/2 (1:1000) and GAPDH (1:5000). Then incubate the membrane with horseradish peroxidase bound secondary antibody (1:5000) for 1 h at room temperature. The ECL system and Bio Rad electrophoretic image analyzer were used to observe the immune reaction zone after the developer was added.

### Elisa

Obtained from rat serum by orbital blood collection and centrifuging. Follow the instructions in the kit, the activity of CuZn-SOD and GSH-Px in serum were detected in biochemical kits. For MDA determination, peritoneal tissue homogenate was first performed, and then the homogenate was centrifuged at 14.000rpm for 10 min at 4°C. The resulting supernatant was used for MDA determination.

### RNA extraction and real-time PCR

Total RNA extractions and concentration determinations. cDNA synthesis according to the specification of SYBR Green. The sequences of the primers used for real-time PCR are: IL-6, forward 5′-AAG​CCA​GAG​CTG​TGC​AGA​TGA​GTA-3′ and reverse 5′-TGT​CCT​GCA​GCC​ACT​GGT​TC-3′; TNF-α, forward 5′-CTG​CCT​GCT​GCA​CTT​TGG​AG-3′ and reverse 5′-ACA​TGG​GCT​ACA​GGC​TTG​TCA​CT-3′; IL-1β, forward 5′-CCA​GGG​ACA​GGA​TAT​GGA​GCA-3′ and reverse 5′-TTC​AAC​ACG​CAG​GAC​AGG​TAC​AG-3′; and actin, forward 5′-ATT​GCC​GAC​CGA​ATG​CAG​A-3′ and reverse 5′-ATG​GAG​CCA​CCG​ATC​CAG​AC-3′.

### Morphological and immunohistochemical analysis of peritoneum

The peritoneum samples fixed in 4% paraformaldehyde underwent a series of standard procedures including successive dehydration in a graded alcohol series (75%, 85%, 95%, and 100%, v/v), transparency in dimethylbenzene and paraffin embedding, and then were sectioned into 5 µm thick slices. Dewaxing and hydration were performed on the slices, followed by dropwise addition of hematoxylin staining solution, differentiation solution, and eosin staining solution on the slices for Hematoxylin eosin (H & amp; E) staining. Masson staining was used to study the fibrosis of peritoneal specimens. We utilized ImageJ (National Institute of Health, Bethesda, MD), a computerized image analysis software, to evaluate PF by measuring the percentage of peritoneal membrane occupied by collagen fibers. In addition, peritoneum thickness was assessed by measuring the distance from the surface mesothelium to the upper limit of the muscular tissue with the aid of ImageJ software. For each sample, select 5 regions. Peritoneal tissue sections were subjected to immunohistochemical staining using a two-step detection kit and a developed DAB reagent. The slices were incubated in a wet box in a primary antibody at 4°C overnight. The primary antibodies included anti-Fibronectin (1:500), anti-TGF-β1 (1:400), and anti-α-SMA (1:500), Anti-collagen I (1:500). The next day, Secondary antibodies (1:200) were incubated in slices for 2 h. Observations by fluorescence microscopy.

### Statistical analysis

All data are expressed as means ± SDs. Non-sample paired *t*-test and paired sample *t*-test were used to determine statistical significance. *p* < 0.05 was regarded as statistically significant. Graphpad prism 8 and SPSS Statistics 22 were used for all calculations.

## Results

### Clinical information and laboratory values of study population

This study recorded and analyzed the relevant information of 81 CAPD patients. Table 1 lists the demographic and clinical data characteristics of the selected patients. A comparison of the level of blood lipid laboratory values between the high-transport and low-transport groups is shown in Table 2. No significant differences were observed in serum HDL-C, LDL-C, apoB, apoA, apoB/HDL-C, apoA/apoB levels between the two groups (*p* > 0.05), however the apoA/HDL-C level and D/D0 glucose in high transport group were obviously lower than that in low transport group (*p* < 0.05).

**TABLE 1:** Baseline characteristics of patients.

General features	Data
Age (years)	50.52 ± 14.56
Dialysis time (month)	17.8 ± 11.72
Gender (case%)	
male	46 (57%)
female	35 (43%)
Primary disease (cases%)	
Chronic glomerulonephritis	32 (40%)
Diabetes nephropathy	6 (7%)
Hypertensive nephropathy	31 (38%)
other	12 (15%)
BMI (Kg/m^2^)	22 ± 2.93
TG (mmol/l)	1.82 ± 0.99
TC (mmol/l)	4.79 ± 1.52
HDL-C (mmol/l)	1.22 ± 0.41
LDL-C (mmol/l)	2.82 ± 0.86
ApoA (g/l)	1.05 ± 0.23
ApoB (g/l)	1.00 ± 0.33
ALB (g/l)	35.09 ± 5.27
Cr (umol/l)	977.45 ± 320.93
eGFR (mL/min)	5.46 ± 2.73
Urine volume (ml)	1354.42 ± 504.06

Data are presented as means ± standard deviation (SD) or n (%). Abbreviations: BMI, body mass index; TG, triglyceride; TC, total cholesterol; ApoA, apolipoprotein A; ApoB, apolipoprotein B; HDL-C, high-density lipoprotein cholesterol; LDL-C, low-density lipoprotein cholesterol; ALB, albumin; Cr, blood creatinine; eGFR, glomerular filtration rate.

**TABLE 2:** Lipid composition between high transport group and low transport group.

Biochemical index	High transport group	Low transport group	*p*-value
HDL-C (mmol/l)	1.30 ± 0.42	1.41 ± 0.84	0.32
LDL-C (mmol/l)	3.63 ± 0.78	2.84 ± 0.72	0.34
ApoA (g/l)	1.07 ± 0.21	1.13 ± 0.34	0.22
ApoB (g/l)	0.95 ± 0.26	0.98 ± 0.29	0.51
ApoA/HDL-C	0.76 ± 0.15	0.89 ± 0.28	0.00
ApoB/HDL-C	0.71 ± 0.23	0.77 ± 0.31	0.17
ApoA/apoB	1.16 ± 0.36	1.24 ± 0.37	0.23
D/D0	0.37 ± 0.11	0.47 ± 0.1	0.00

Means ± standard deviations (SDs) are expressed for normally distributed variables. Abbreviations: ApoA, apolipoprotein A; ApoB, apolipoprotein B; HDL-C, high-density lipoprotein cholesterol; LDL-C, low-density lipoprotein cholesterol; D/D0: 4 to 0 h dialysate glucose.

### ApoA/HDL-C ratios are related to the filtration function of peritoneum

**FIGURE 1:**
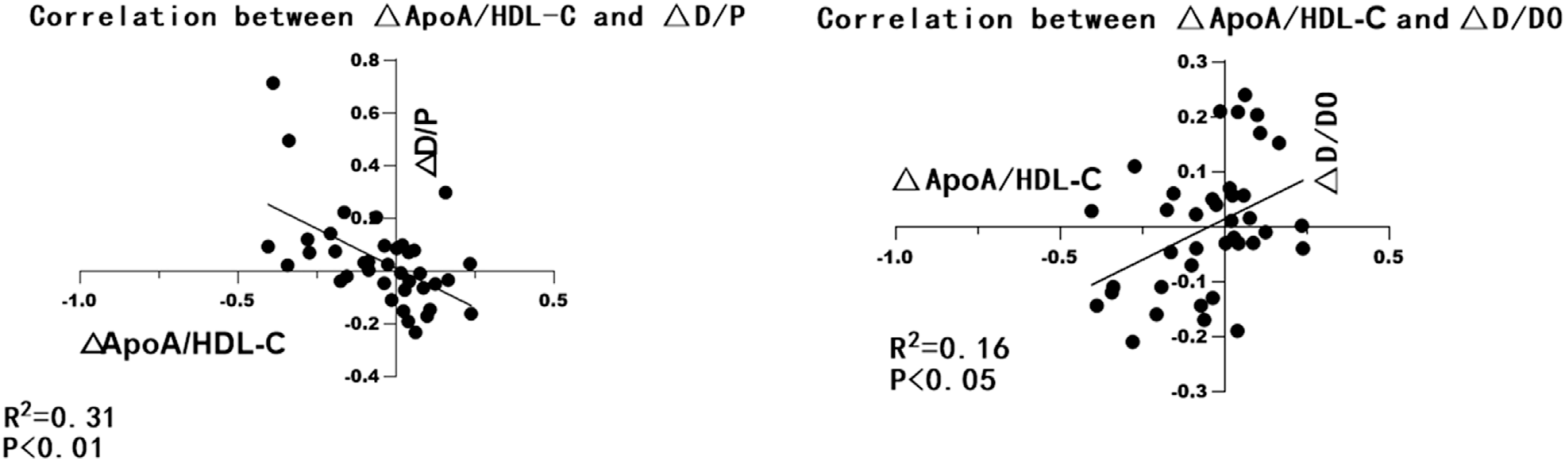
GraphPad Prism 8 was used to make the correlation analysis chart of △ApoA/HDL-C and △D/P, △D/D0.

A total of 39 patients wih peritoneal dialysis >24 months performed PET in both 3 and 24 months were selected and grouped according to D/*p* values. The correlation of △D/P and △apoA/HDL-C between 3 and 12 months was analyzed. As showed in Table3 and Figure 1, the △apoA/HDL-C was negatively correlated with the △D/*p* values (r = −0.58, *p* < 0.01), and positively correlated with △D/D0 (r = 0.33, *p* < 0.05).

**TABLE 3:** ApoA/HDL-C ratios are related to the filtration function of the peritoneum.

Biochemical index	Difference	△D/P		△D/D0	
		r	P	r	P
△HDL-C	0.06 ± 0.45	0.08	0.64	−0.28	0.09
△LDL-C	−0.01 ± 0.75	0.05	0.77	−0.16	0.33
△APOA	−0.07 ± 0.27	−0.24	0.14	−0.05	0.75
△APOB	−0.05 ± 0.32	−0.03	0.87	−0.11	0.53
△APOA/HDL	−0.08 ± 0.21	−0.58	0.00	0.33	0.04
△APOB/HDL	−0.04 ± 0.25	−0.2	0.22	0.12	0.47

Abbreviations: ApoA, apolipoprotein A; ApoB, apolipoprotein B; HDL-C, high-density lipoprotein cholesterol; LDL-C, low-density lipoprotein cholesterol,△, the 24-month dialysis minus the 3-month dialysis value.

### D-4F reduced the peritoneal fibrosis by regulating the EMT process in PD rats

**FIGURE 2:**
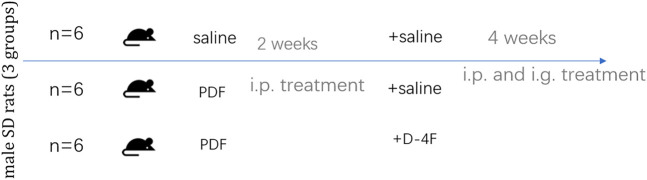
Schematic of the study design. Male SD rats were subjected to 6 weeks of daily treatment with either saline or high-glucose (4.25%)-containing PD fluid (PDF) ± D-4F (20 mg/kg body weight).

**FIGURE 3:**
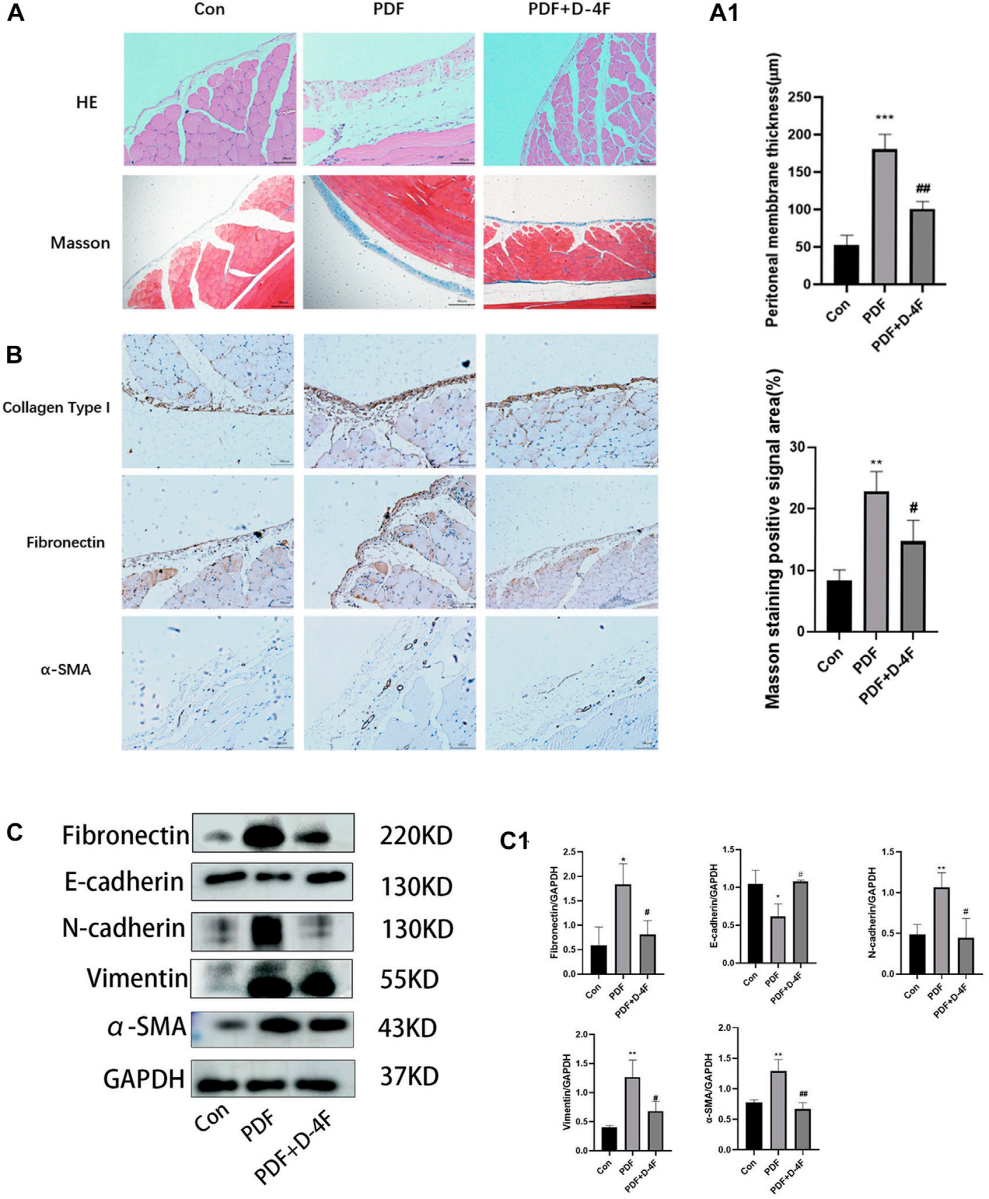
Peritoneal dialysis fluid-induced fibrosis in mice is attenuated by D-4F. **(A)** Masson trichrome staining and H&E staining of peritoneum with or without D-4F treatment is illustrated in photomicrographs (magnification: ×200; scale bars = 100 μm). Mean peritoneal membrane thickness and peritoneal fibrosis score in rat are shown in **(A1)**. **(B)** Collagen I, Fibronectin, α-SMA expression levels were observed using immunohistochemistry (magnification: ×200; scale bars = 100 μm). **(C)** the peritoneum was taken for immunoblot analysis of E-cadherin, N-cadherin, Vimentin, Fibronectin, α-SMA and GAPDH. Representative immunoblots from three experiments are shown. **(C1)** Using GAPDH as loading control for quantitative analysis. The data represent an analysis of at least three independent experiments. *p < 0.05, **p < 0.01, ***p < 0.001 compared with the Con group; #*p* < 0.05 and ##*p* < 0.01 compared with the PDF group.

With the aim of researching the role and mechanism of apoA in fibrosis of the peritoneum, we investigate the role of D-4F, one of the apoA-I mimic peptides, in a rat model of peritoneal dialysate-induced peritoneal fibrosis. Rats were given saline or PDF for 6 weeks, with or without D-4F (20 mg/kg/d) orally for 4 weeks (Figure 2). As expected, pronounced sub-mesothelial thickening developed after a 6 weeks exposure to PDF, while D-4F treatment showed a greater rescuing effect in terms of peritoneal thickness (Figure 3A, A1). EMT from peritoneal mesothelial cells has been shown to play a significant role in PF. Immunohistochemistry or Western blot analysis determined the expression of the epithelial adhesion protein marker E-cadherin, the mesenchymal marker α-smooth muscle actin (α-SMA), N-cadherin and vimentin, and the fibrosis markers Fibronectin and collagen type I (Figures 3B,C). The results showed that high-glucose treatment increased the expression of N-cadherin, α-SMA, Fibronectin, vimentin and Collagen I in peritoneal tissue, but significantly decreased the expression of E-cadherin. The effect was blunted by oral administration of D-4F (Figures 3A–C).

### D-4F suppressed the TGF-β1/smad signaling pathway in peritoneal fibrosis rats

**FIGURE 4:**
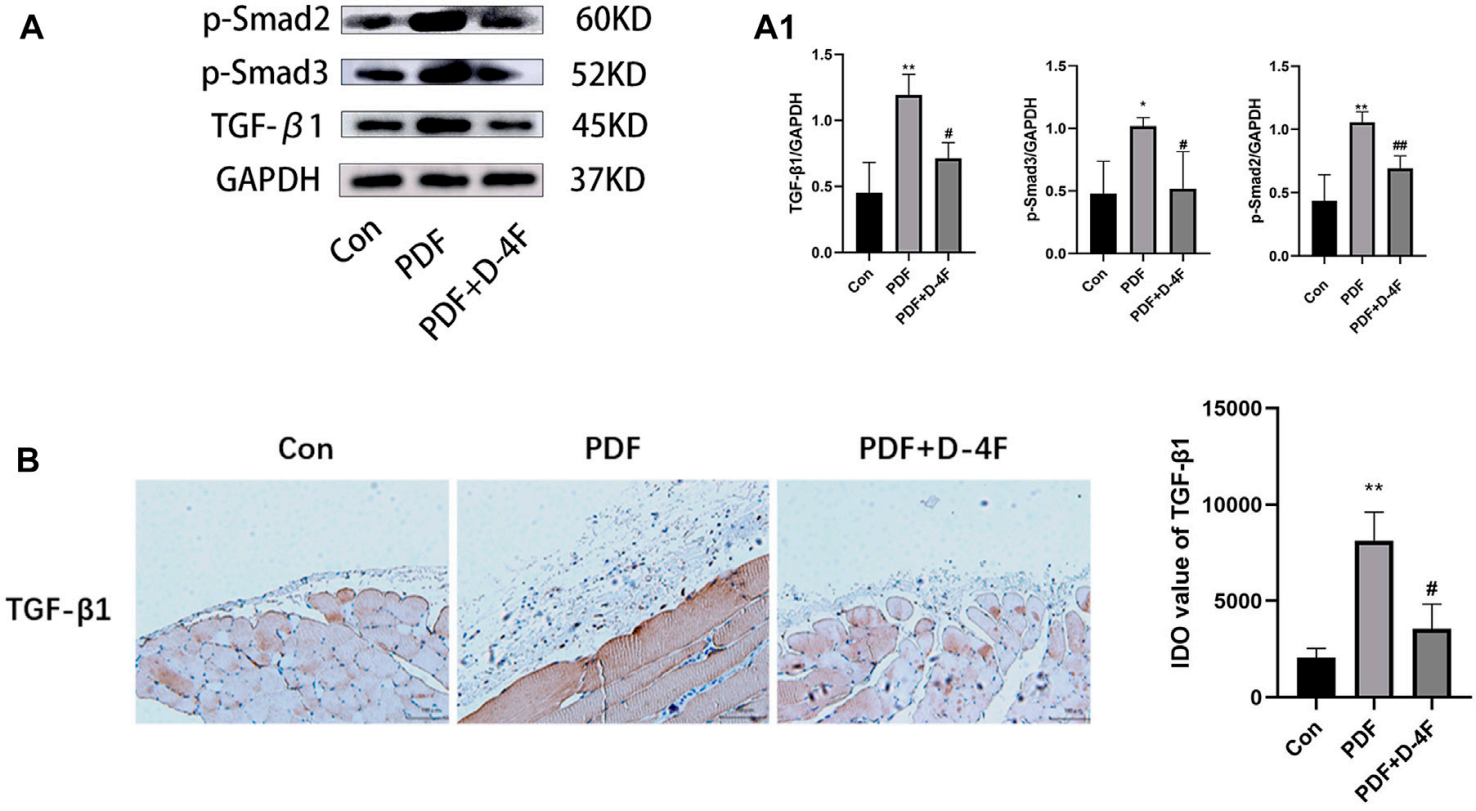
D-4F can inhibit TGF-β/Smad pathway. **(A)** The peritoneum was taken for immunoblot analysis of TGF-β1, p-smad2, p-smad3 and GAPDH. Representative immunoblots from three experiments are shown. **(A1)** Using GAPDH as loading control for quantitative analysis. The data represent an analysis of at least three independent experiments. *p < 0.05, **p < 0.01 compared with the Con group; #*p* < 0.05 and ##*p* < 0.01 compared with the PDF group. **(B)** TGF-β1 expression levels were observed using immunohistochemistry (magnification: ×200; scale bars = 100 μm), and semiquantitative analysis of data shown in **(B)** *p < 0.05, **p < 0.01 compared with the Con group; #*p* < 0.05 compared with the PDF group.

To further investigate the mechanism behind the anti-fibrotic effect of D-4F, we investigated whether D-4F was acting on the TGF-β1-induced activation of the Smad pathway. Western blot analysis revealed that the TGF-β1/Smad signal transduction activity of the PDF group was higher than that of the control group, which showed increased TGF-β1 expression and increased phosphorylation of Smad2/3. However, D-4F treatment significantly suppressed TGF-β1 and p-Smad2/3 levels (Figure 4A, A1). TGF-β1 has the same trend as Western blotting by immunohistochemical analysis (Figure 4B).

### D-4F attenuated the oxidative stress in PD rats

**FIGURE 5:**
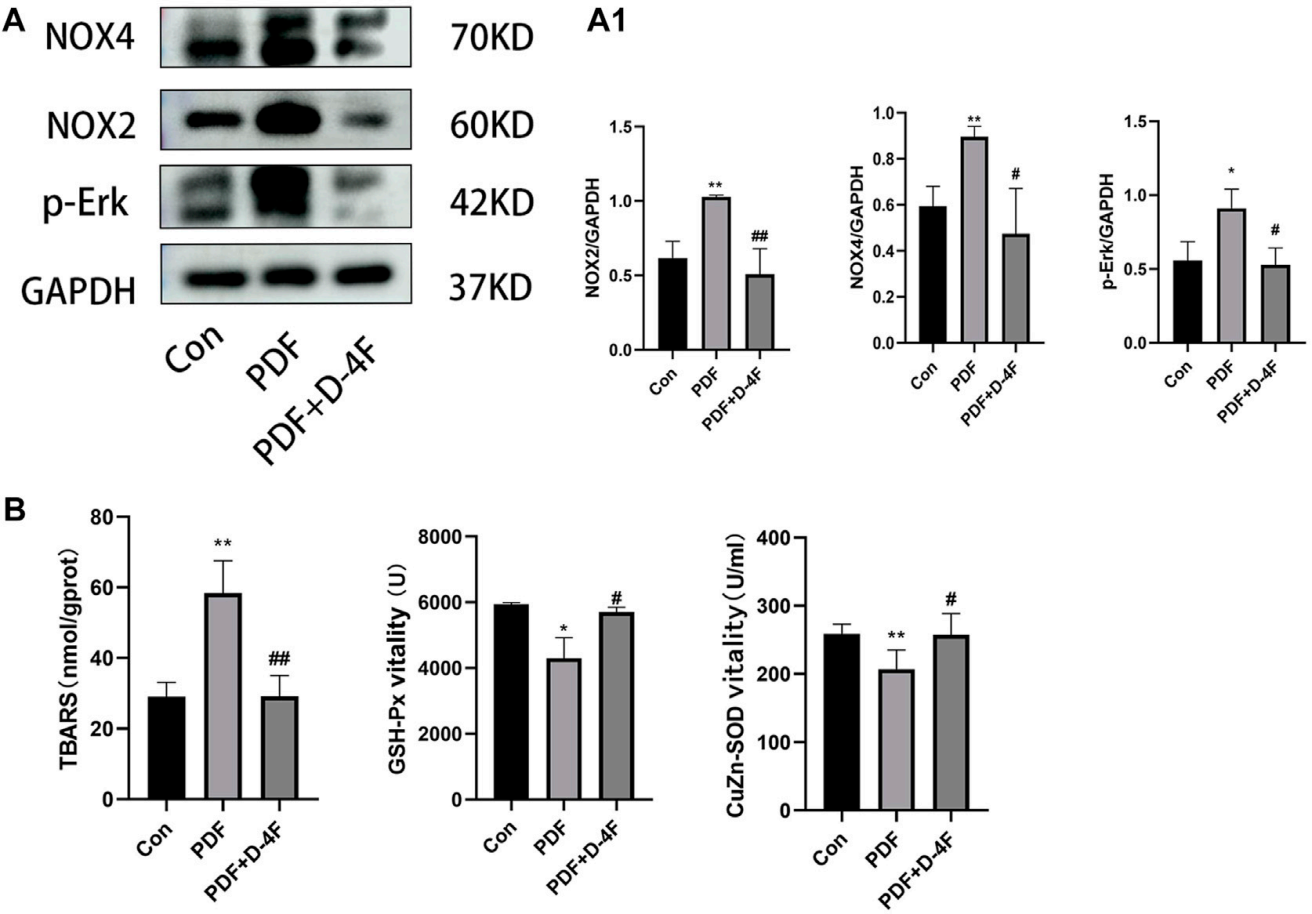
D-4F may inhibit oxidative stress during peritoneal dialysis. **(A)** Western blot was used to detect the expressions of NOX2, NOX4 and p-Erk in the control group, peritoneal dialysis model group and D-4F treatment group. **(A1)** Using GAPDH as a loading control, the results were quantitatively analyzed, *p < 0.05, **p < 0.01, compared with the Con group; #*p* < 0.05 and ##*p* < 0.01 compared with the PDF group. **(B)** The concentration of TBARS and the activities of serum CuZn-SOD, GSH-PX in the control group, peritoneal dialysis model group and D-4F treatment group were detected by colorimetry.

Moreover, oxidative stress is strongly associated with chronic inflammation and secondary fibrosis of PD. In order to elucidate the effect of D-4F on oxidative stress, we detected the expression of NOX2, NOX4 and p-Erk in PD rats by Western blotting. As showed in shown in Figure 5A, A1, the expression of NOX2 NOX4 and p-Erk was increased by exposure to peritoneum dialysate, which was significantly blocked by D-4F. Furthermore, we found that D-4F treatment significantly reduced the concentration of TBARS in Peritoneal tissue and enhanced the activity of CuZn-SOD and GSH-Px in plasma, indicated that D-4F reversed a decrease in anti-oxidant activity (Figure 5B).

### D-4F reduced inflammatory cytokines secretion in PD rats

**FIGURE 6:**
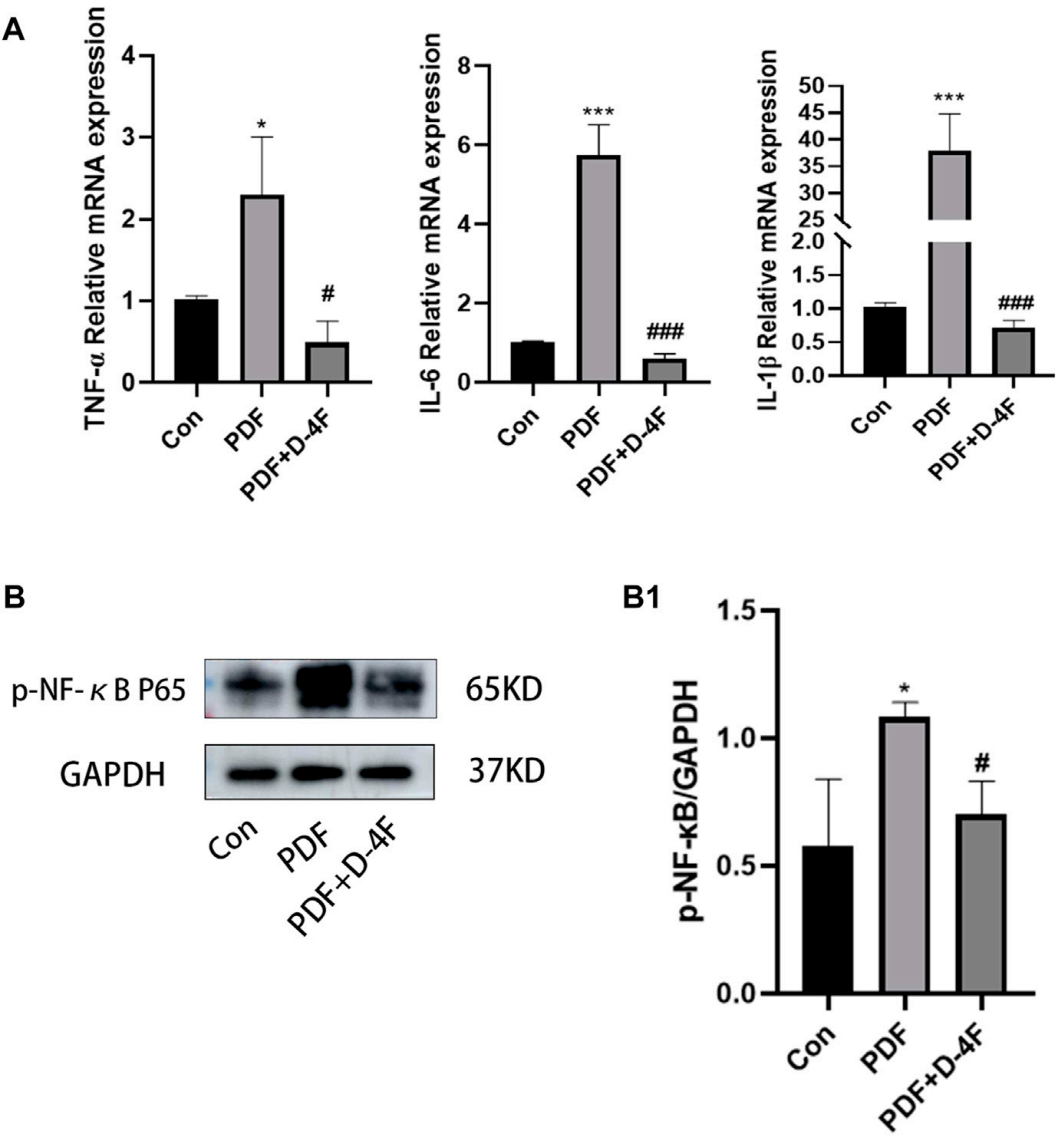
D-4F can inhibit inflammatory reaction during peritoneal dialysis. **(A)** The expression levels of IL-6, IL-1β and TNF-α in the control group, peritoneal dialysis model group and D-4F treatment group were detected by Real-time PCR. Using β-ACTIN as a loading control, the results were quantitatively analyzed. **(B)** Western blot was used to detect the expression of p-NF-κB p65 in the control group, peritoneal dialysis model group and D-4F treatment group. **(B1)** Results were quantitatively analyzed using GAPDH as a loading control. *p < 0.05, **p < 0.01, ***p < 0.001 compared with the Con group; #*p* < 0.05, ##*p* < 0.01and ###*p* < 0.001 compared with the PDF group.

We further explored the effects of D-4F on inflammation. From these results, it is known that the mRNA levels of IL-1β, IL-6 and TNF-α in the PDF group were significantly increased, whereas D-4F treatment obviously inhibited this pro-inflammatory component (Figure 6A). The NF-κB is among the classic transcription factors that can direct the expression of multiple cytokine/chemo kinesins. As shown in Figure 6B, high levels of phosphorylation of NF-κB were detected in PD rats by Western blotting, and the expression was obviously downregulated by D-4F. Moreover, in H;E staining (Figure 3A), we found that the number of peritoneal inflammatory cells in the PDF group was significantly higher than that in the Con group and the D-4F + PDF group, which is consistent with our conclusion.

## Discussion

Peritoneal dialysis (PD) has been used extensively in patients with end-stage renal disease (ESRD). The other side of the shield, chronic exposure of the peritoneum to bioincompatible PD solutions during long-term dialysis leads to peritoneal fibrosis (PF), thus contributing to the failure of ultrafiltration, which is, at least in part, related to lipid abnormalities. In this study, we describe that apoA-I is a protective factor of peritoneal ultrafiltration function in patients with PD. D-4F treatment ameliorated the high-glucose-induced PF by suppressing EMT via TGF-β/Smad signaling in rats. In addition, D-4F significantly reduced the levels of inflammatory cytokines and oxidative stress, indicating the possible mechanism of apoA-I in reduced PF.

Apolipoprotein A-I (ApoA-I) takes up the majority of the high density lipoprotein (HDL) components. Despite the fact that each HDL particle contains five apoA-I molecules, the systemic apoA-I levels were used as an indicator for HDL cholesterol concentrations (Catapano et al, 2016). It has been suggested in previous studies that PD patients will have changes in lipid mass spectrometry during dialysis with an increase in serum total cholesterol, very low-density lipoprotein cholesterol (VLDL), and low-density lipoproteins (LDL) levels, whereas high-density lipid cholesterol and apolipoprotein A (apoA) levels decrease (Steele et al, 1989; Holzer et al, 2015). One of the largest meta-analyses of prospective studies in general populations demonstrates an inverse association between apoA-I and incident coronary artery disease (Holmes et al, 2018). Furthermore, a higher apoB/apoA-I ratio is highly correlated with an increase in all-cause mortality and cardiovascular events in PD patients (Zhan et al, 2018). Recent research demonstrates that HDL: apoA-I ratio has an independent relationship with all-cause mortality in patients with PD (Zeng et al, 2021). However, the relationship between apoA-I and peritoneal ultrafiltration function remains unclear.

As noted above, we found that the serum apoA/HDL-C levels of PD patients with elevated peritoneal transport function based on PET findings were reduced. △ApoA/HDL-C value between 3 and 24 months after the first PD treatment was also found to be negatively correlated with △D/*p*-value, indicating that apoA exerts a protective effect on the peritoneal ultrafiltration function of patients with PD. This study further investigated the effect of apoA-I on peritoneal fibrosis in rat PF models induced by high-glucose peritoneal dialysis solution. The results showed effectively anti-fibrotic effects of apoA-I mimicking peptides (D-4F) on peritoneal fibrosis, which participated in regulating EMT, inflammatory and oxidative stress.

Recent research has demonstrated that there are a variety of causes of peritoneal fibrosis including inflammation, oxidative stress, and epithelial-to-mesenchymal transition (EMT), and that these processes interact and promote each other (Balzer, 2020). The basic properties of EMT are to destroy cell polarity and adhesion and to obtain mesenchymal characteristics including migration and invasion (Thiery and Sleeman, 2006; Dongre and Weinberg, 2019). Previous studies have indicated that high-glucose treatment induces EMT in human peritoneal mesothelial cells, which is associated with a decline in peritoneal function as a result of peritoneal fibrosis (Yang et al, 2018; Su et al, 2020). During the study of patients with idiopathic lung fibrosis, apolipoprotein A-I (apoA-I) in the BAL solution was found to be significantly decreased (Kim et al, 2010). D-4F was used for further research, which demonstrated that D-4F inhibited TGF-β1 induced EMTs in alveolar cells and IL-4 induced alternative activation of macrophage in human acute monocytic leukemia cells (You et al, 2016; Song et al, 2019). However, while studies have shown that apoA-I has specific anti-fibrotic potential, only a handful of studies have provided information on the role of D-4F peritoneal fibrosis. In this study, we reveal for the first time that apoA-I participates in peritoneal fibrosis through the regulation of EMT. EMT is a process induced by a variety of growth factors, in particular transformative growth factor-β (TGF-β), which was considered to be a key mediator of mesothelial EMT both *in vitro* (Yang et al, 2003) and *in vivo* (Margetts et al, 2005). Two TGFβ1-induced signal transduction pathways have been identified, the Smad-dependent pathway and the Smad-independent pathway, and most profibrotic effects are accomplished via the Smad dependent canonical pathway (Wang et al, 2005; Bottinger, 2007; Lan, 2011; Zhou et al, 2016). TGF-β1 initiates the EMT process with multiple steps. First, it interacts with TGF-β Type II receptor binding activates TGF-β Type I receptors then phosphorylate the downstream Smad2/3 proteins following their phosphorylation and Smad4 binding transmits signals to the nucleus, where they initiate the EMT process in conjunction with transcription factors such as snail and twist (Shi and Massague, 2003; Aroeira et al, 2007; Hao et al, 2019). Inhibition of SGLT-2 using empagliflozin has been reported to ameliorate peritoneal fibrosis via suppressing TGF-β/Smad signaling (Shentu et al, 2021). We indeed found in this study that peritoneal dialysate induced EMT consistent with increased TGF-β1 and phosphorylated-Smad2/3 expression, which can be suppressed by D-4F treatment. The results of this study have important implications for understanding the cellular mechanisms of apoA-I in peritoneal fibrosis.

Under repeated stimuli of long-term non-physiological peritoneal dialysate components, peritonitis, uremia toxin, or other micro-inflammation, oxidative stress (OS) throughout the whole process of peritoneal fibrosis.

Previous studies have demonstrated that, in terms of a healthy control population, the serum levels of markers of OS are markedly lower than those of patients with PD (Kocak et al, 2008). Moreover, compared to patients undergoing hemodialysis, significantly elevated plasma levels of myeloperoxidase (MPO) and the state of lipid peroxidation were reported (Taylor et al, 1992; Al-Hweish et al, 2010). D-4F has been reported to reduce ROS generation by inhibiting PKC, P47 activation, and eNOS uncoupling via AMPK (Guo et al, 2020). To further investigate the antioxidant effect of D-4F in preventing peritoneal fibrosis, we examined the activity of CuZn-SOD and Px-GSH in serum and the protein expression of NOX2 and NOX4 in peritoneal tissue of PF rats. Similar results have been obtained in our current study, suggesting that apoA-I has a potential antioxidant stress effect on PF induced by high-glucose peritoneal dialysis solution.

Peroxidation-antioxidation imbalance not only increase the level of oxidative stress, but also activate monocyte chemoattractant protein-1 [MCP-1], TNF-a, IL-1β and other proinflammatory factors, as well as infiltration of various inflammatory cells (Wang et al, 2016). Monocyte and macrophage infiltration and excessive proinflammatory cytokine production have been shown to accelerate peritoneal fibrosis (Bellon et al, 2011; Ko et al, 2019; Terri et al, 2021). The anti-inflammatory property of HDL could directly lead to cell adhesion molecules and tumor necrosis factor-alpha (TNF-α) downregulation of expression level (Wang et al, 2018). There is no doubt that NF-κB pathway is an important transcription factor regulating gene expression or production of various inflammatory cytokines and chemokines under various pathologic conditions (Chen et al, 2015). In this study, the expression of TNF-a, IL-6, IL-1β, and NF-κB was significantly elevated in the peritoneum of PF rats, which was inhibited by D-4F treatment.

In summary, we found that apoA-I plays a protective effect on peritoneal ultrafiltration function of PD patients. This beneficial effect may be due to suppression of multiple profibrotic signaling pathways, oxidative stress and inflammatory responses. Therefore, application of apoA-I could be an effective method for preserving the ultrafiltration ability of the peritoneal membrane. Additional studies are needed to assess the practicability and validity of apoA-I substitutes in patients with fibrosis of the peritoneum.

## Data Availability

The original contributions presented in the study are included in the article/Supplementary Material, further inquiries can be directed to the corresponding authors.
