# Corrigendum: Apolipoprotein A-I attenuates peritoneal fibrosis associated with peritoneal dialysis by inhibiting oxidative stress and inflammation

**DOI:** 10.3389/fphar.2024.1532774

**Published:** 2024-12-16

**Authors:** Jing Lu, Jie Gao, Jing Sun, Haiping Wang, Huijuan Sun, Qian Huang, Yao Zhang, Shuo Zhong

**Affiliations:** ^1^ Department of Nephrology, Shandong Provincial Hospital Affiliated to Shandong First Medical University, Jinan, China; ^2^ Jinzhou First People’s Hospital, Dalian, China

**Keywords:** peritoneal dialysis, peritoneal fibrosis, apolipoprotein A-I, D-4F, oxidative stress, inflammation peritoneal dialysis, inflammation

In the published article, there was an error in the **Funding** statement. The funding details for “Jinan Science and Technology Development Plan - Clinical Medicine Technology Innovation Plan (No. 202225049)” were erroneously omitted. The correct **Funding** statement appears below.

