# Supplementary figures and images for: Apolipoprotein A-I attenuates peritoneal fibrosis associated with peritoneal dialysis by inhibiting oxidative stress and inflammation
Source: Front Pharmacol. 2023 Jul 28;14:1106339. doi: 10.3389/fphar.2023.1106339 (PMC10422021; doi:10.3389/fphar.2023.1106339)

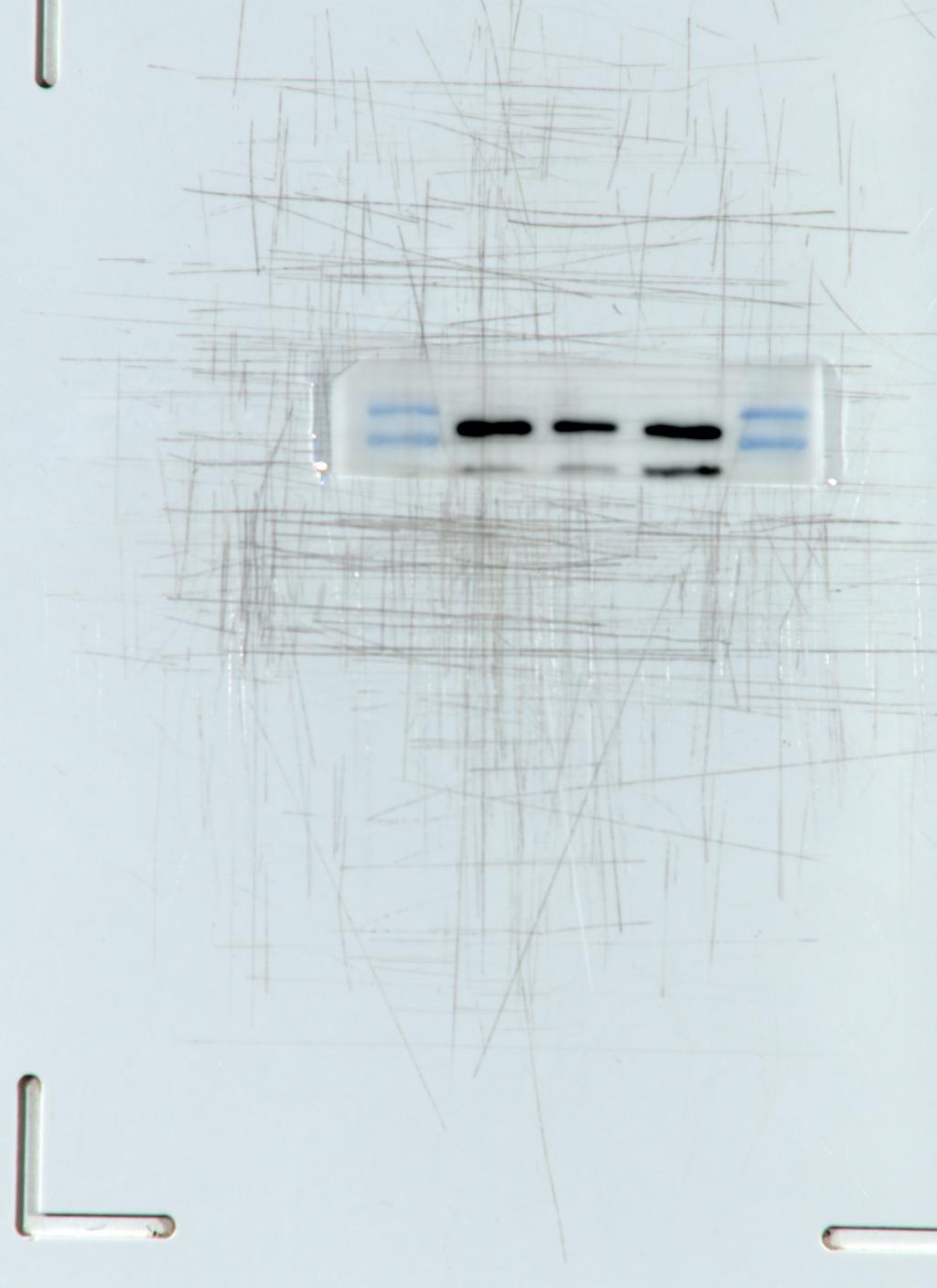

Supplement: Supplementary file 1 [file DataSheet1.ZIP › Raw Data 2/WB/figure 3C/E-ca 10.jpg]

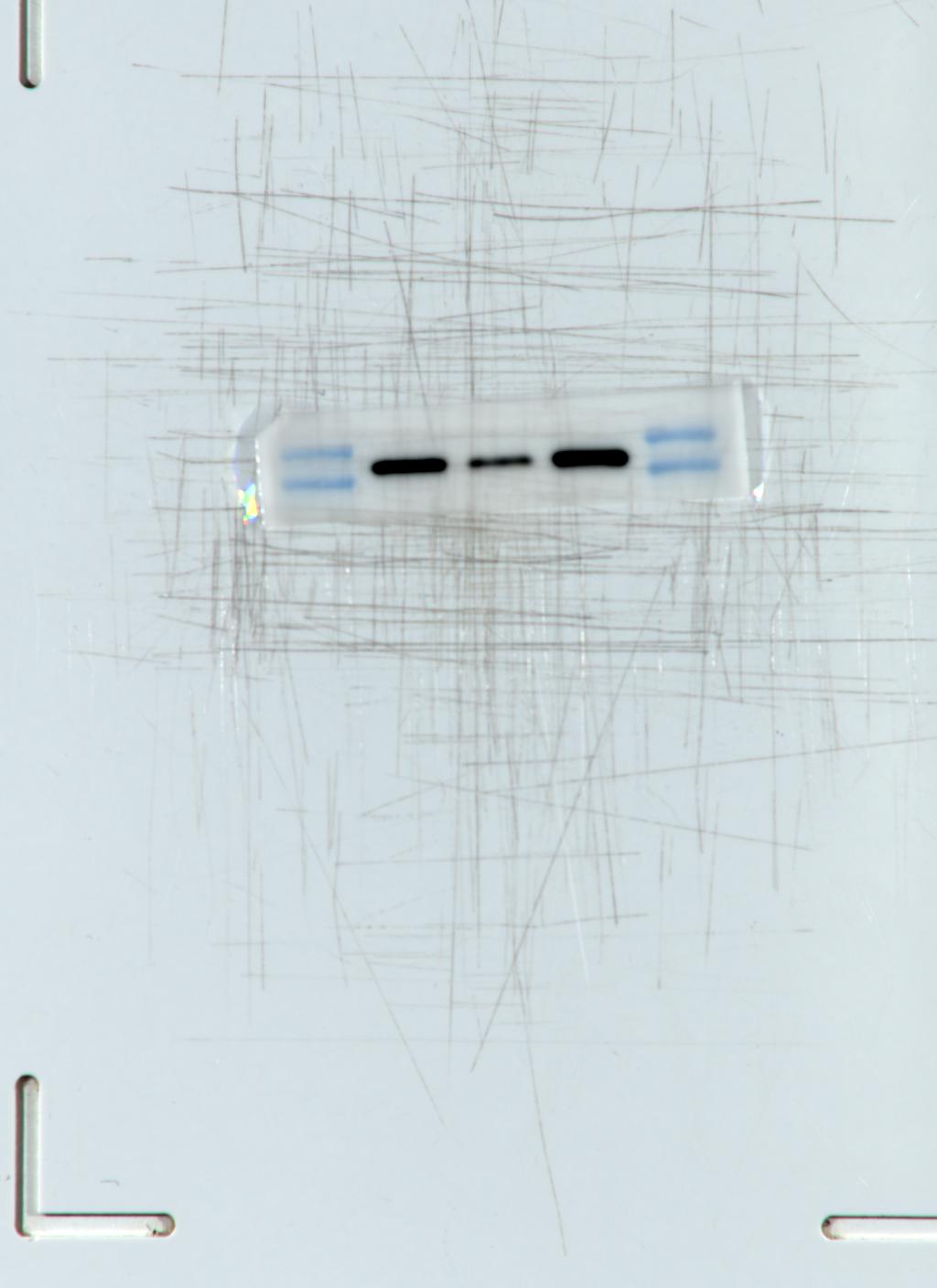

Supplement: Supplementary file 1 [file DataSheet1.ZIP › Raw Data 2/WB/figure 3C/E-ca 7.jpg]

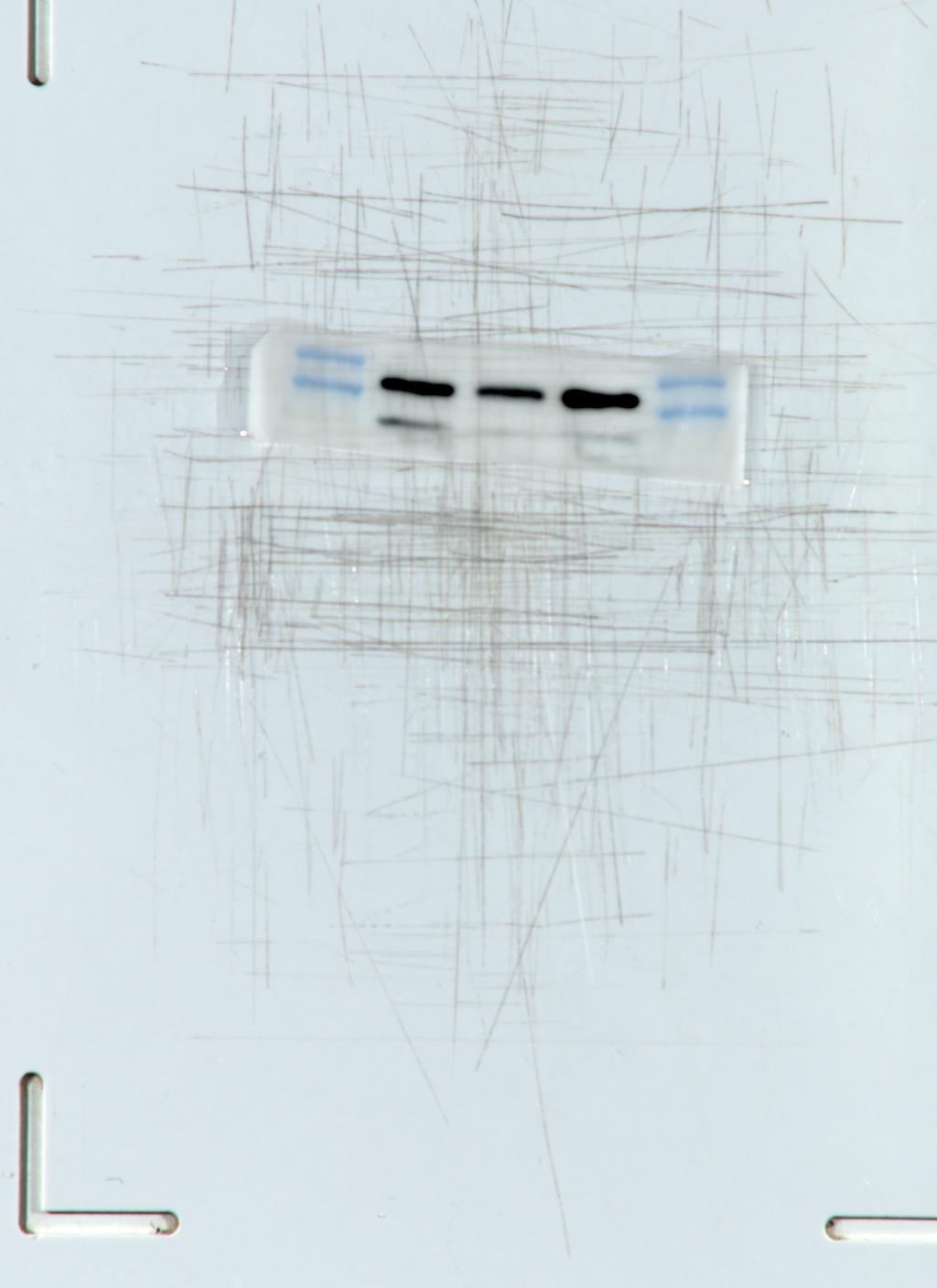

Supplement: Supplementary file 1 [file DataSheet1.ZIP › Raw Data 2/WB/figure 3C/E-ca 8.jpg]

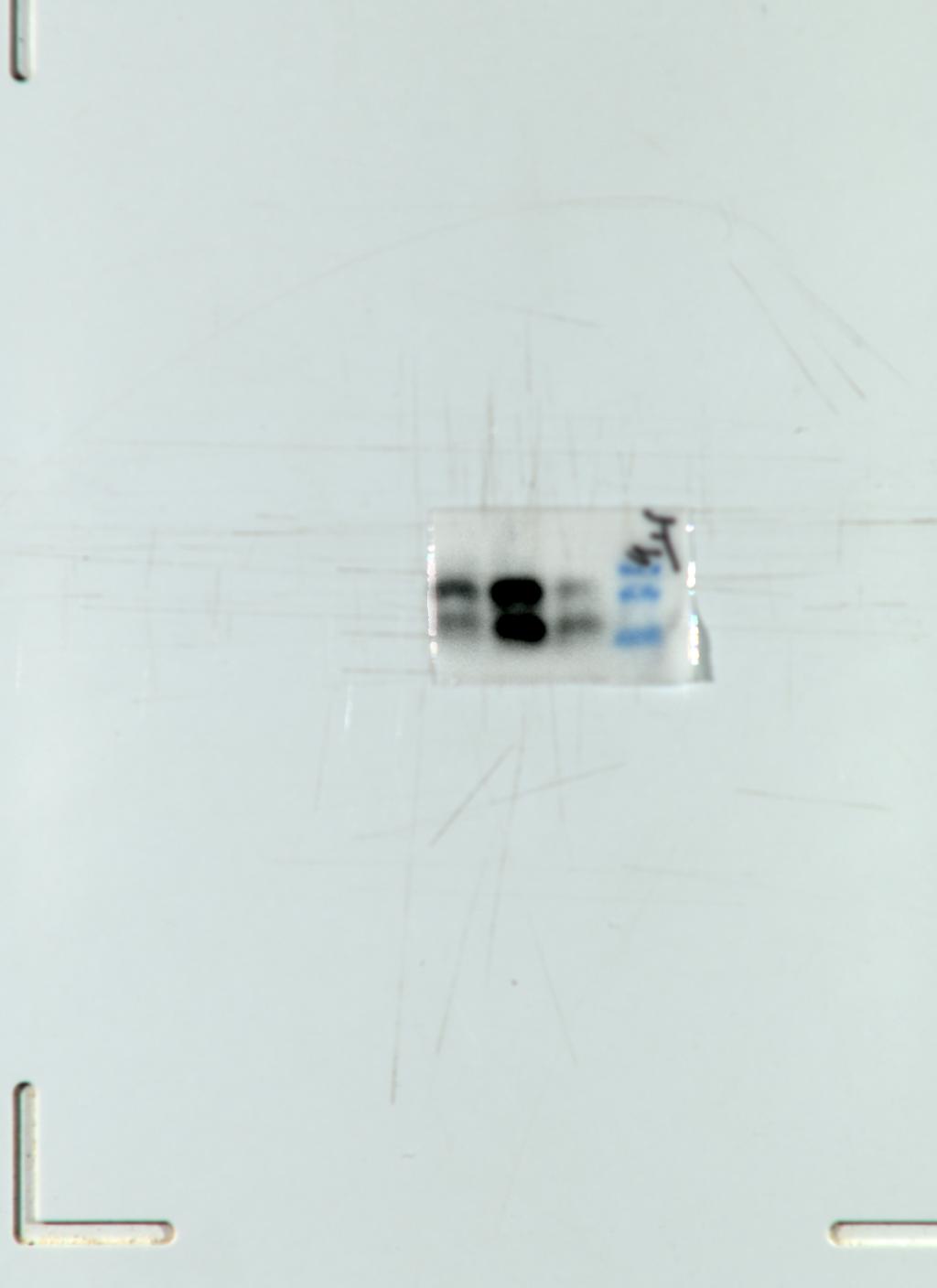

Supplement: Supplementary file 1 [file DataSheet1.ZIP › Raw Data 2/WB/figure 3C/fn 2.jpg]

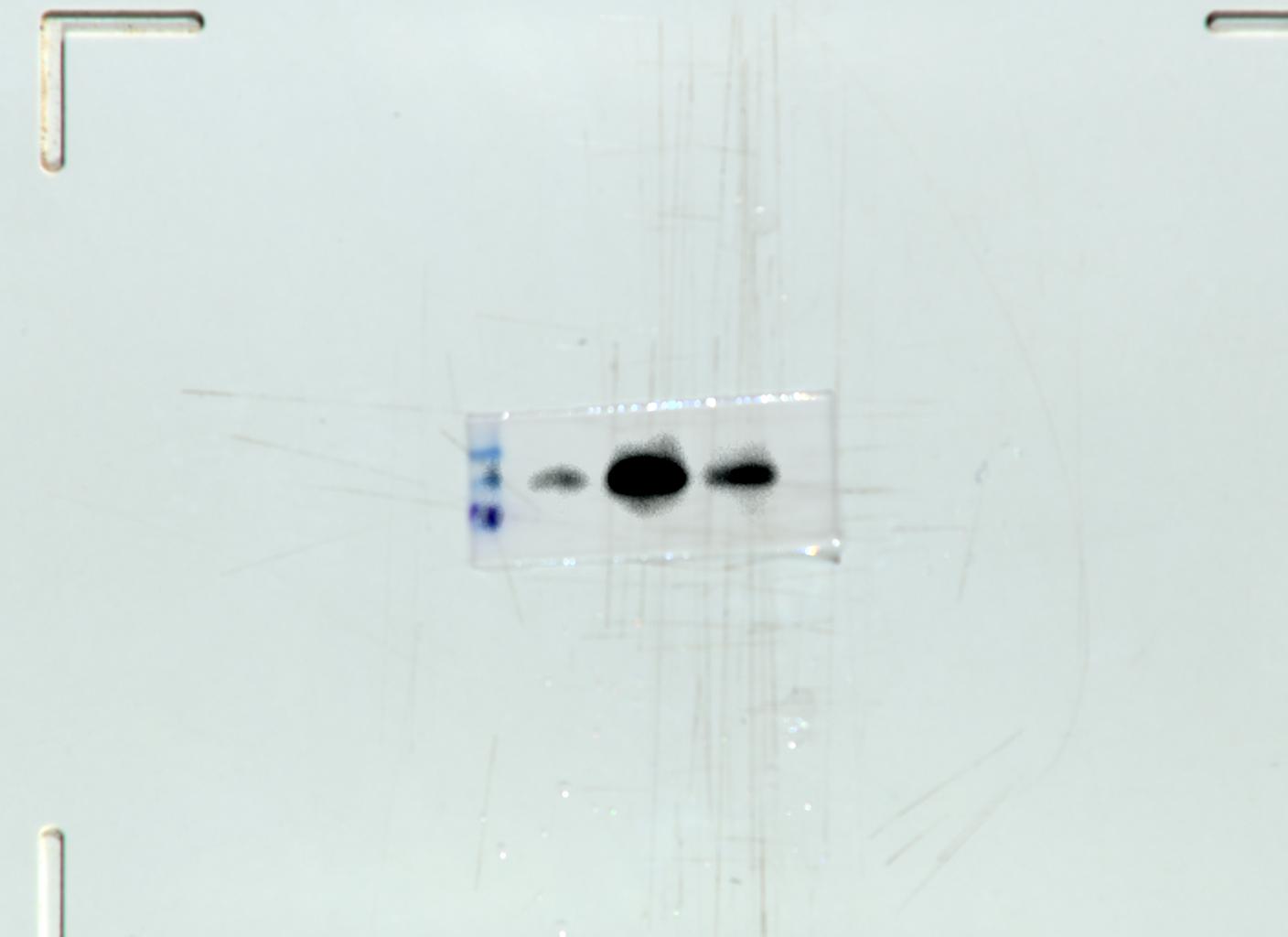

Supplement: Supplementary file 1 [file DataSheet1.ZIP › Raw Data 2/WB/figure 3C/fn 6.jpg]

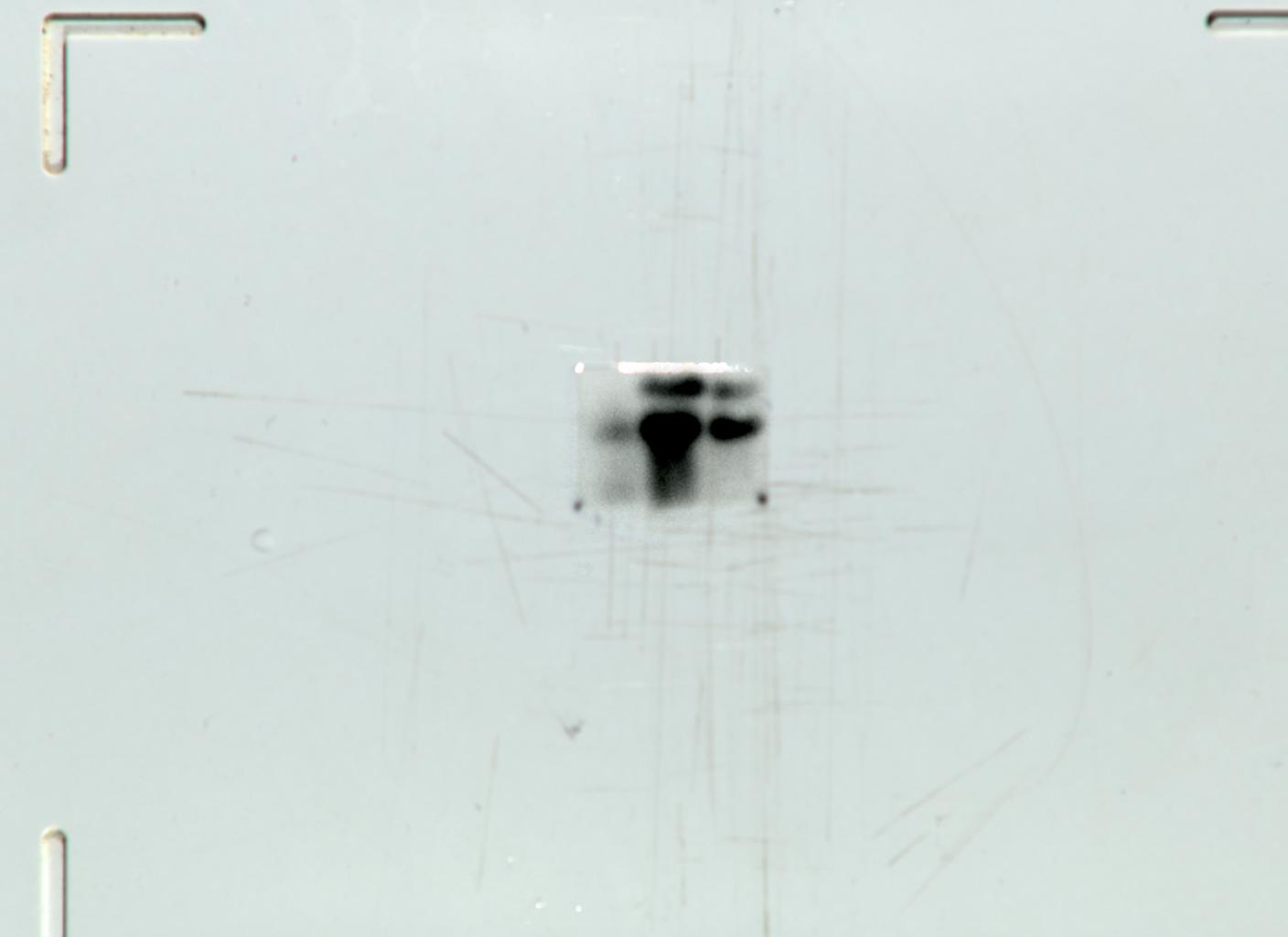

Supplement: Supplementary file 1 [file DataSheet1.ZIP › Raw Data 2/WB/figure 3C/fn 3.jpg]

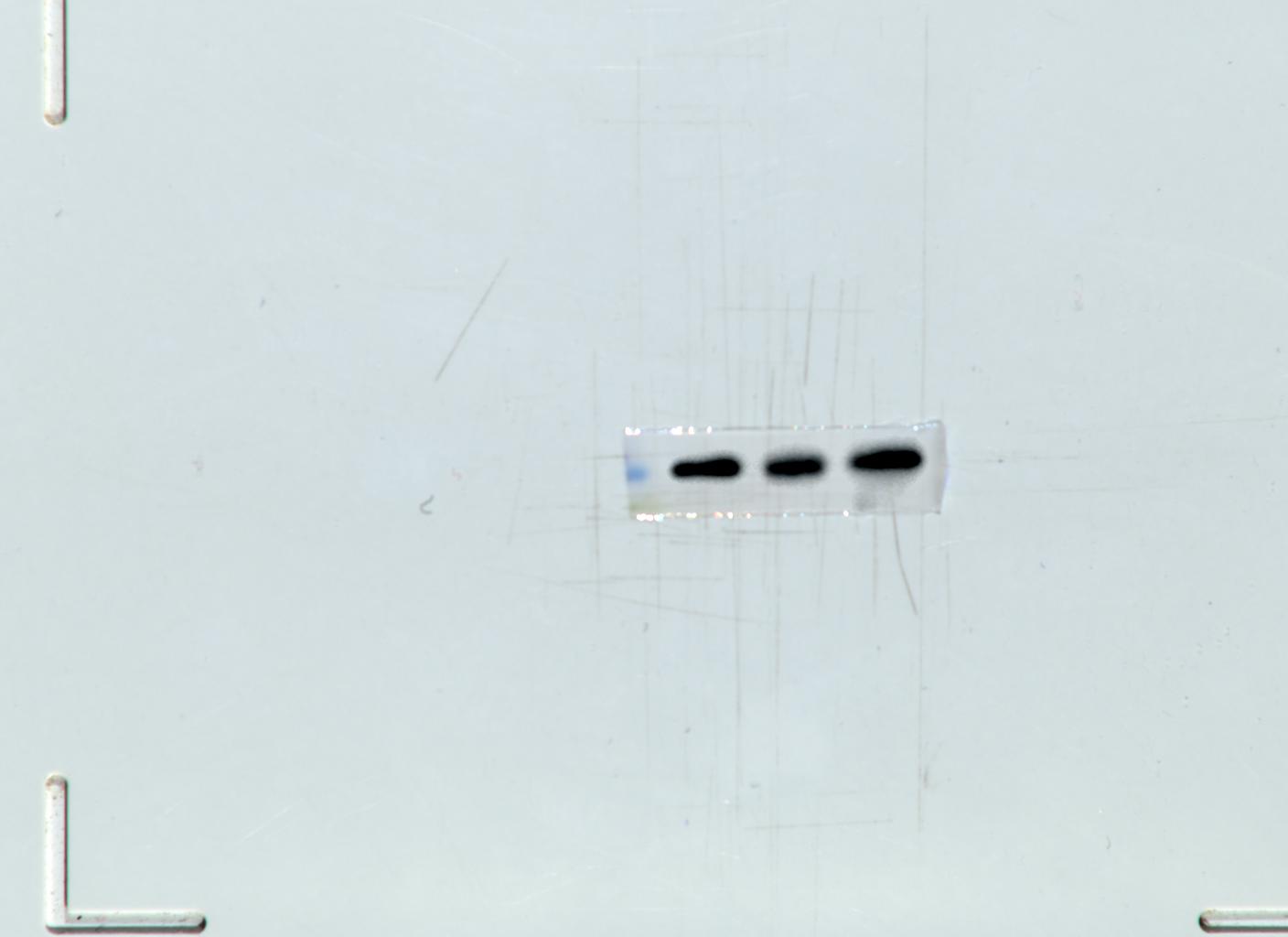

Supplement: Supplementary file 1 [file DataSheet1.ZIP › Raw Data 2/WB/figure 3C/gap 6.jpg]

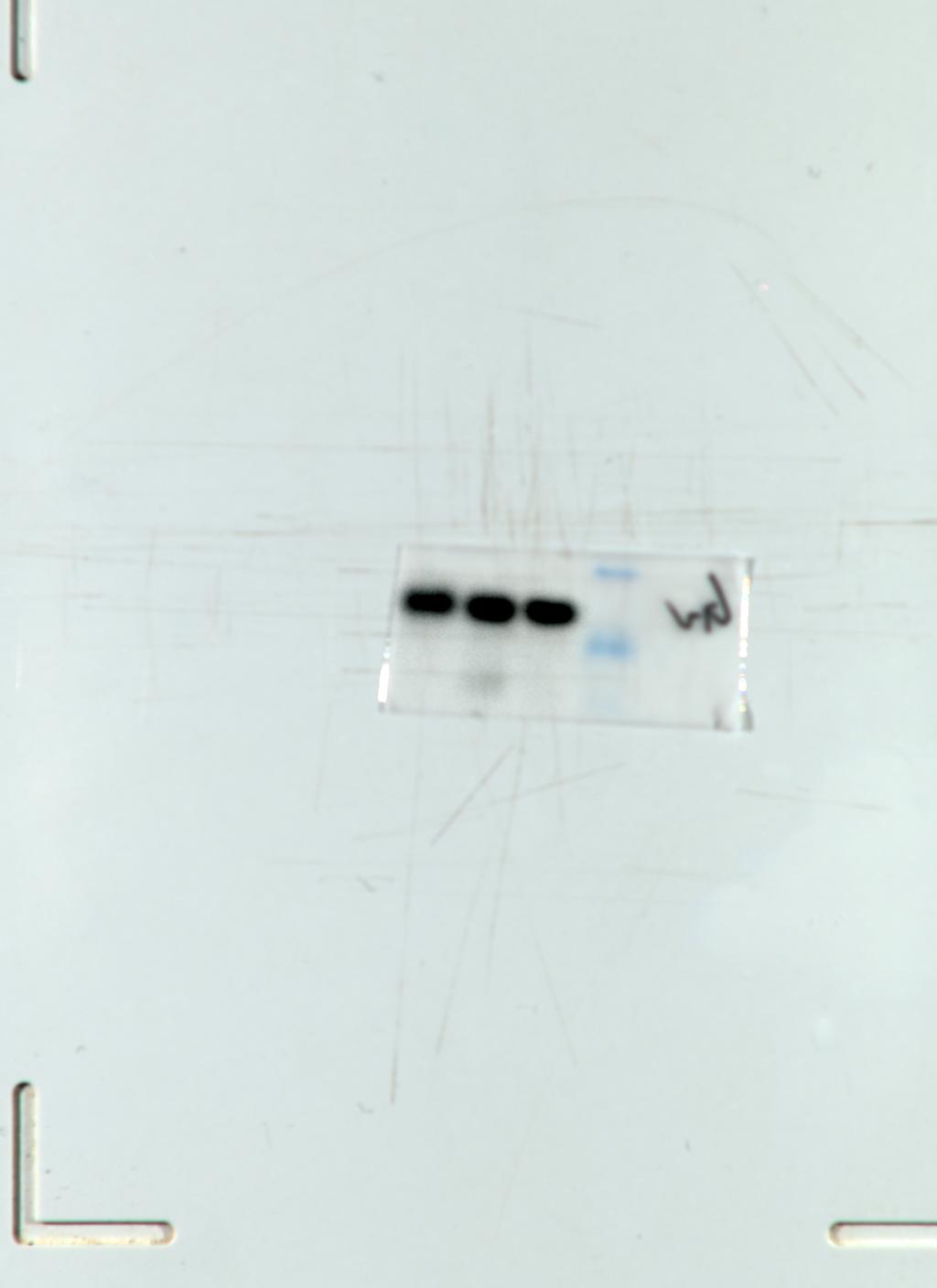

Supplement: Supplementary file 1 [file DataSheet1.ZIP › Raw Data 2/WB/figure 3C/gap 2.jpg]

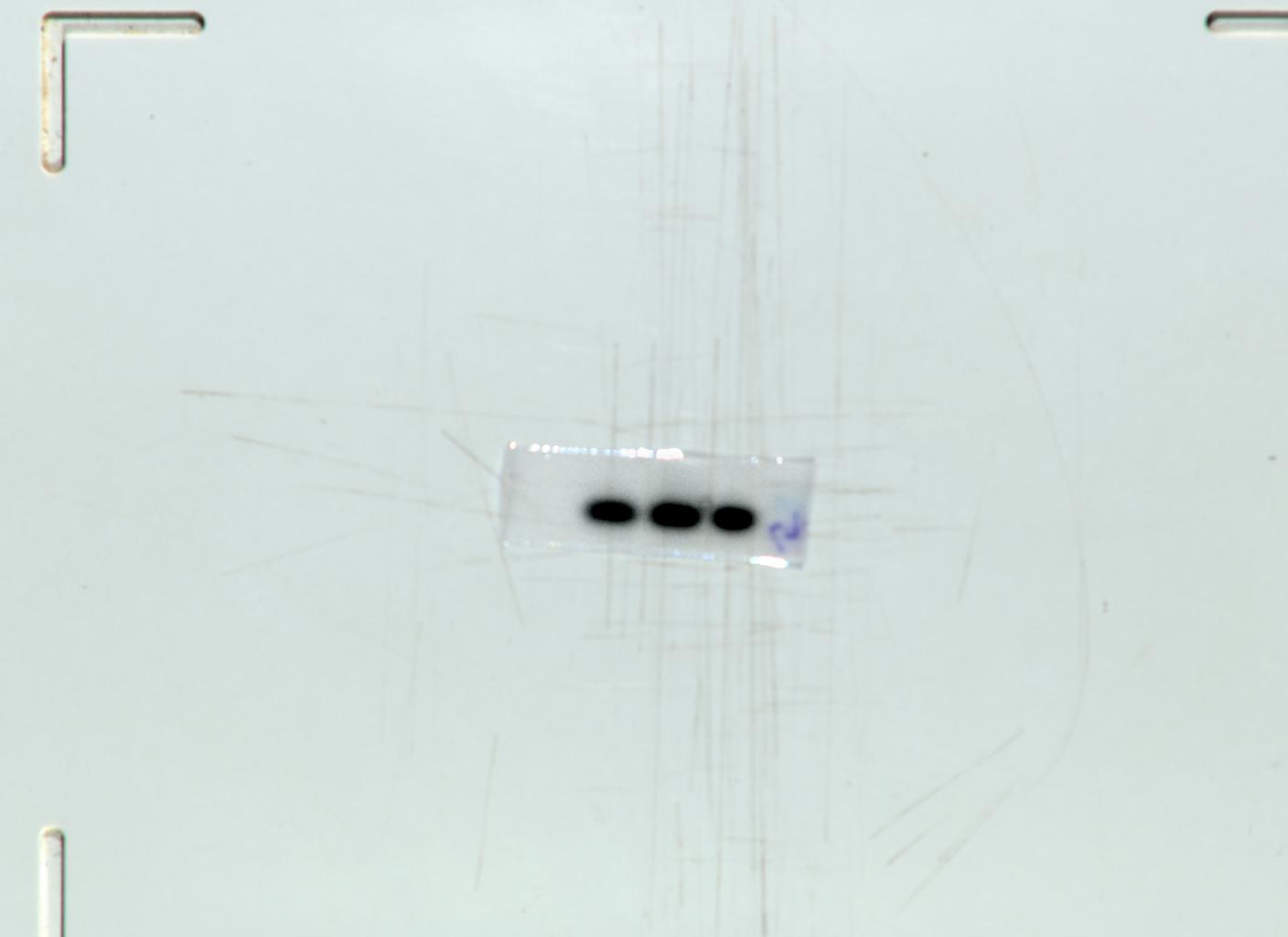

Supplement: Supplementary file 1 [file DataSheet1.ZIP › Raw Data 2/WB/figure 3C/gap 3.jpg]

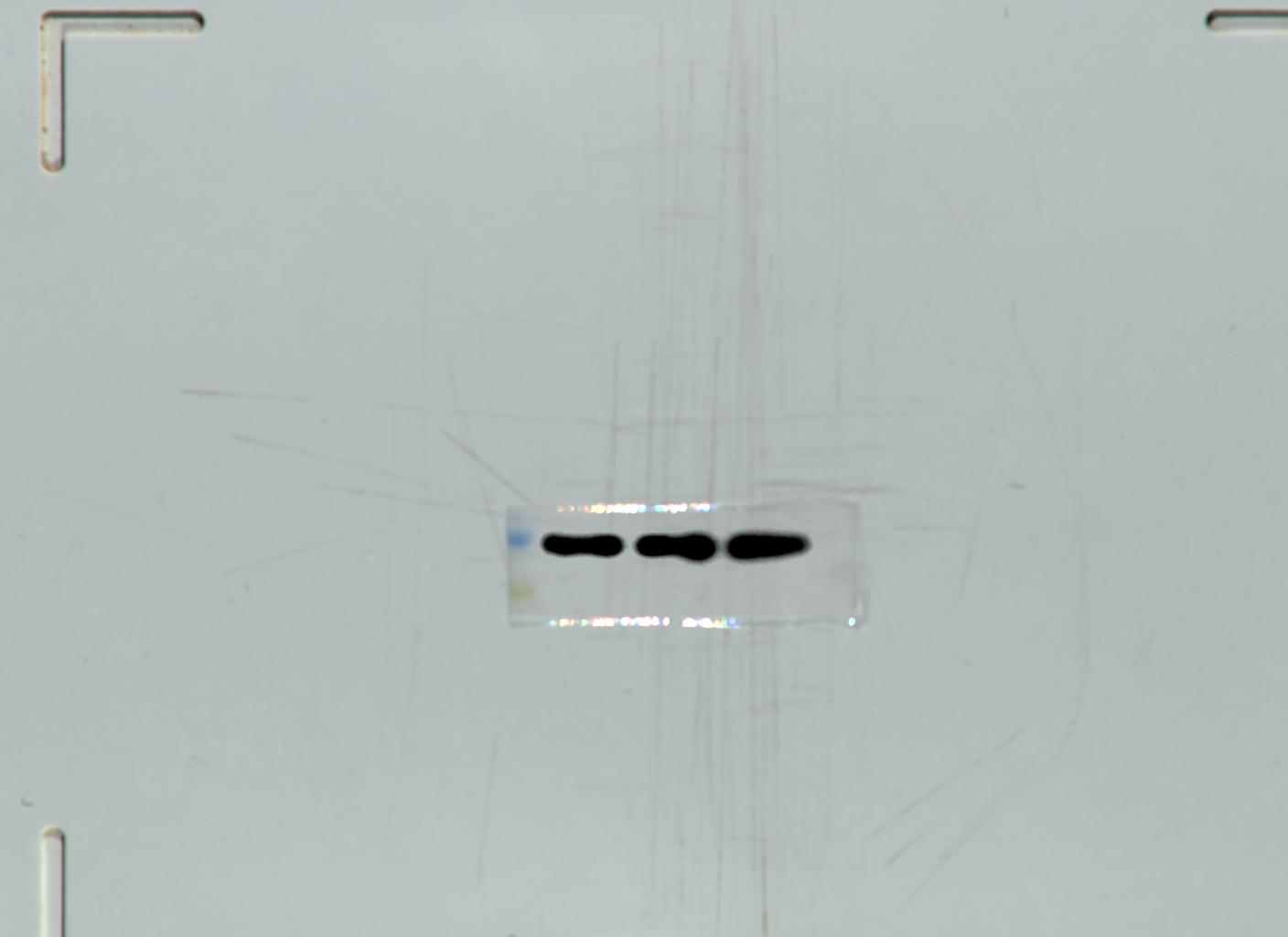

Supplement: Supplementary file 1 [file DataSheet1.ZIP › Raw Data 2/WB/figure 3C/gap 5.jpg]

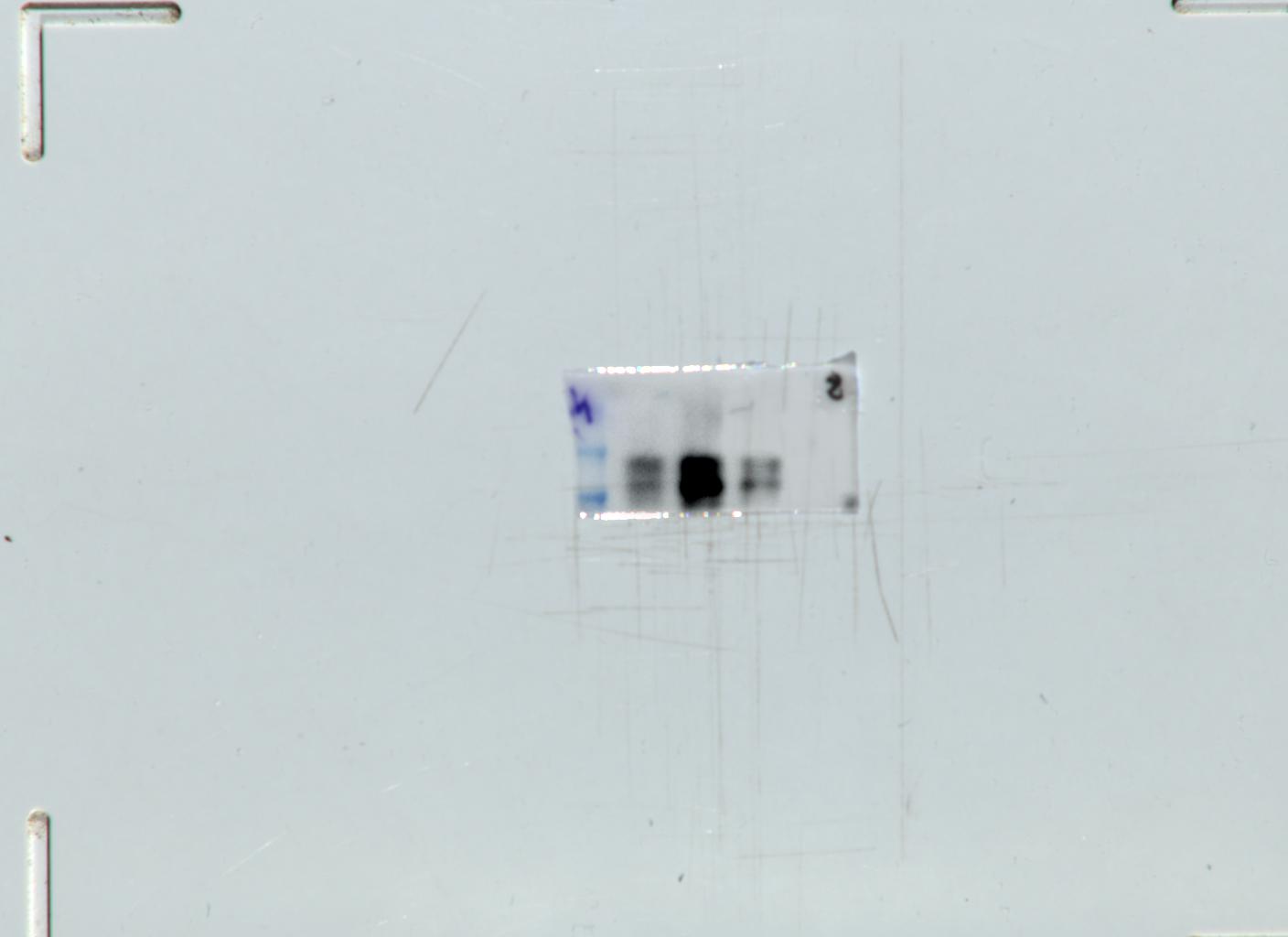

Supplement: Supplementary file 1 [file DataSheet1.ZIP › Raw Data 2/WB/figure 3C/lu-nca 1.jpg]

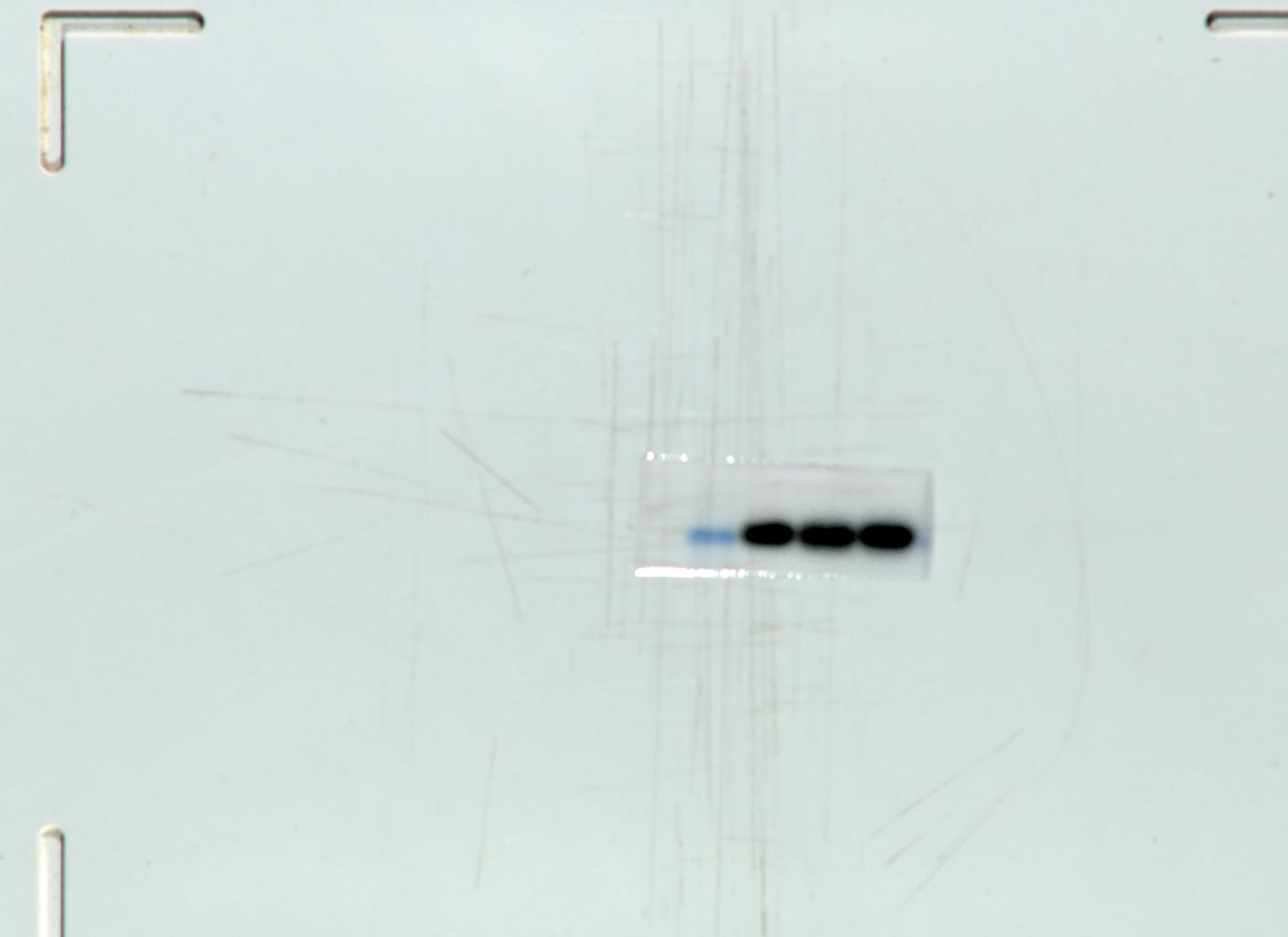

Supplement: Supplementary file 1 [file DataSheet1.ZIP › Raw Data 2/WB/figure 3C/luj-gap 1.jpg]

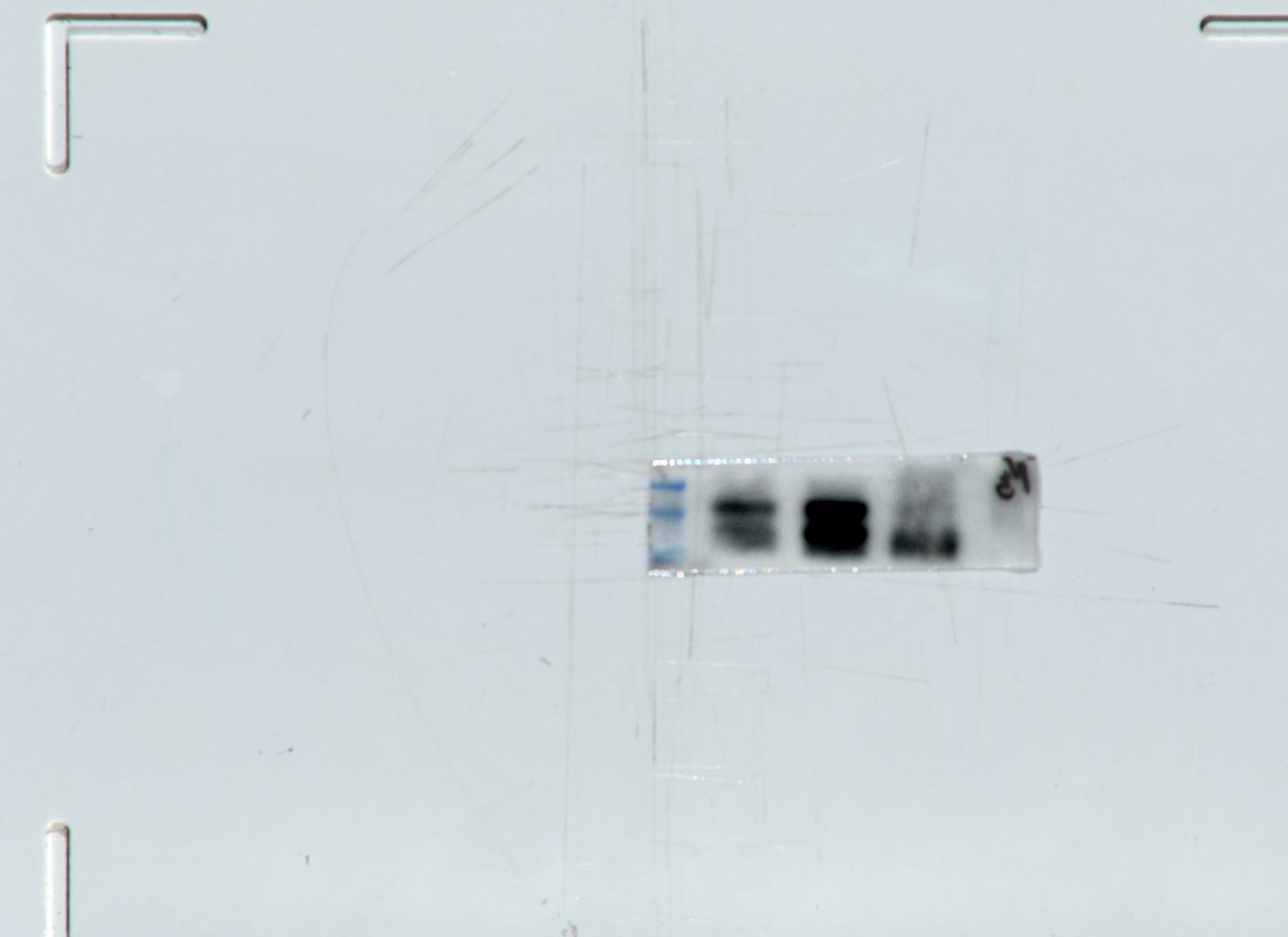

Supplement: Supplementary file 1 [file DataSheet1.ZIP › Raw Data 2/WB/figure 3C/nca 4.jpg]

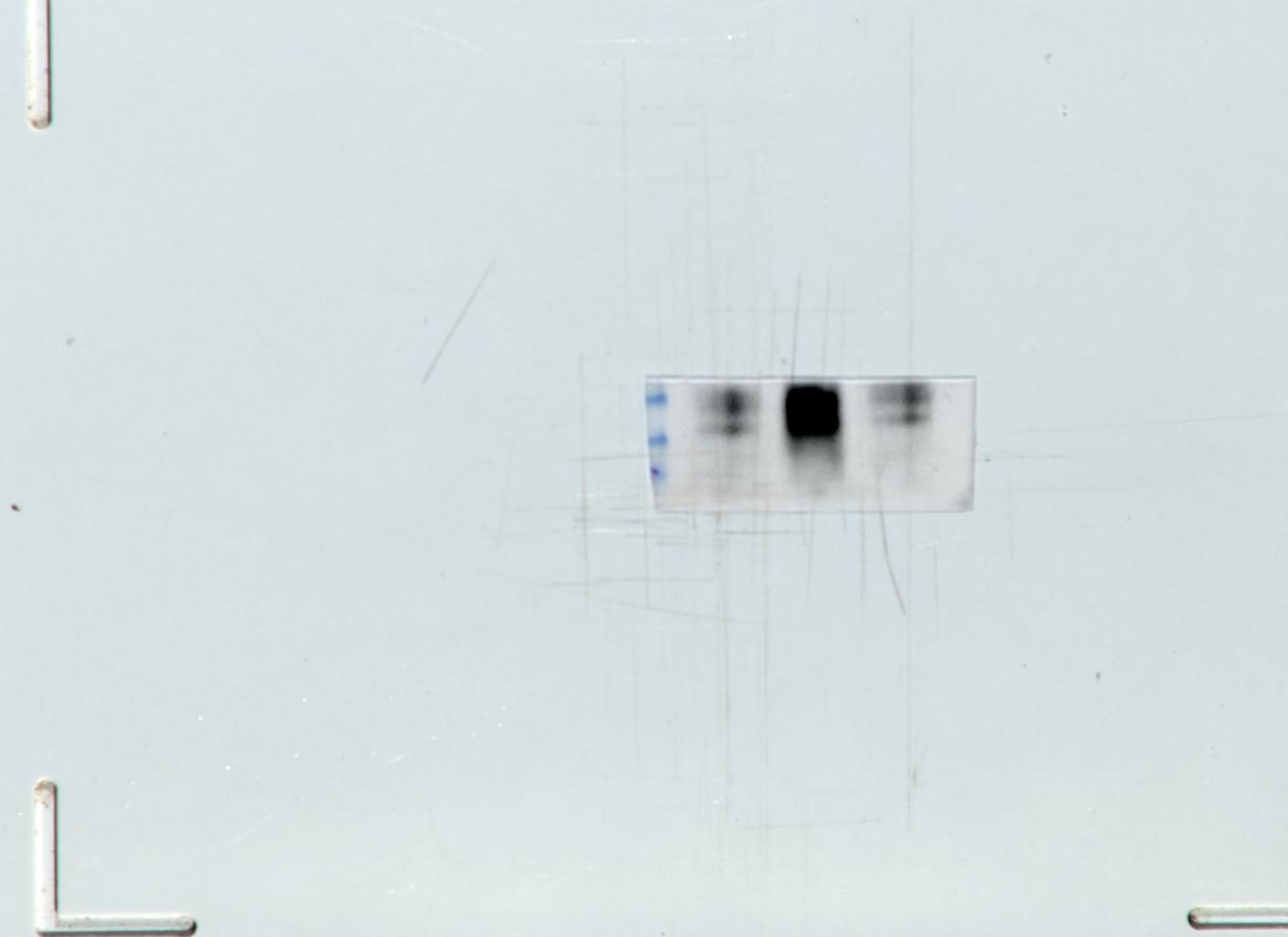

Supplement: Supplementary file 1 [file DataSheet1.ZIP › Raw Data 2/WB/figure 3C/nca 5.jpg]

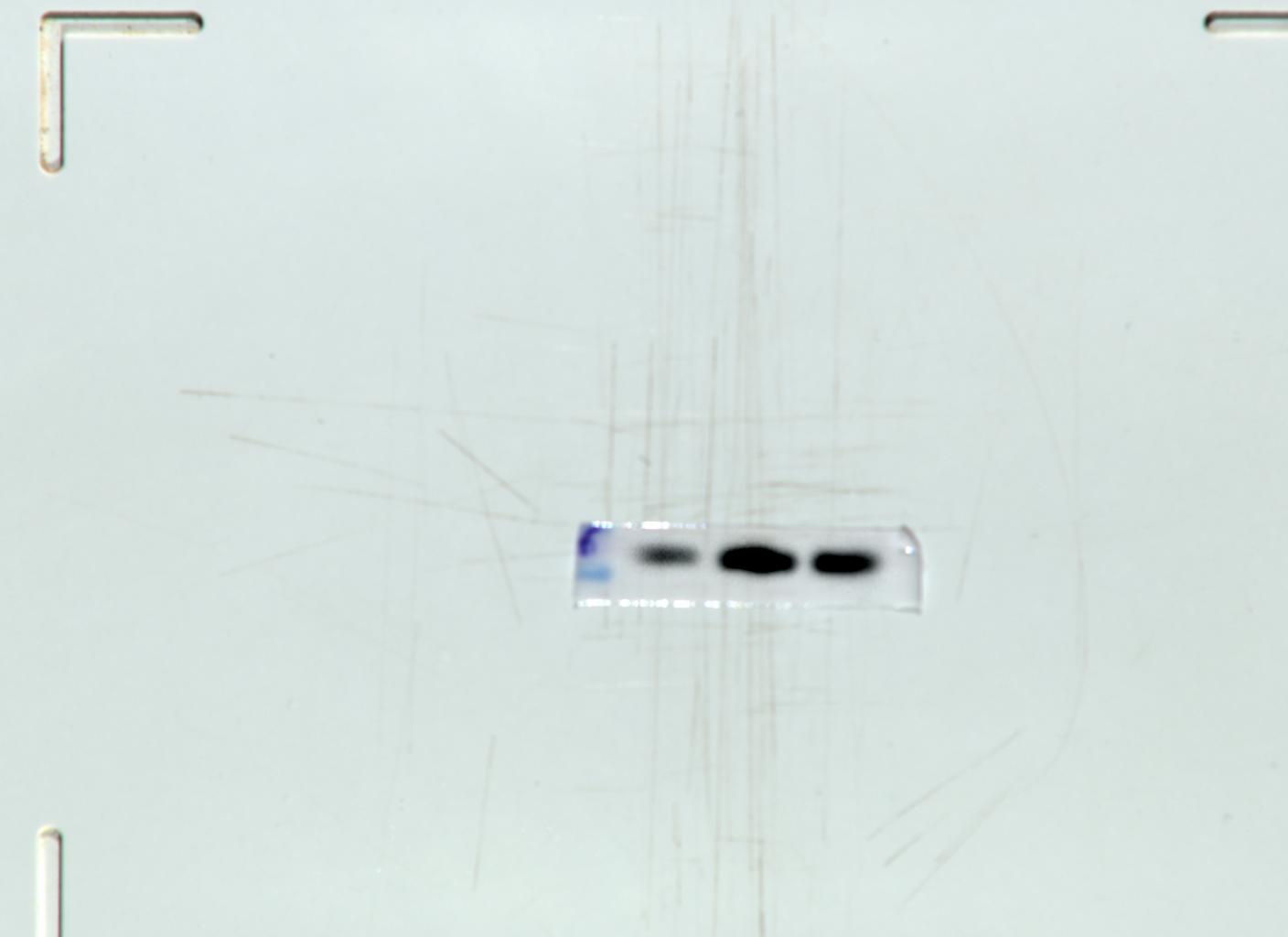

Supplement: Supplementary file 1 [file DataSheet1.ZIP › Raw Data 2/WB/figure 3C/sma 6.jpg]

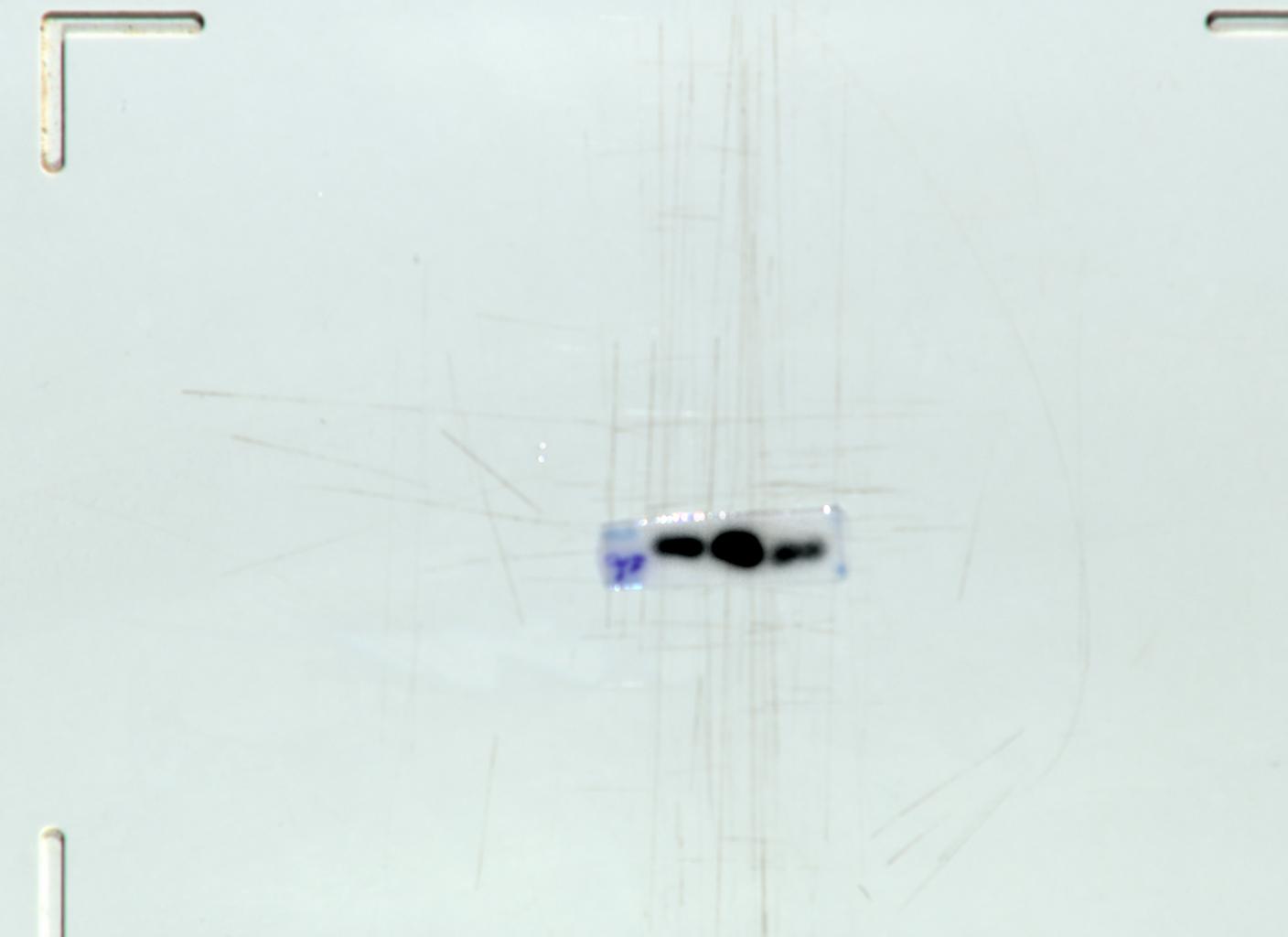

Supplement: Supplementary file 1 [file DataSheet1.ZIP › Raw Data 2/WB/figure 3C/sma 3.jpg]

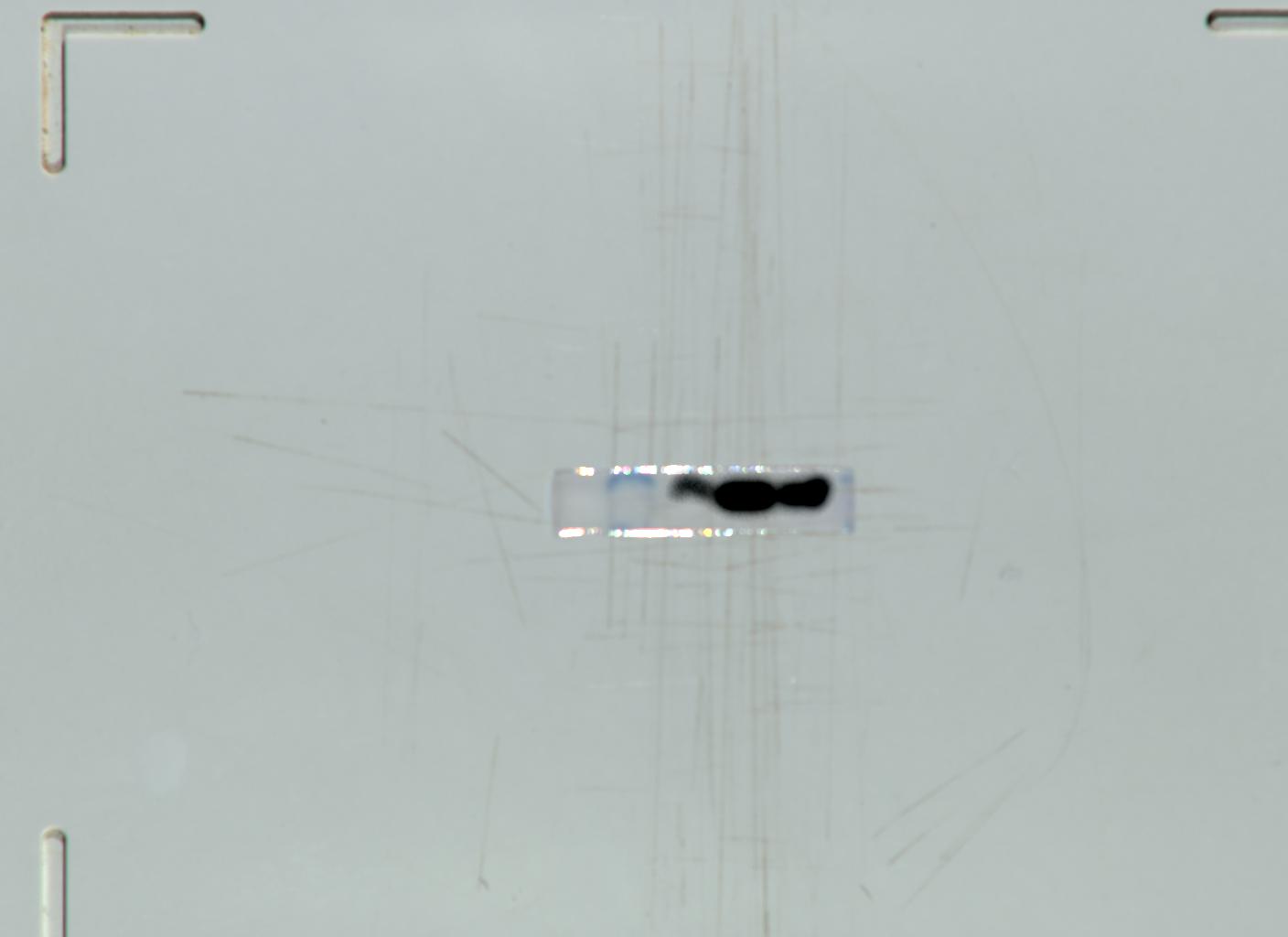

Supplement: Supplementary file 1 [file DataSheet1.ZIP › Raw Data 2/WB/figure 3C/sma 2.jpg]

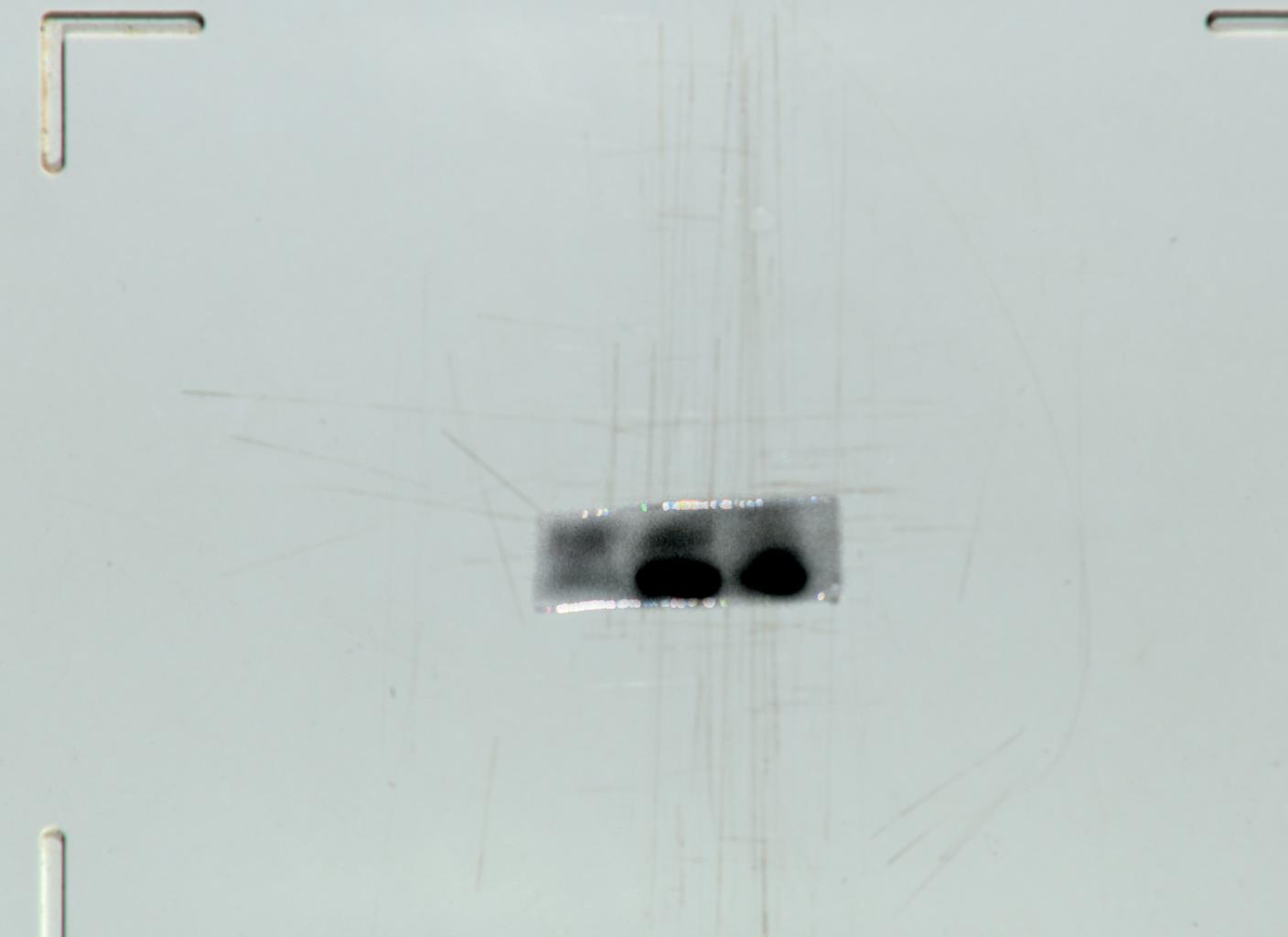

Supplement: Supplementary file 1 [file DataSheet1.ZIP › Raw Data 2/WB/figure 3C/vim 6.jpg]

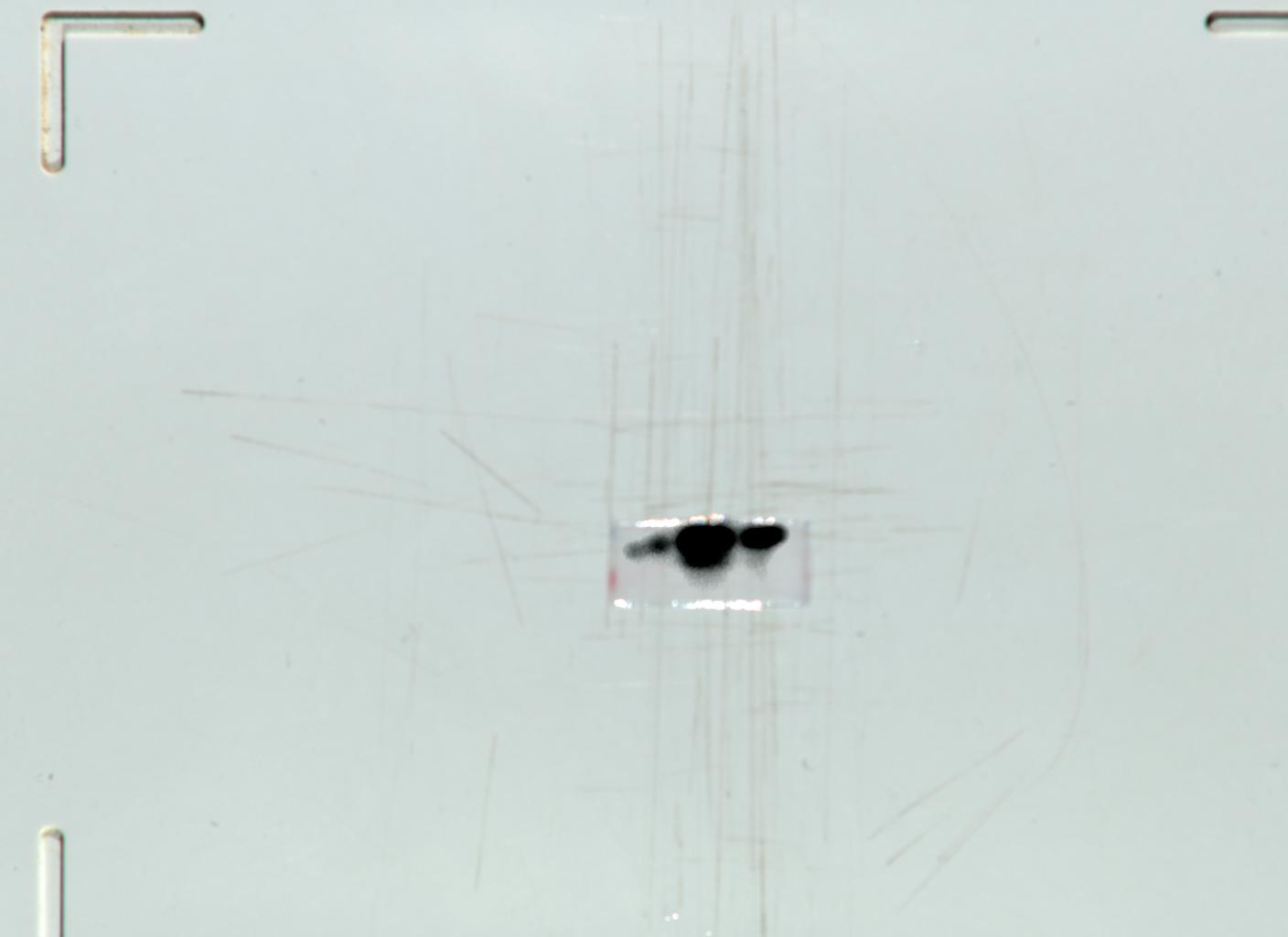

Supplement: Supplementary file 1 [file DataSheet1.ZIP › Raw Data 2/WB/figure 3C/vim 3.jpg]

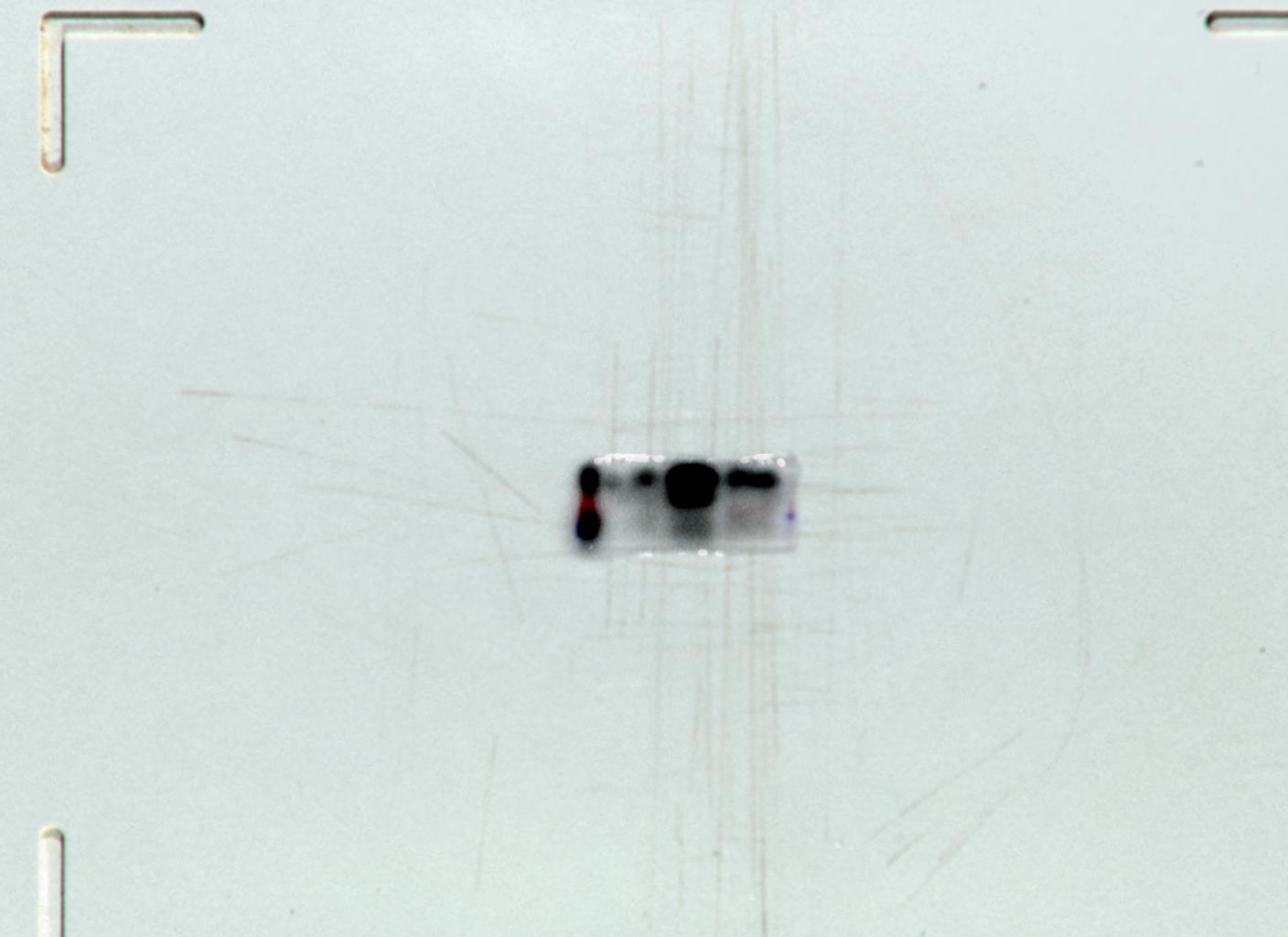

Supplement: Supplementary file 1 [file DataSheet1.ZIP › Raw Data 2/WB/figure 3C/vim 2.jpg]

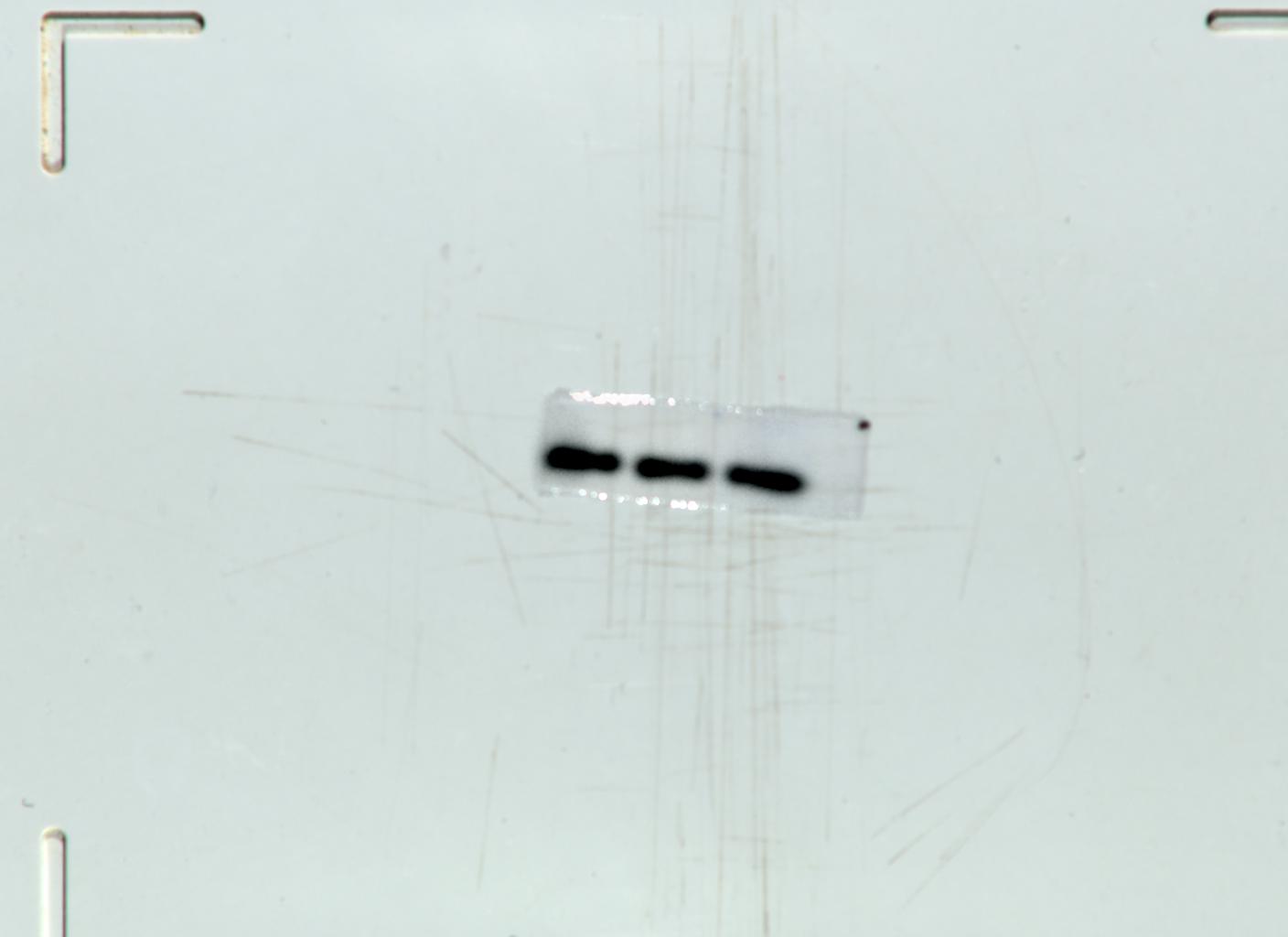

Supplement: Supplementary file 1 [file DataSheet1.ZIP › Raw Data 2/WB/figure 4A/gap 4.jpg]

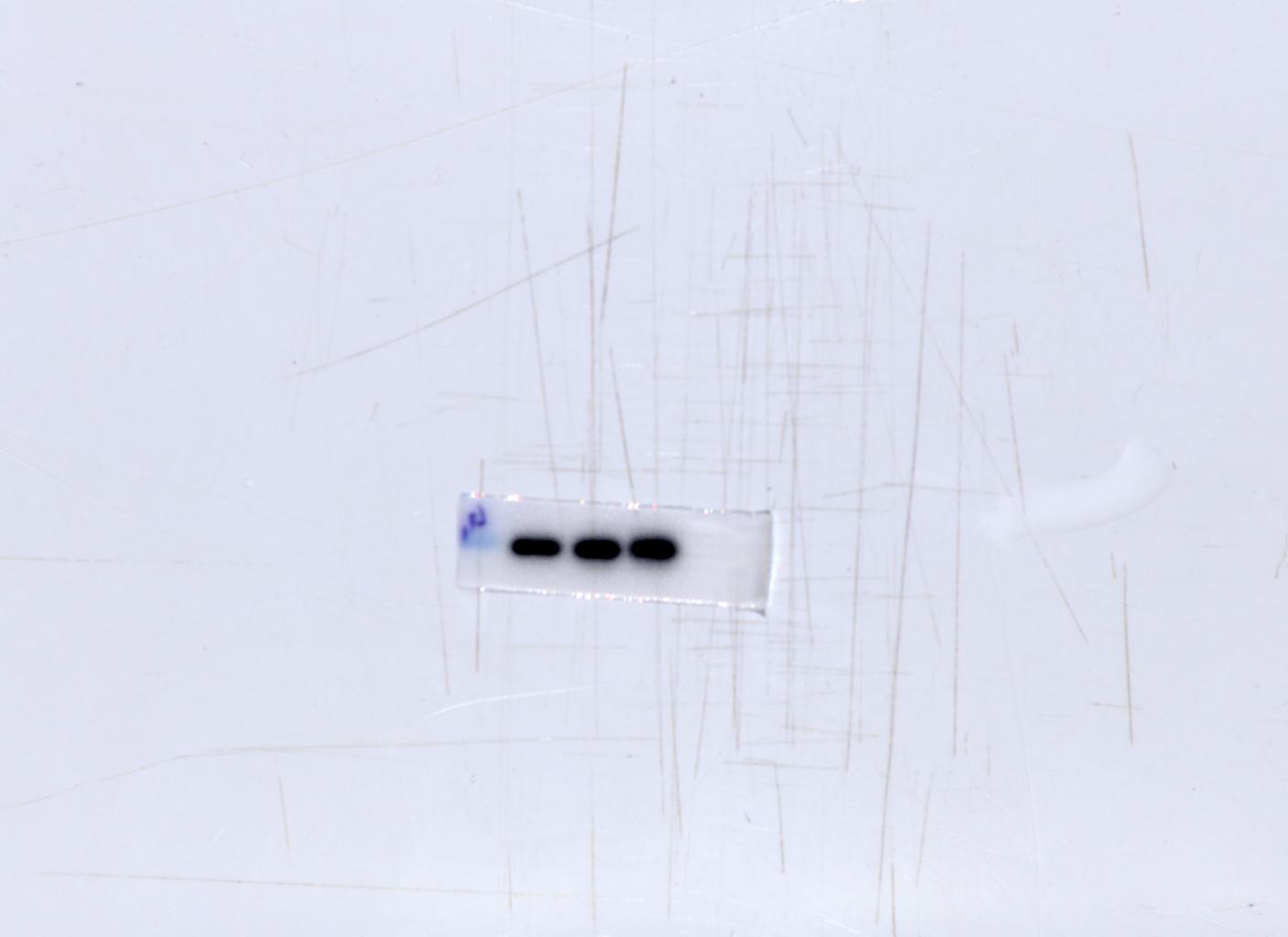

Supplement: Supplementary file 1 [file DataSheet1.ZIP › Raw Data 2/WB/figure 4A/gap 8.jpg]

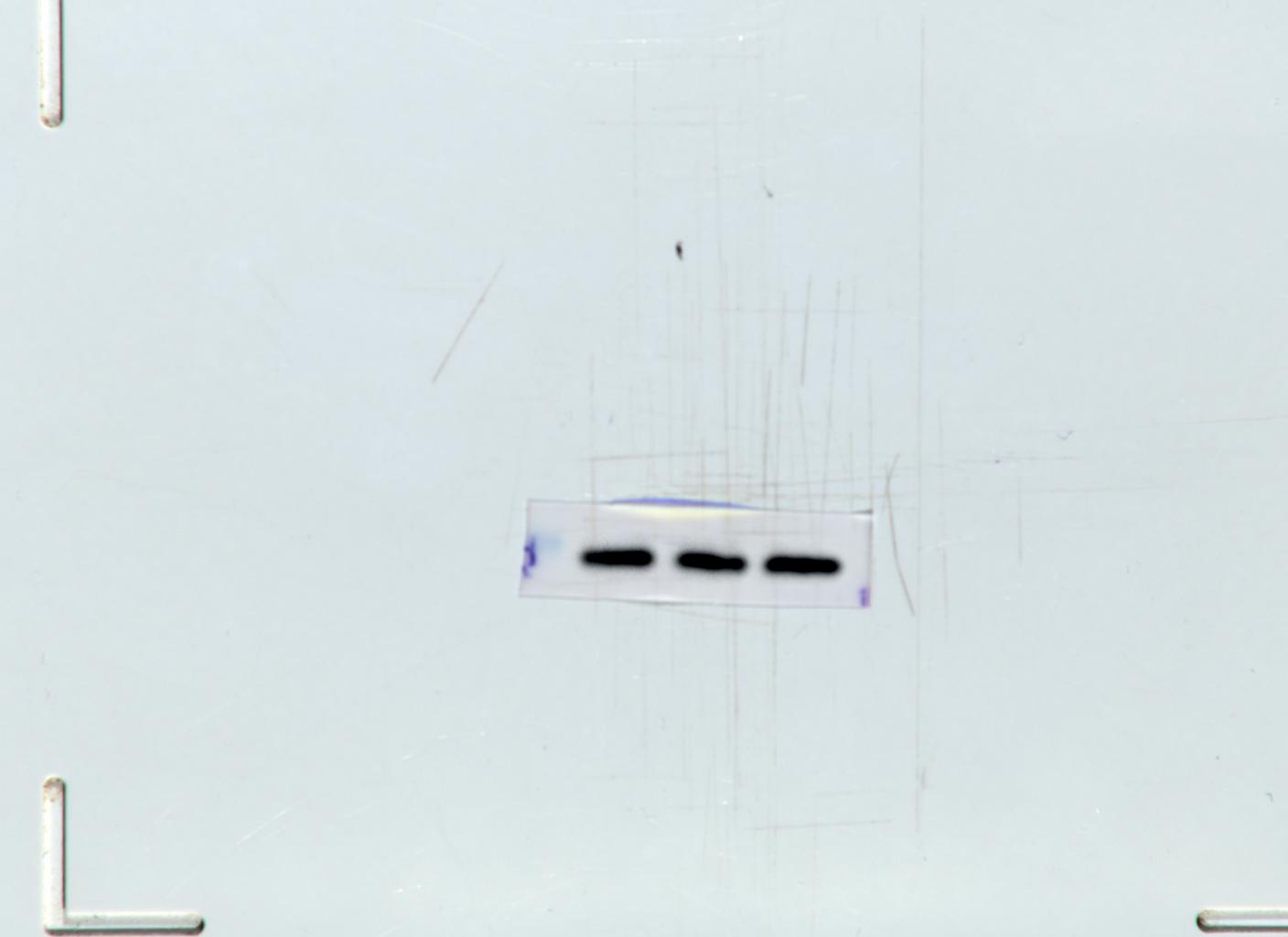

Supplement: Supplementary file 1 [file DataSheet1.ZIP › Raw Data 2/WB/figure 4A/gap 10.jpg]

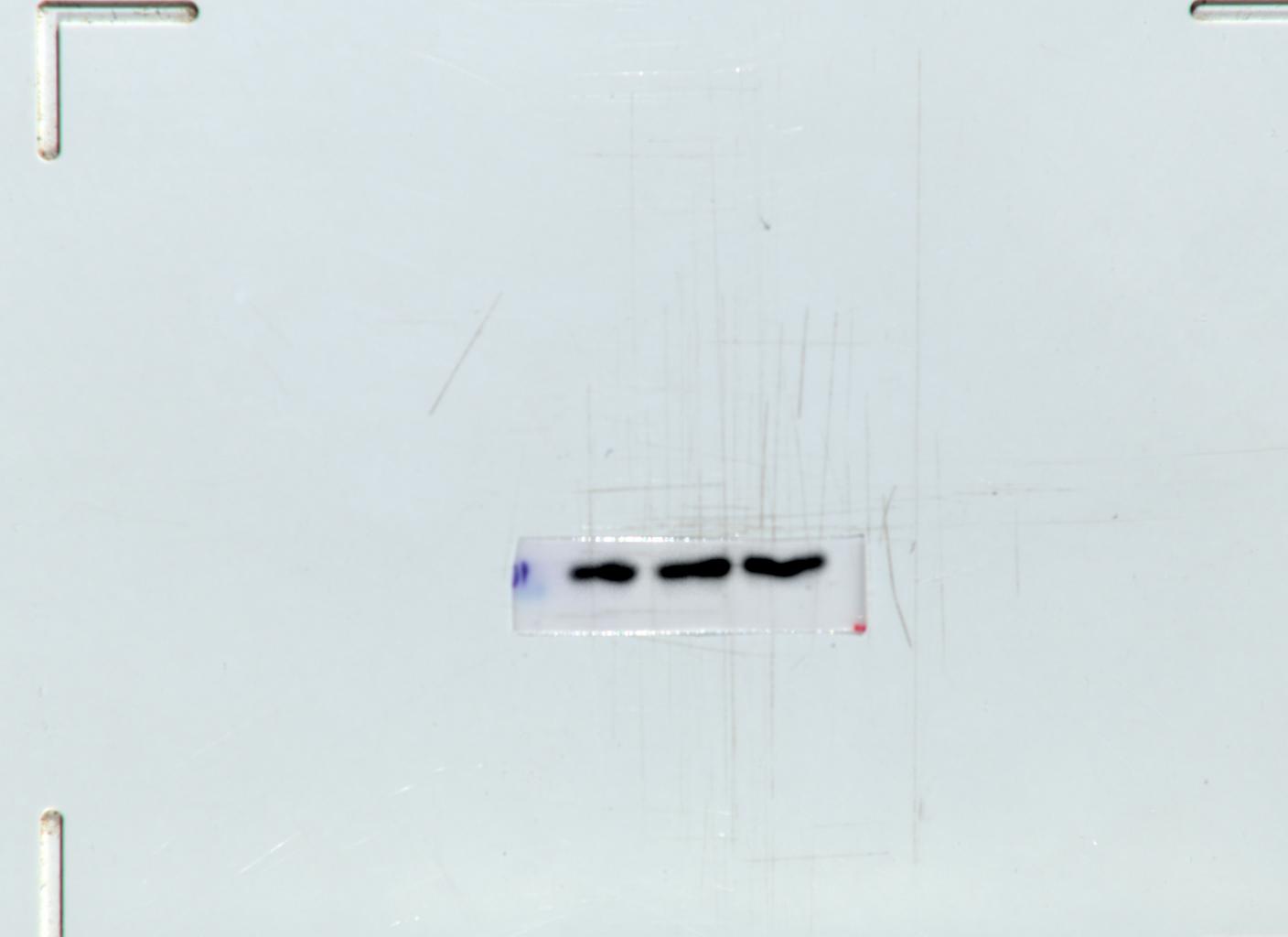

Supplement: Supplementary file 1 [file DataSheet1.ZIP › Raw Data 2/WB/figure 4A/gap 9.jpg]

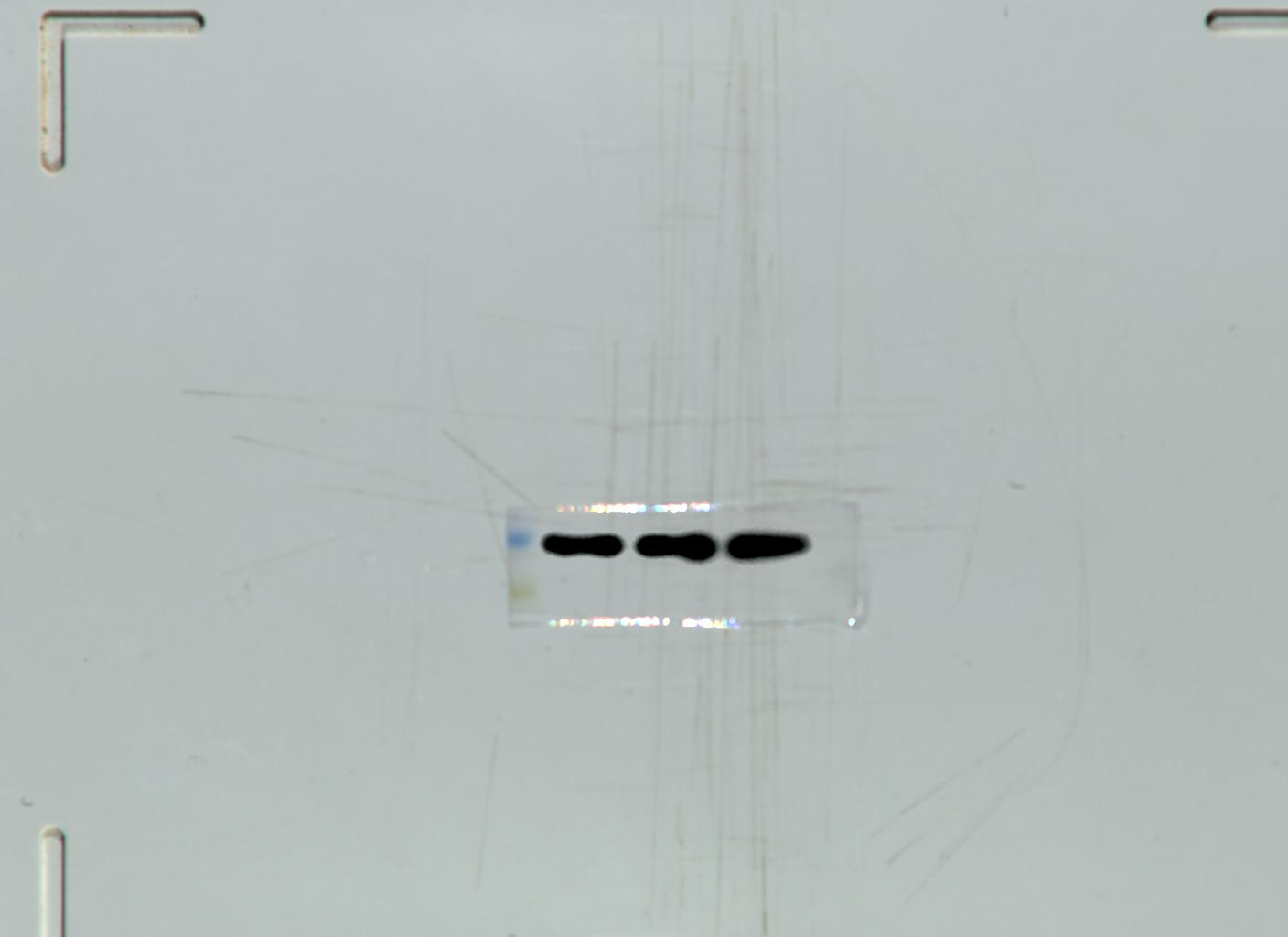

Supplement: Supplementary file 1 [file DataSheet1.ZIP › Raw Data 2/WB/figure 4A/gap 5.jpg]

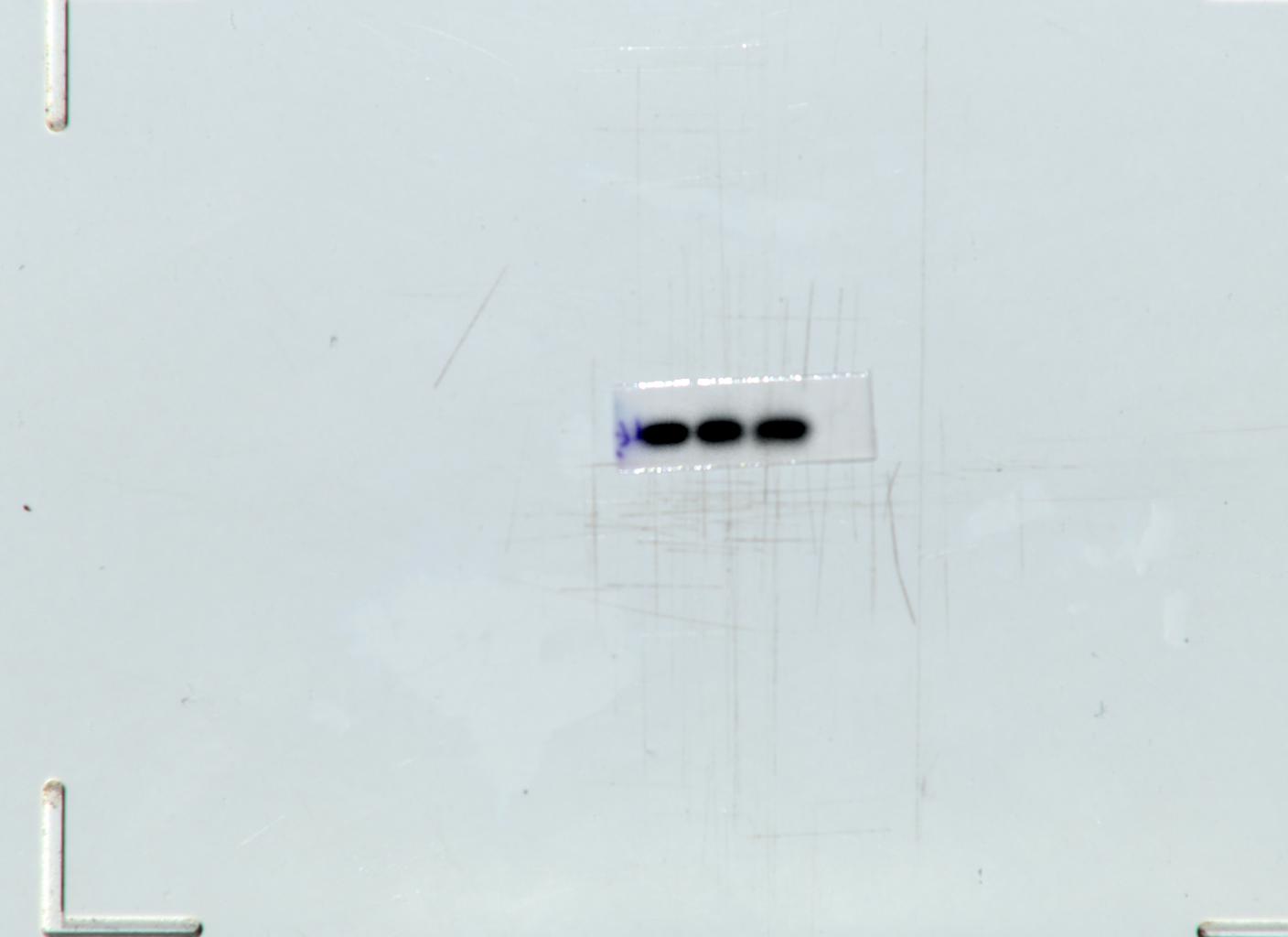

Supplement: Supplementary file 1 [file DataSheet1.ZIP › Raw Data 2/WB/figure 4A/gap 7.jpg]

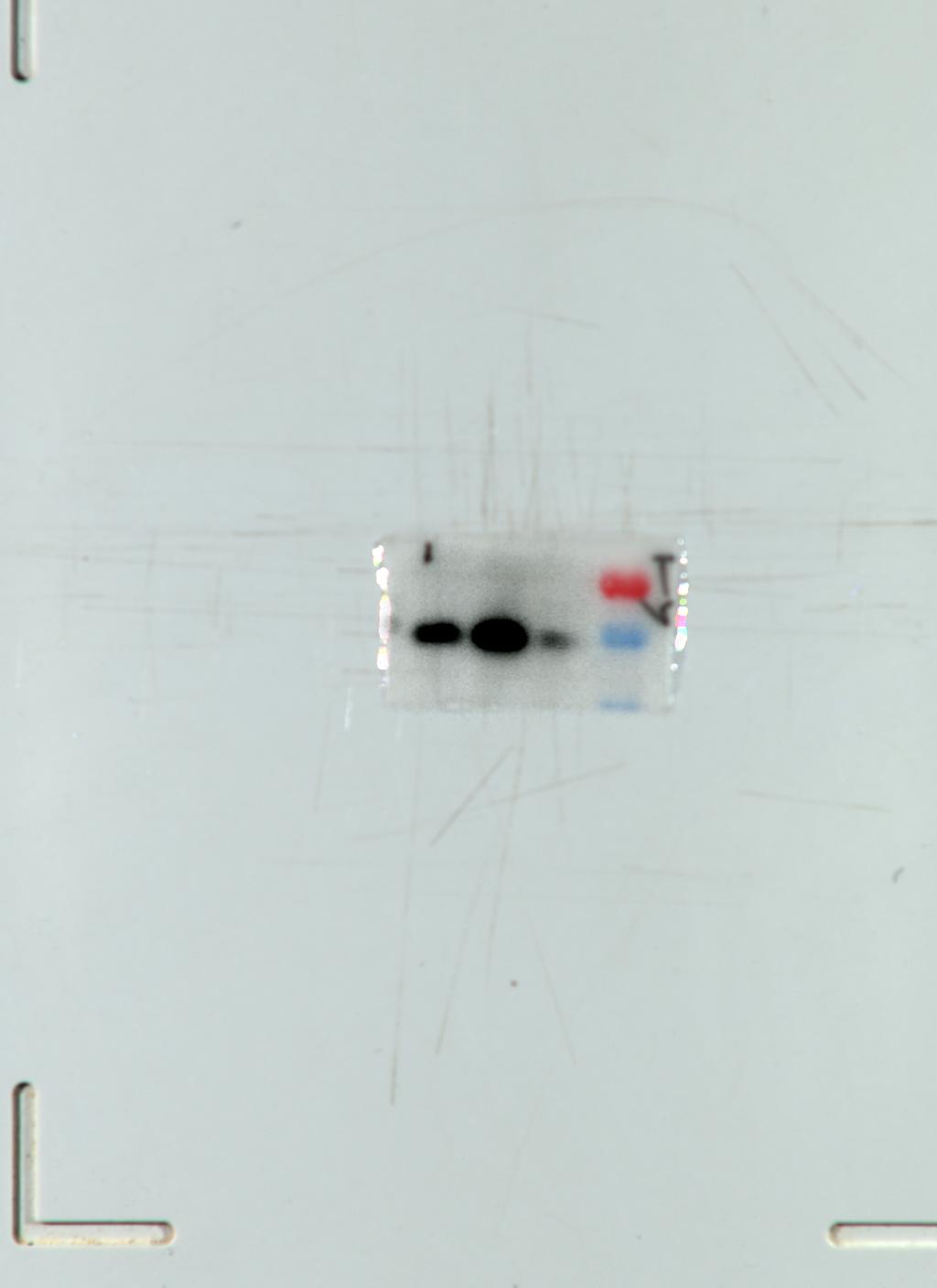

Supplement: Supplementary file 1 [file DataSheet1.ZIP › Raw Data 2/WB/figure 4A/lu-tgf 1.jpg]

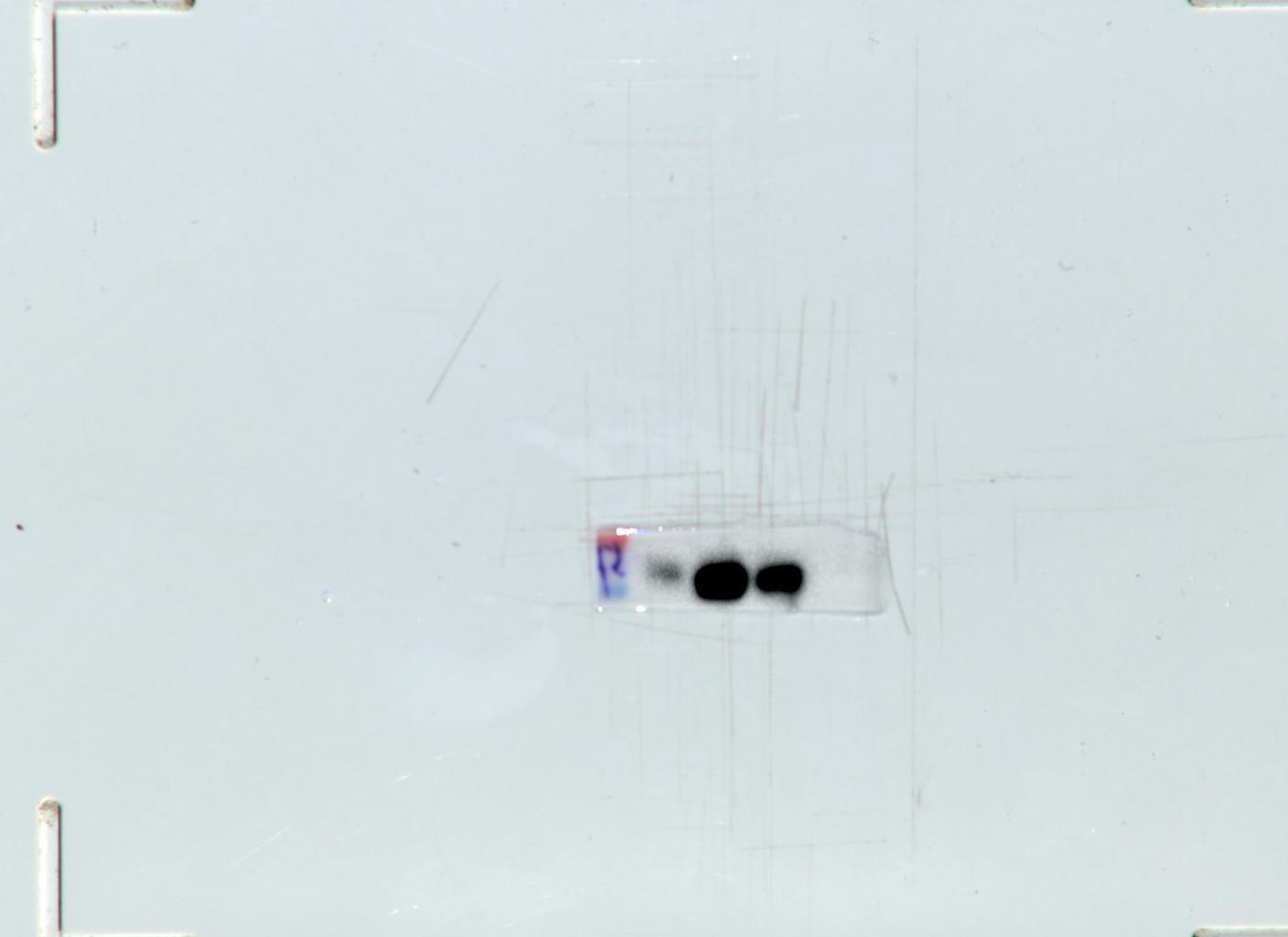

Supplement: Supplementary file 1 [file DataSheet1.ZIP › Raw Data 2/WB/figure 4A/psmad 2 7.jpg]

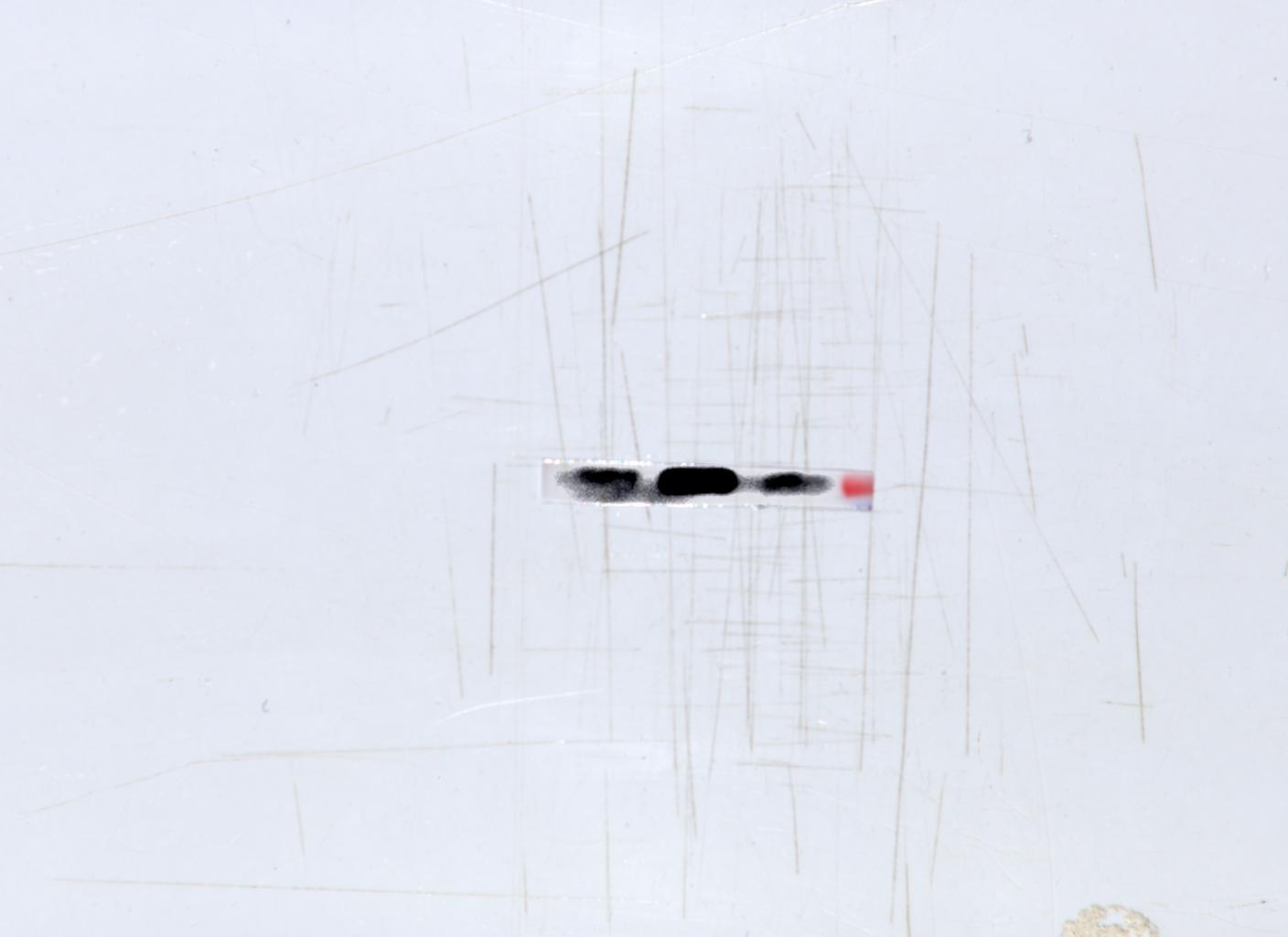

Supplement: Supplementary file 1 [file DataSheet1.ZIP › Raw Data 2/WB/figure 4A/psmad2 9.jpg]

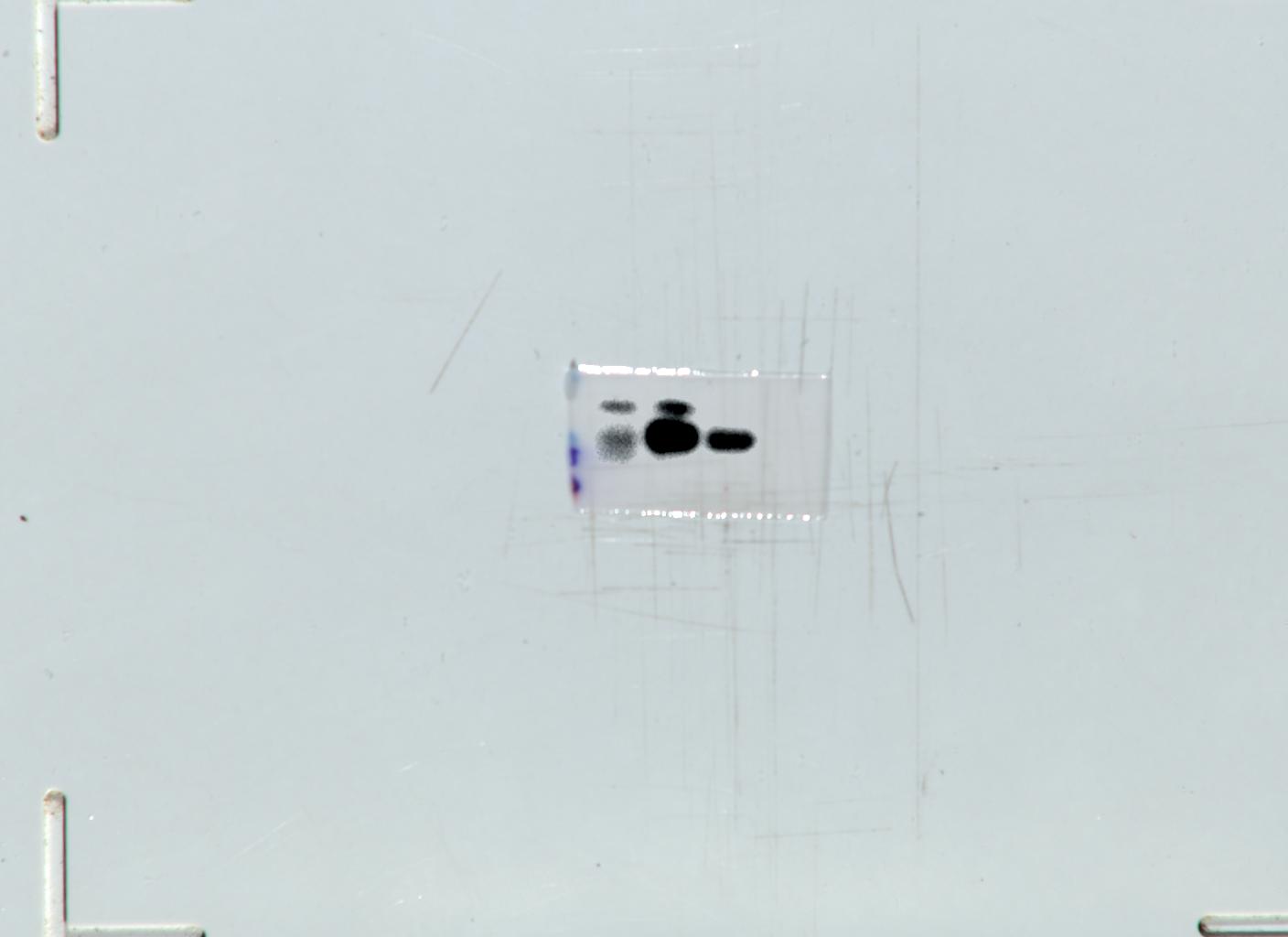

Supplement: Supplementary file 1 [file DataSheet1.ZIP › Raw Data 2/WB/figure 4A/psmad2 10.jpg]

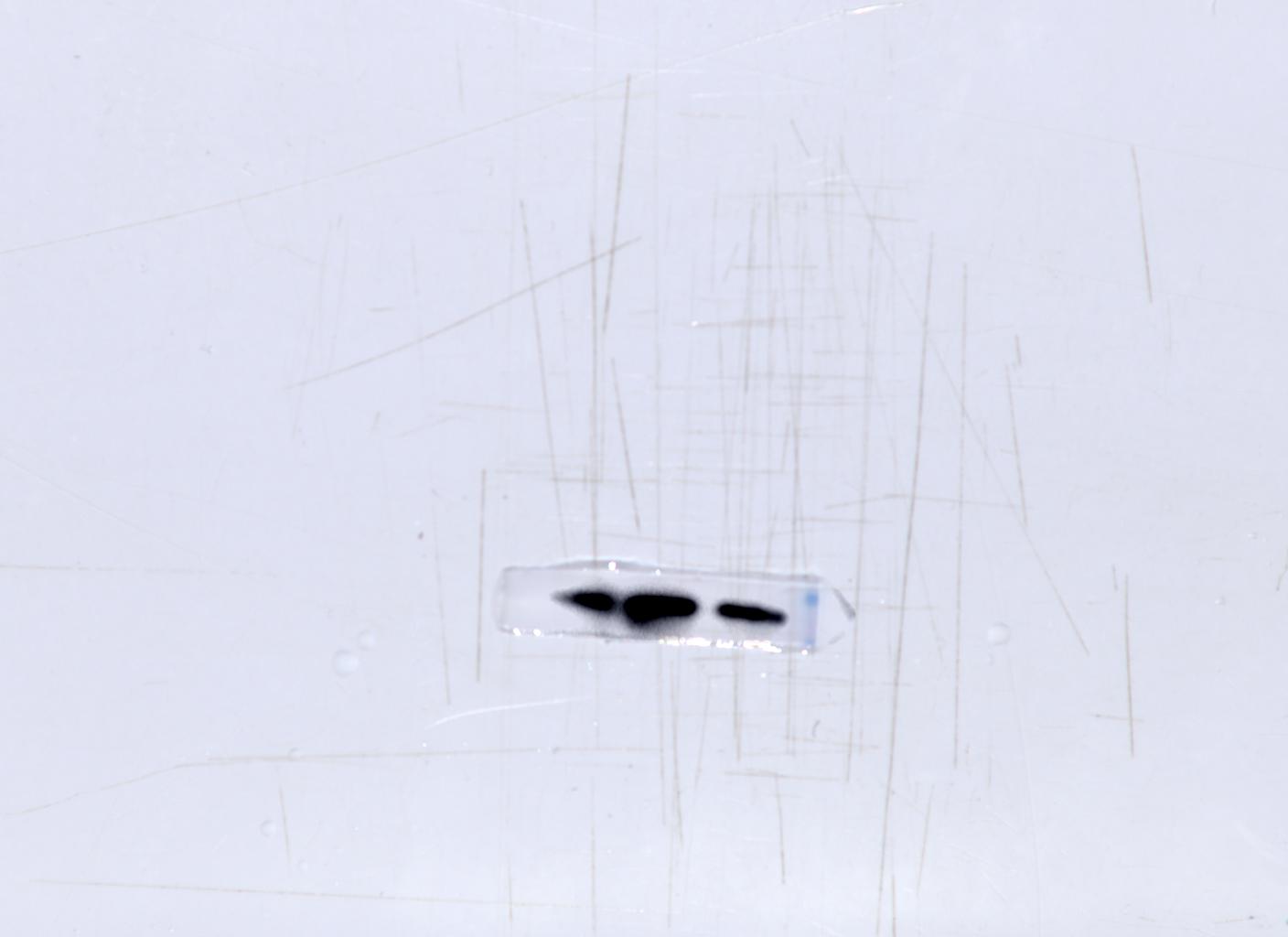

Supplement: Supplementary file 1 [file DataSheet1.ZIP › Raw Data 2/WB/figure 4A/psmad3 9.jpg]

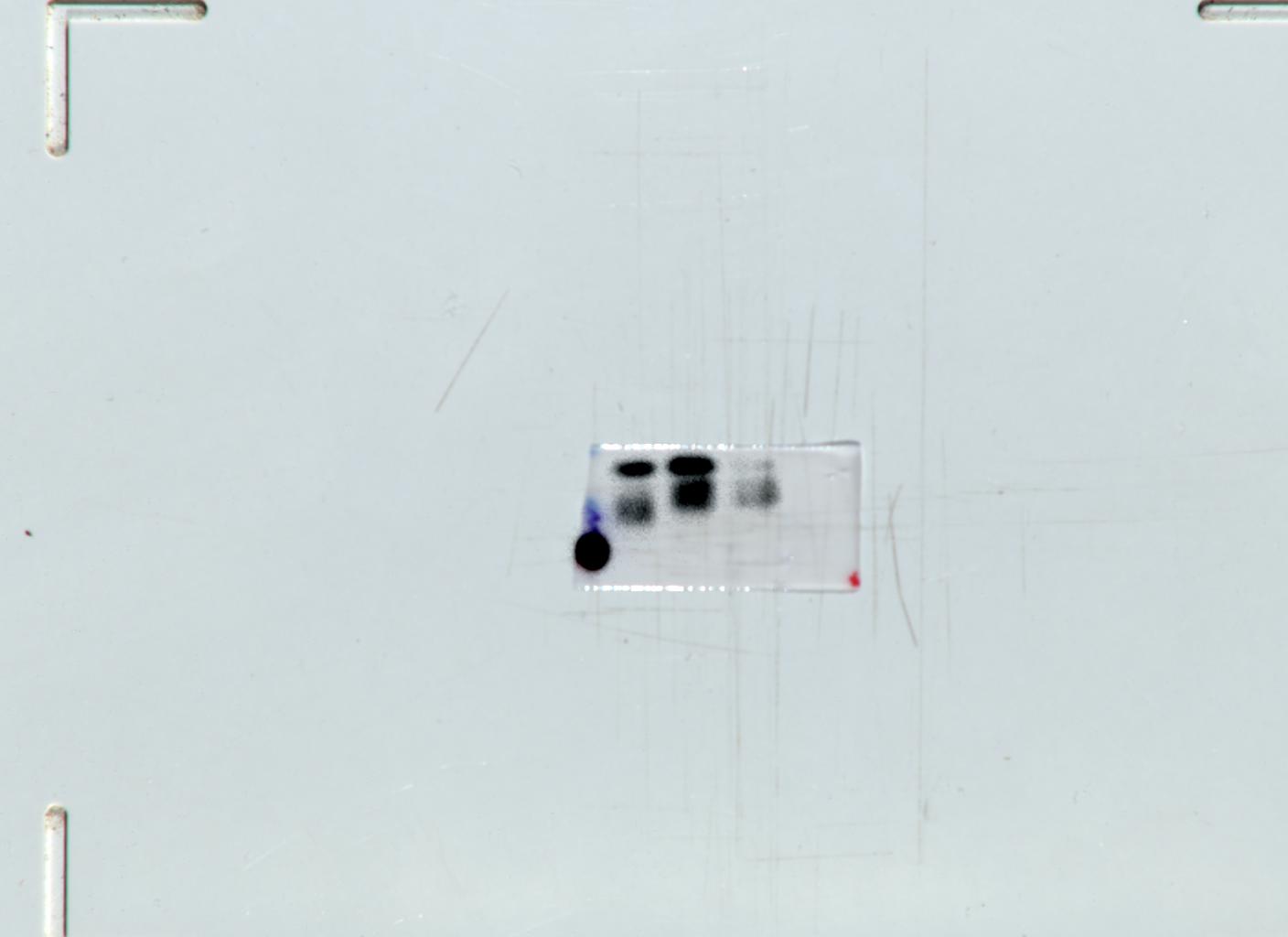

Supplement: Supplementary file 1 [file DataSheet1.ZIP › Raw Data 2/WB/figure 4A/psmad3 8.jpg]

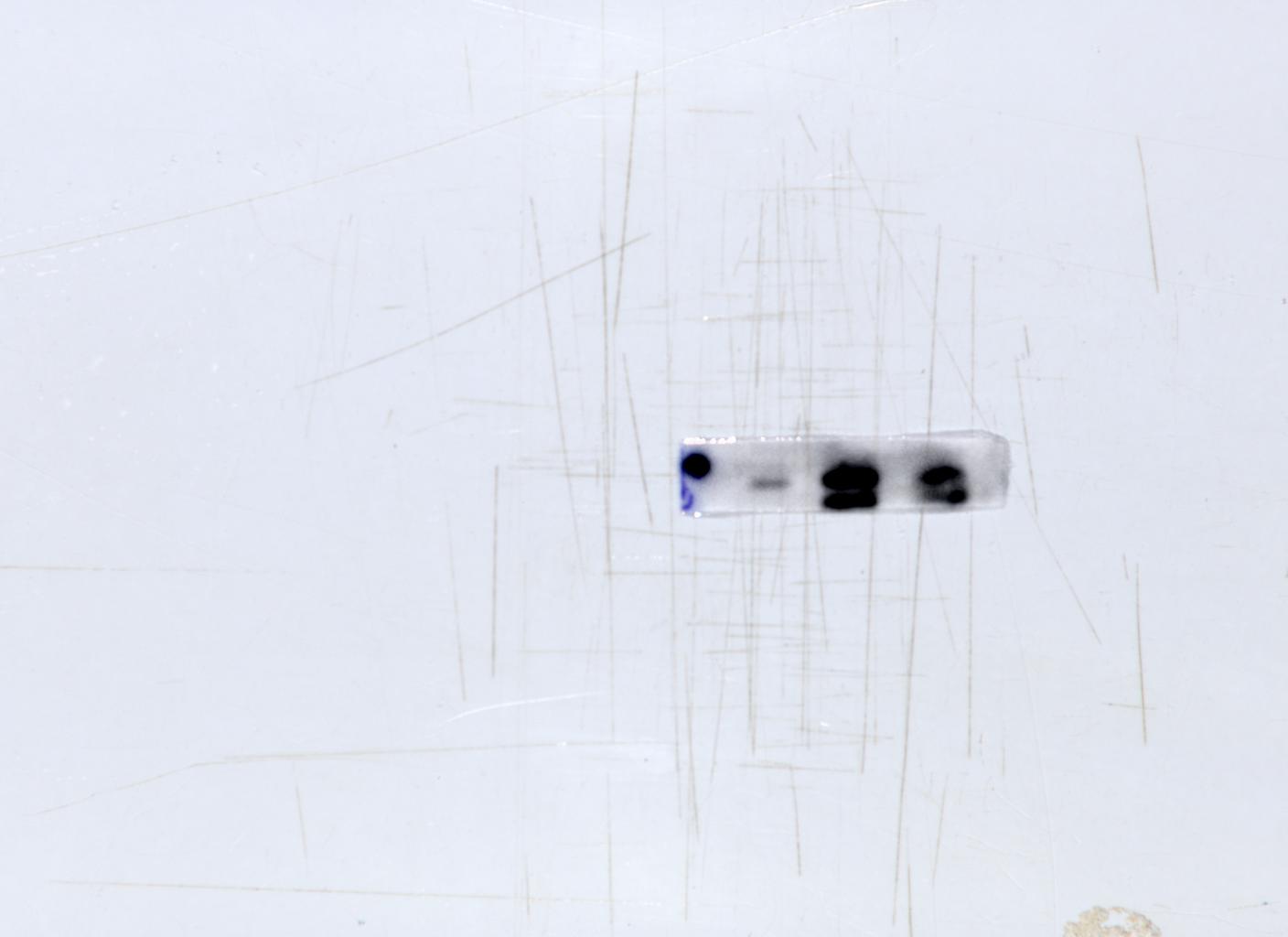

Supplement: Supplementary file 1 [file DataSheet1.ZIP › Raw Data 2/WB/figure 4A/psmad3 10.jpg]

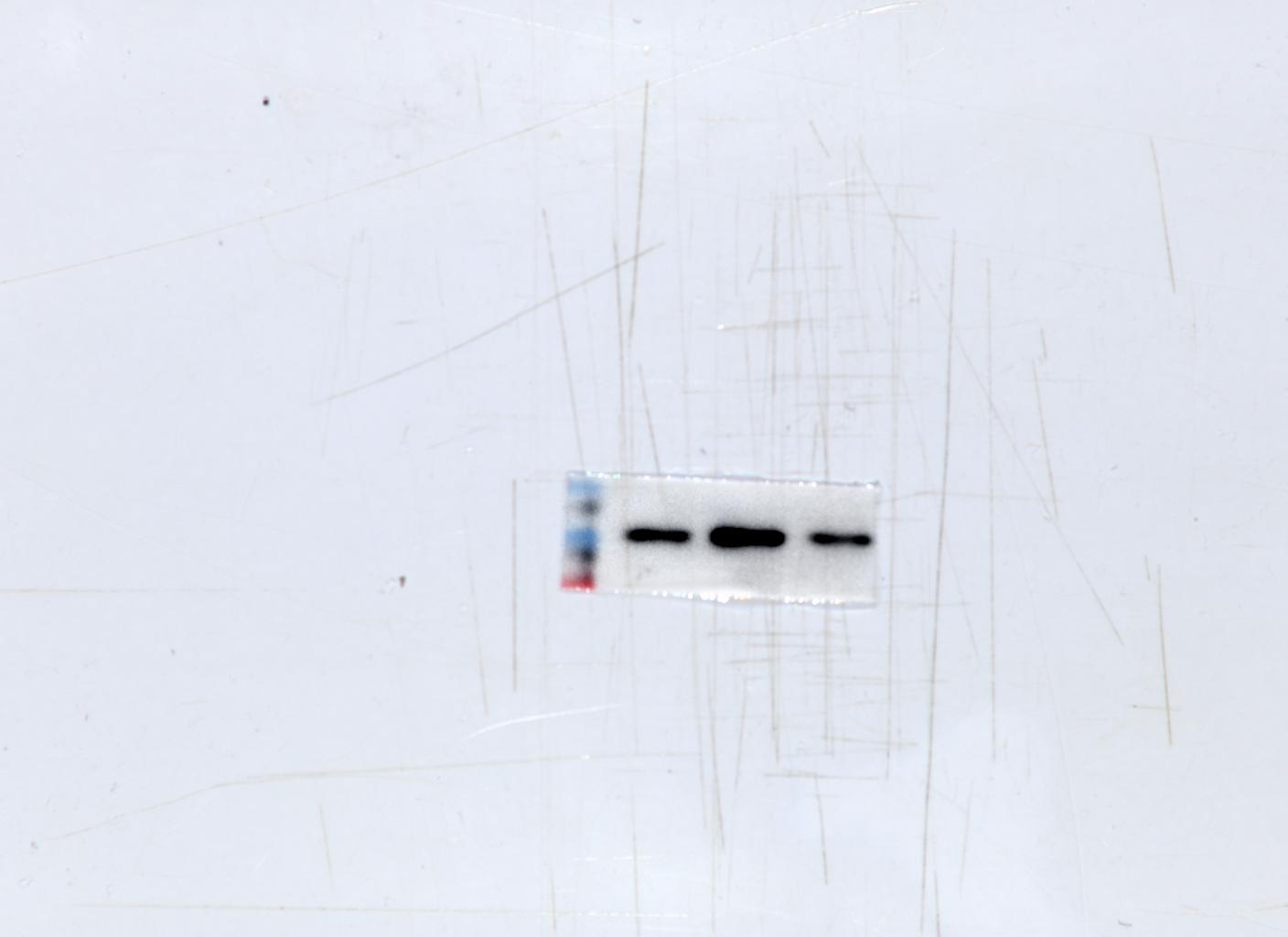

Supplement: Supplementary file 1 [file DataSheet1.ZIP › Raw Data 2/WB/figure 4A/tgf 4.jpg]

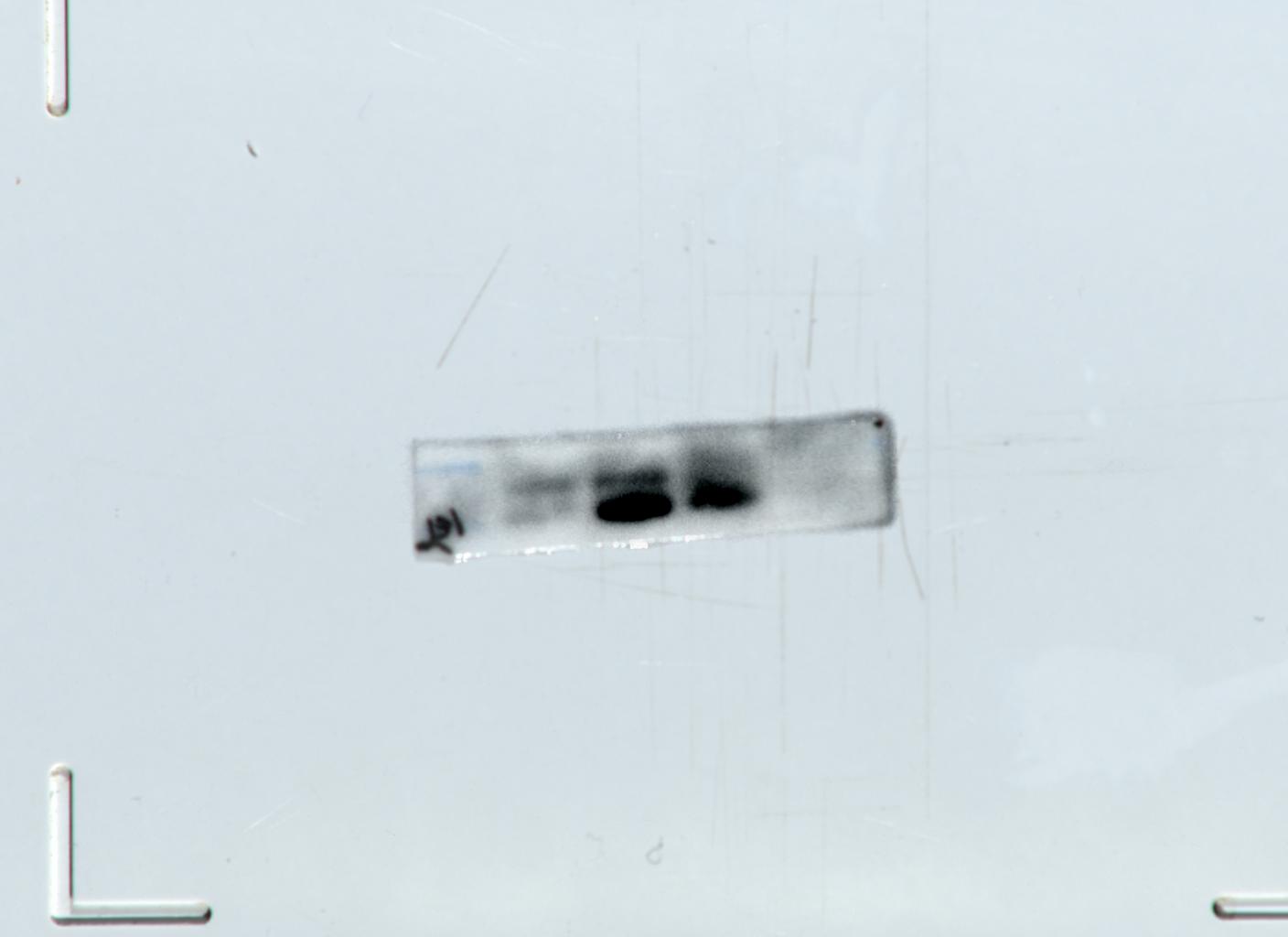

Supplement: Supplementary file 1 [file DataSheet1.ZIP › Raw Data 2/WB/figure 4A/tgf 5.jpg]

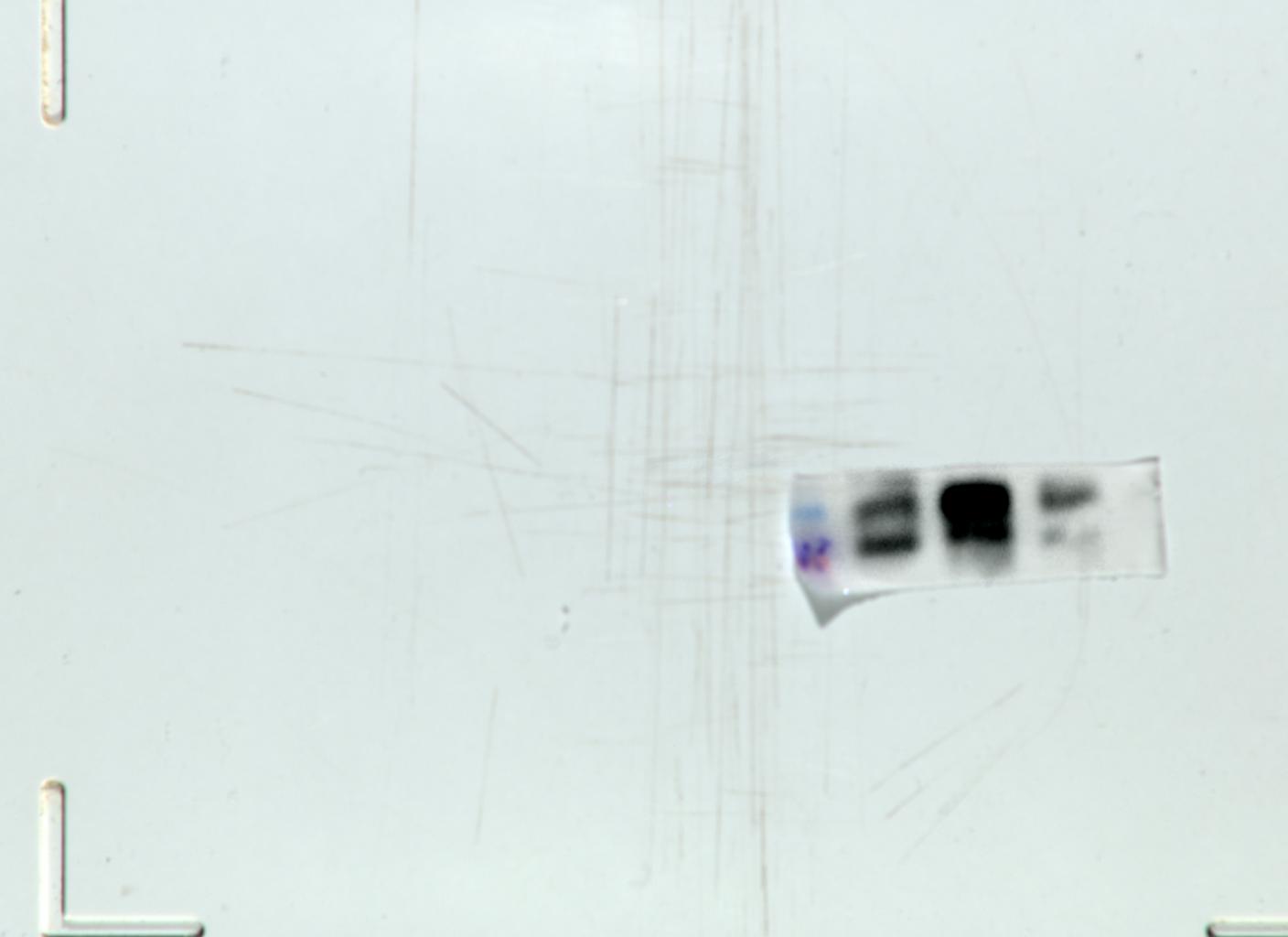

Supplement: Supplementary file 1 [file DataSheet1.ZIP › Raw Data 2/WB/figure 5A/erk 20.jpg]

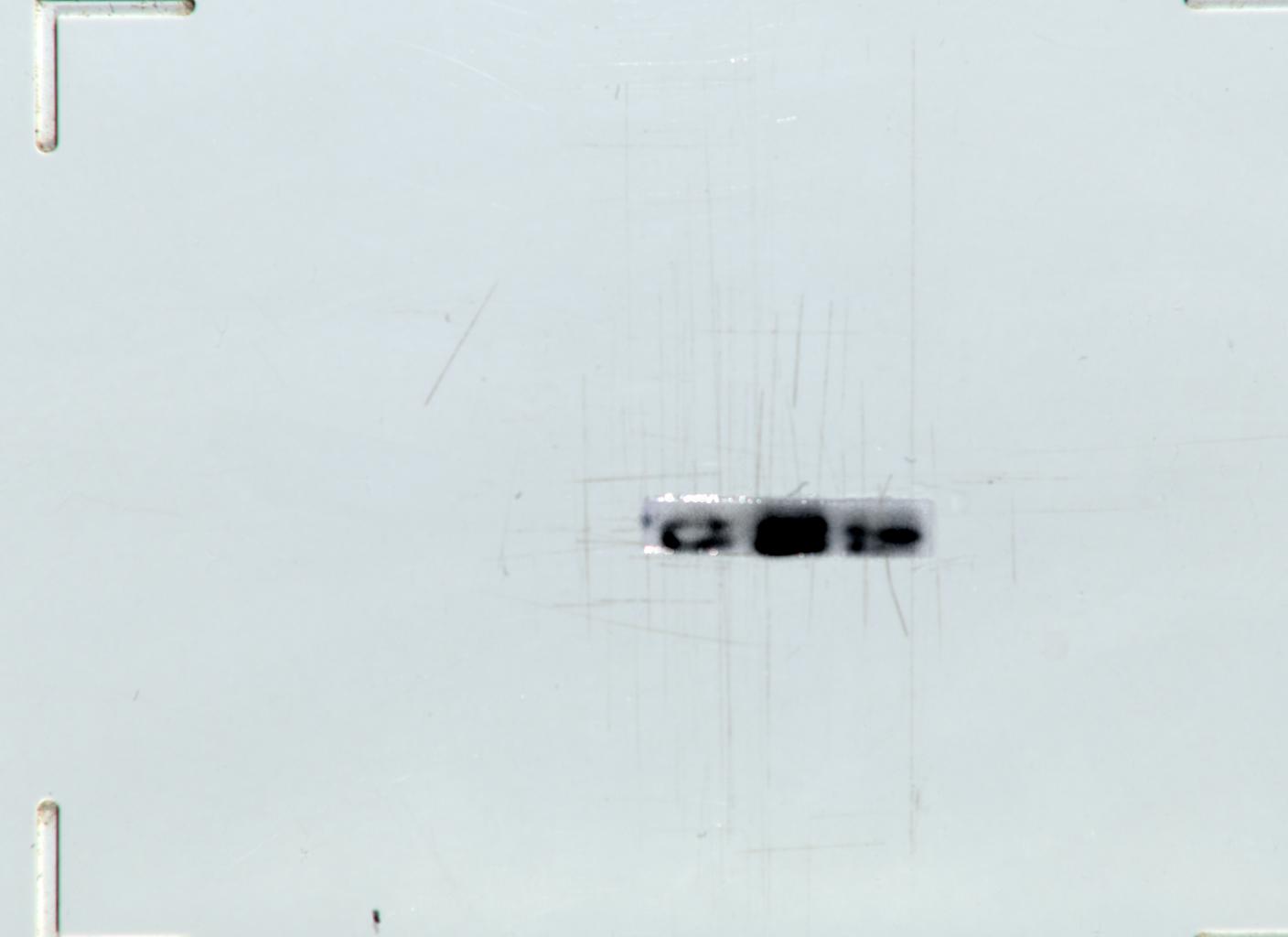

Supplement: Supplementary file 1 [file DataSheet1.ZIP › Raw Data 2/WB/figure 5A/erk 21.jpg]

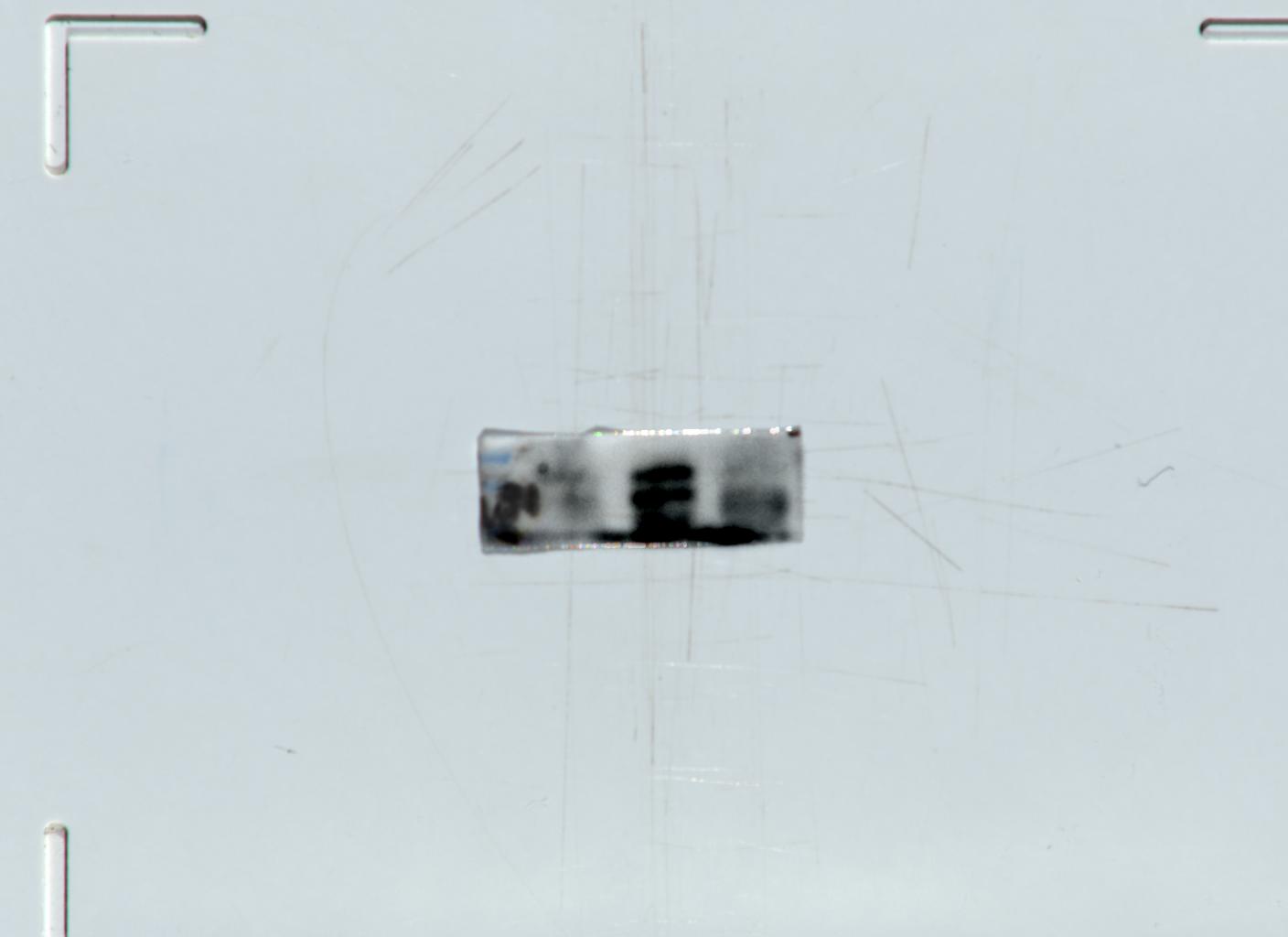

Supplement: Supplementary file 1 [file DataSheet1.ZIP › Raw Data 2/WB/figure 5A/erk 22.jpg]

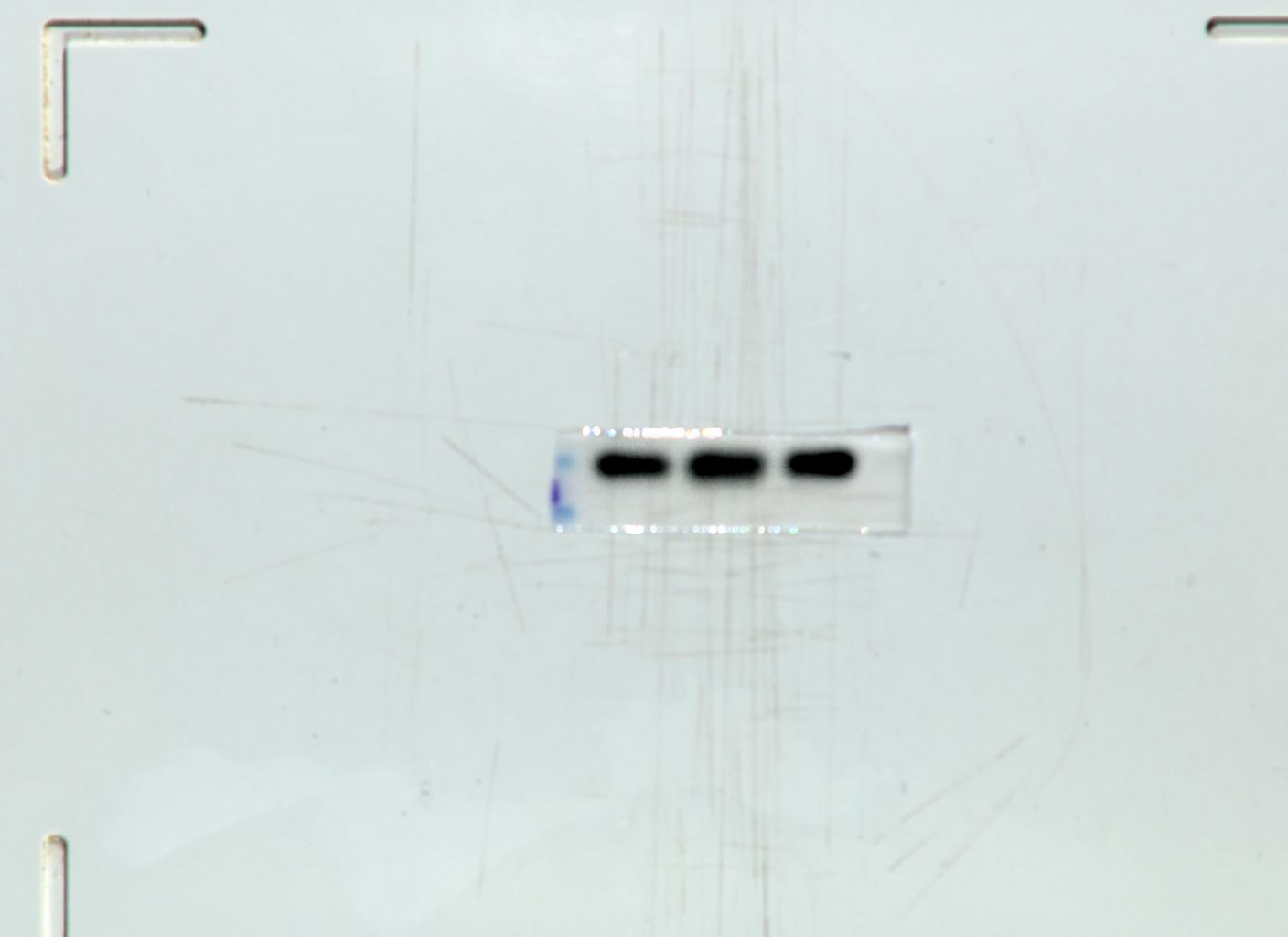

Supplement: Supplementary file 1 [file DataSheet1.ZIP › Raw Data 2/WB/figure 5A/gap 18.jpg]

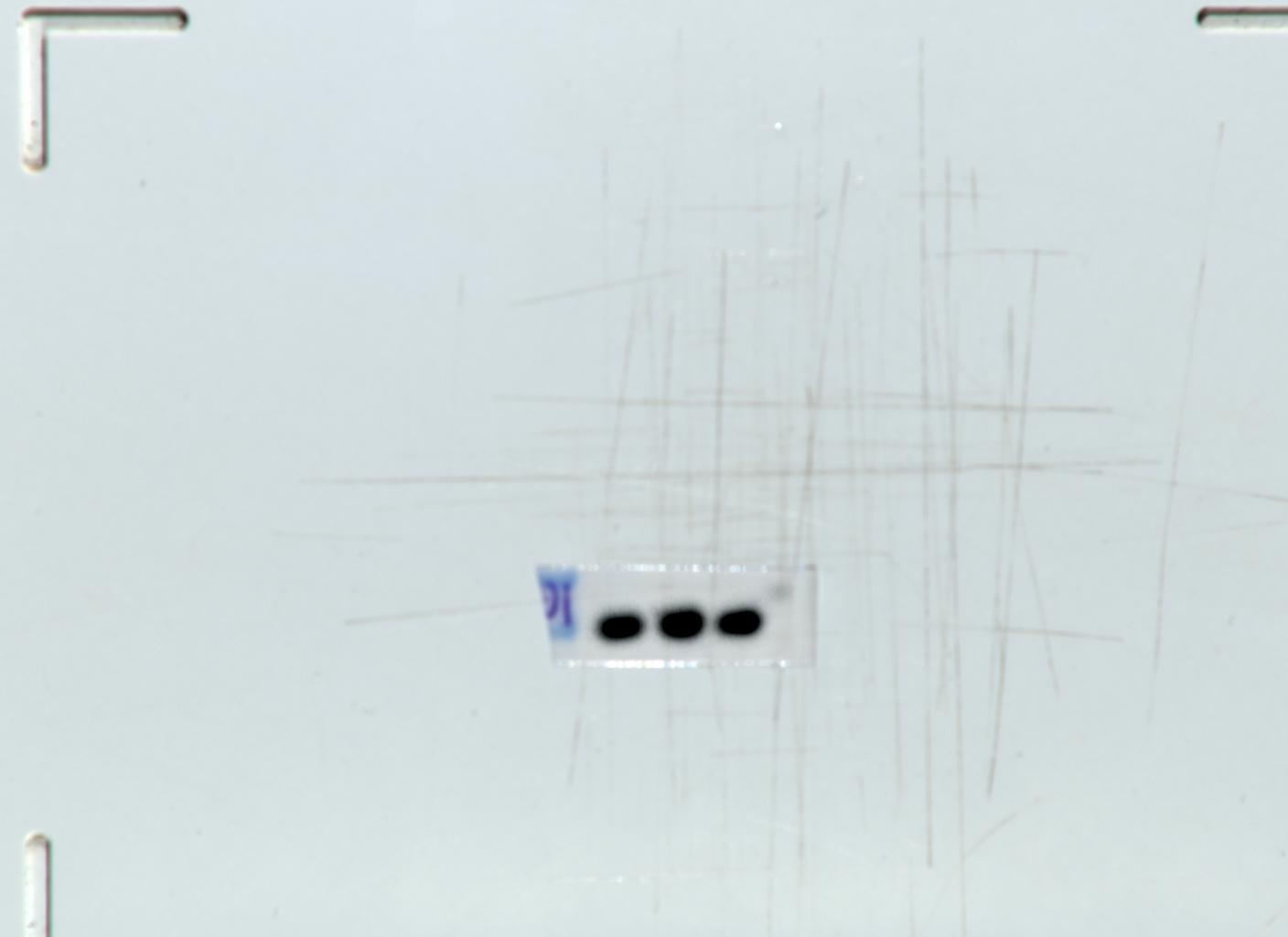

Supplement: Supplementary file 1 [file DataSheet1.ZIP › Raw Data 2/WB/figure 5A/gap 14.jpg]

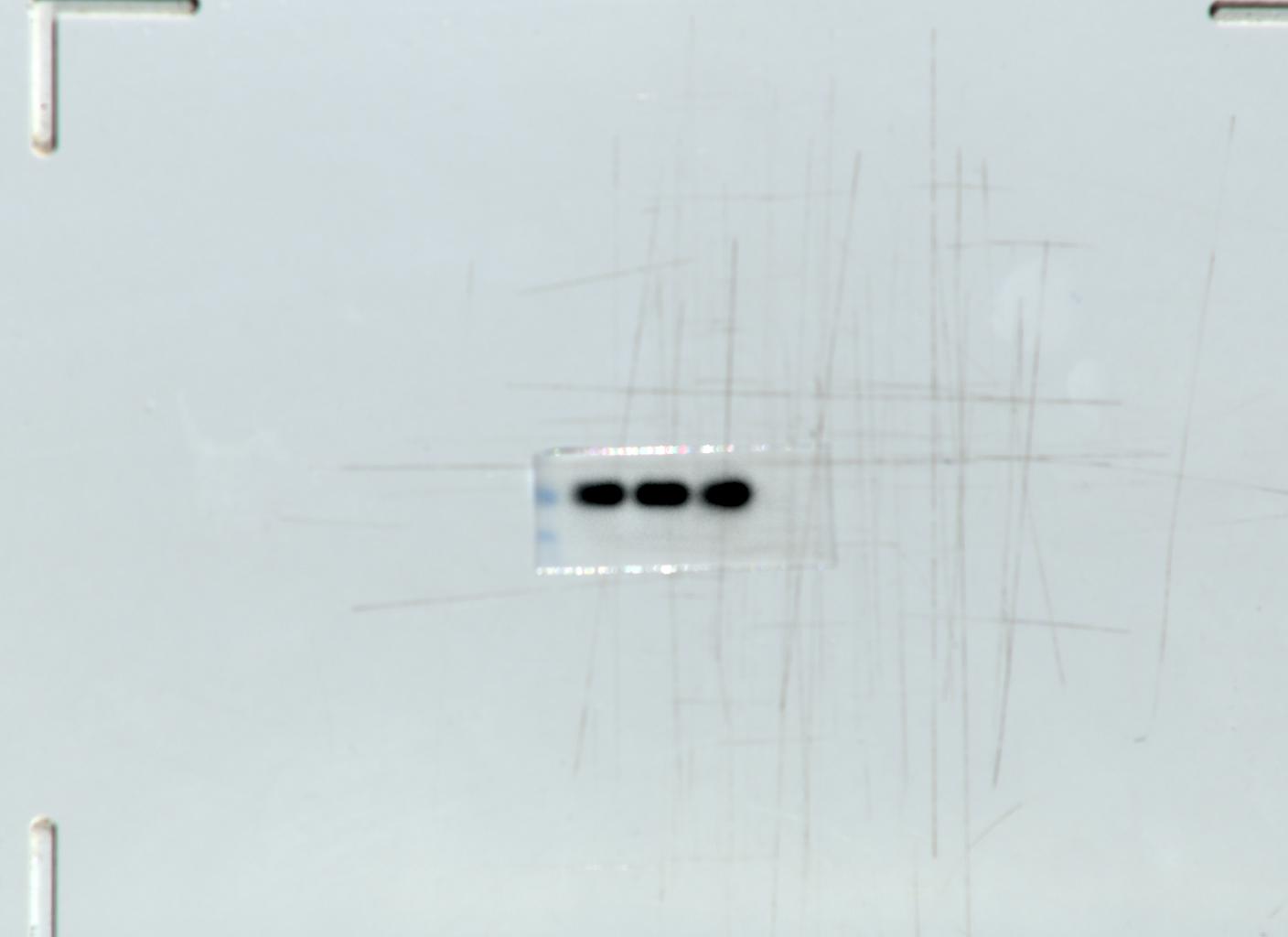

Supplement: Supplementary file 1 [file DataSheet1.ZIP › Raw Data 2/WB/figure 5A/gap 15.jpg]

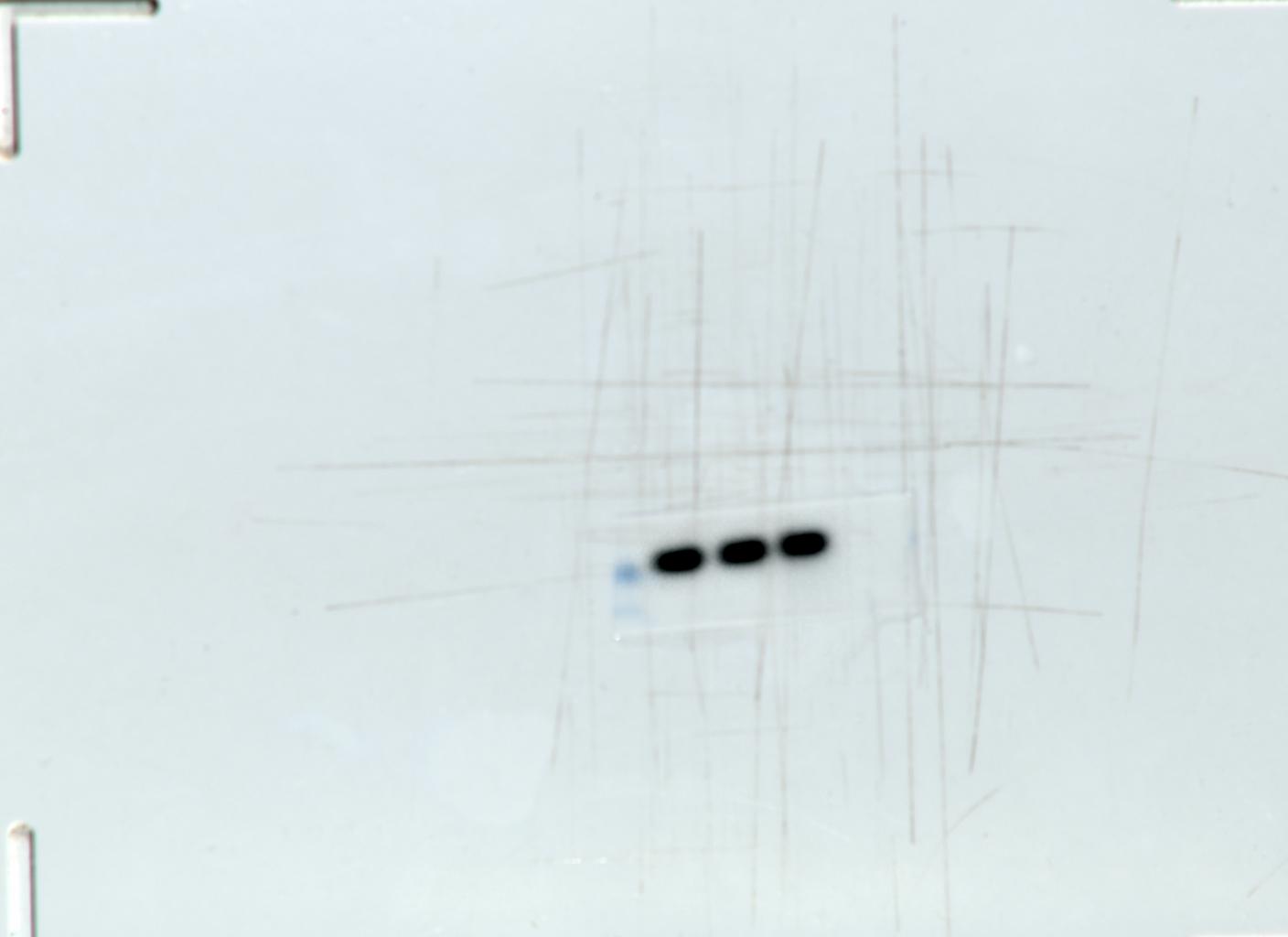

Supplement: Supplementary file 1 [file DataSheet1.ZIP › Raw Data 2/WB/figure 5A/gap 16.jpg]

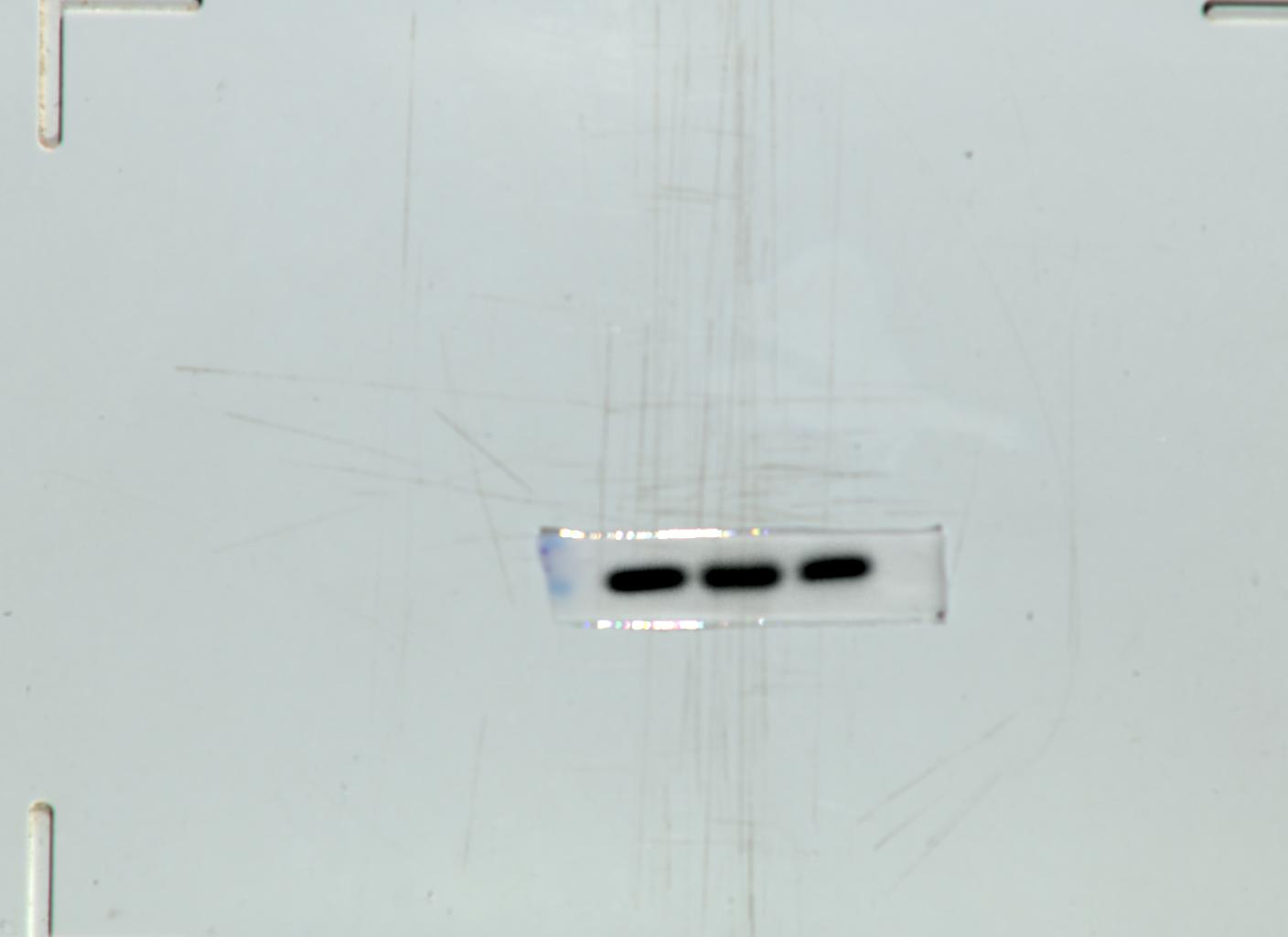

Supplement: Supplementary file 1 [file DataSheet1.ZIP › Raw Data 2/WB/figure 5A/gap 19.jpg]

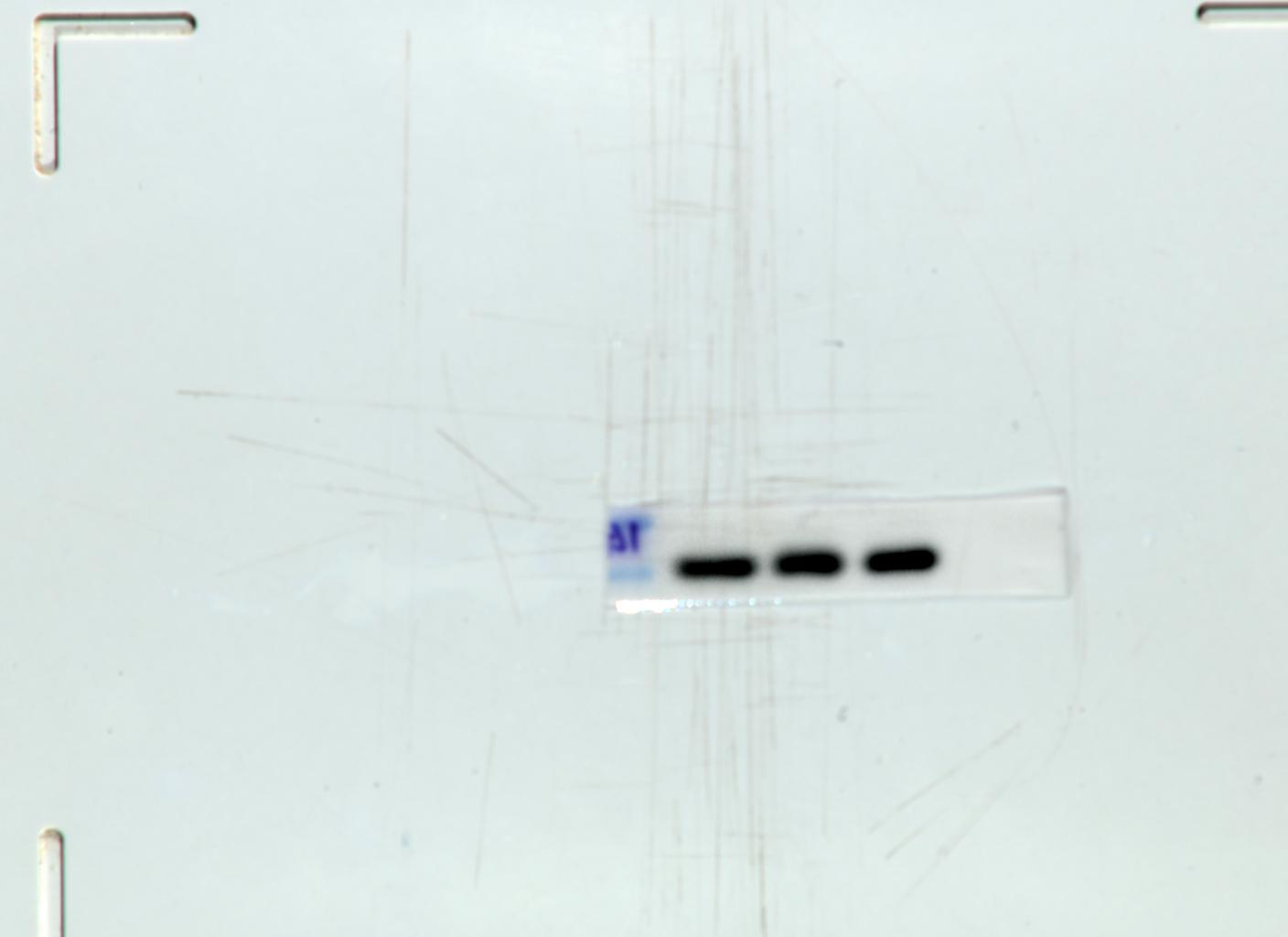

Supplement: Supplementary file 1 [file DataSheet1.ZIP › Raw Data 2/WB/figure 5A/gap_17.jpg]

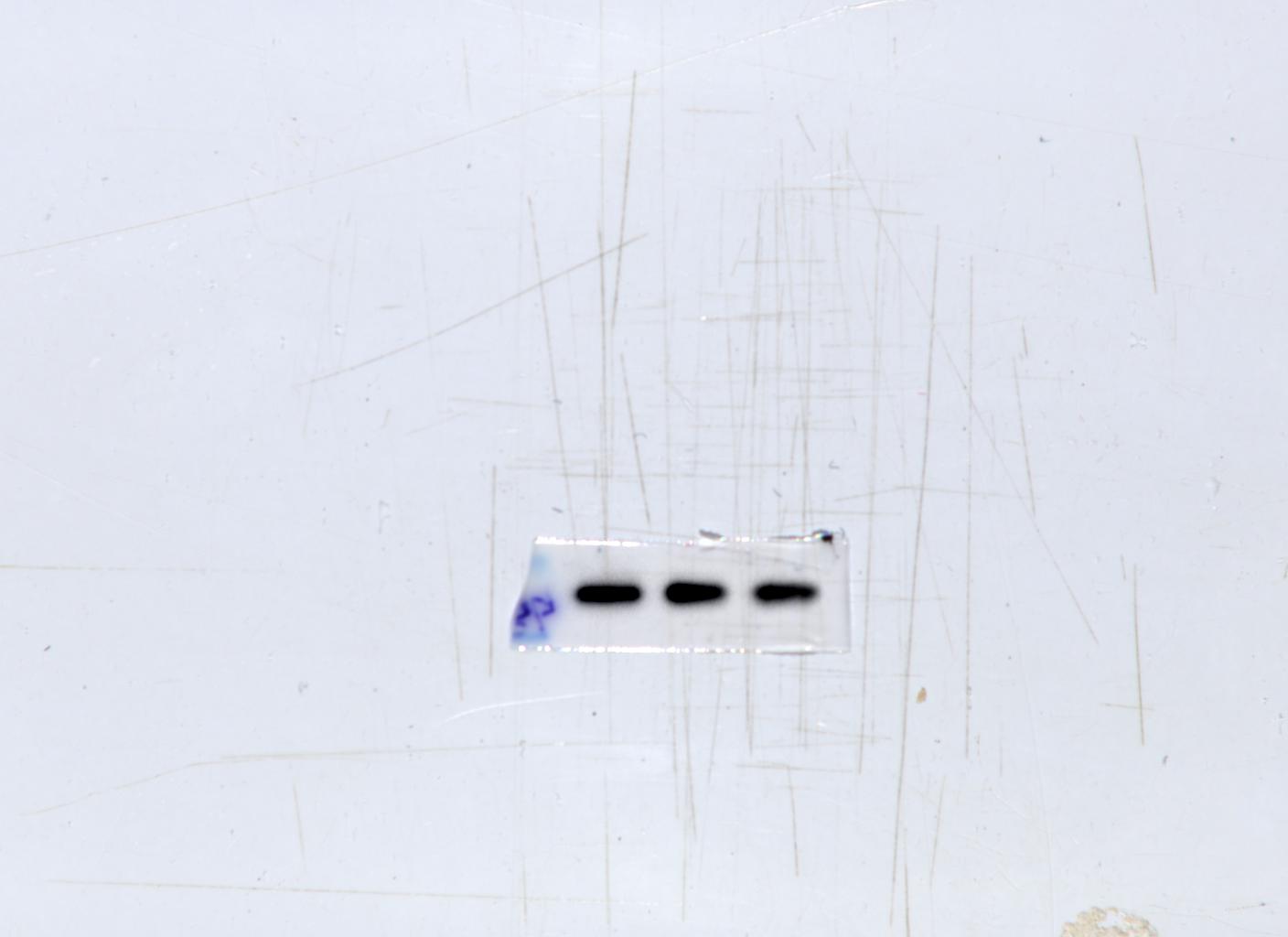

Supplement: Supplementary file 1 [file DataSheet1.ZIP › Raw Data 2/WB/figure 5A/lu-g 20.jpg]

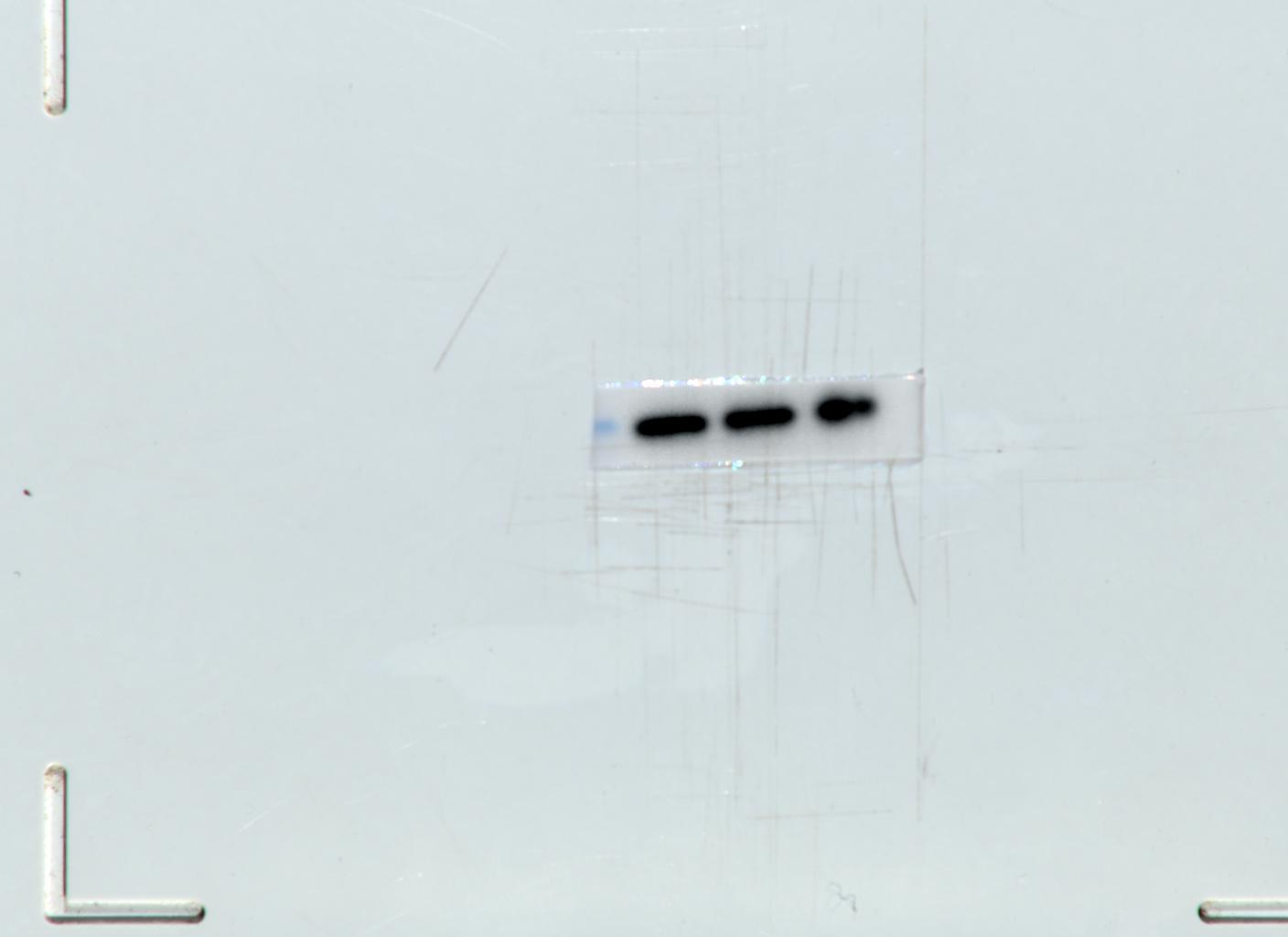

Supplement: Supplementary file 1 [file DataSheet1.ZIP › Raw Data 2/WB/figure 5A/lu-g 21.jpg]

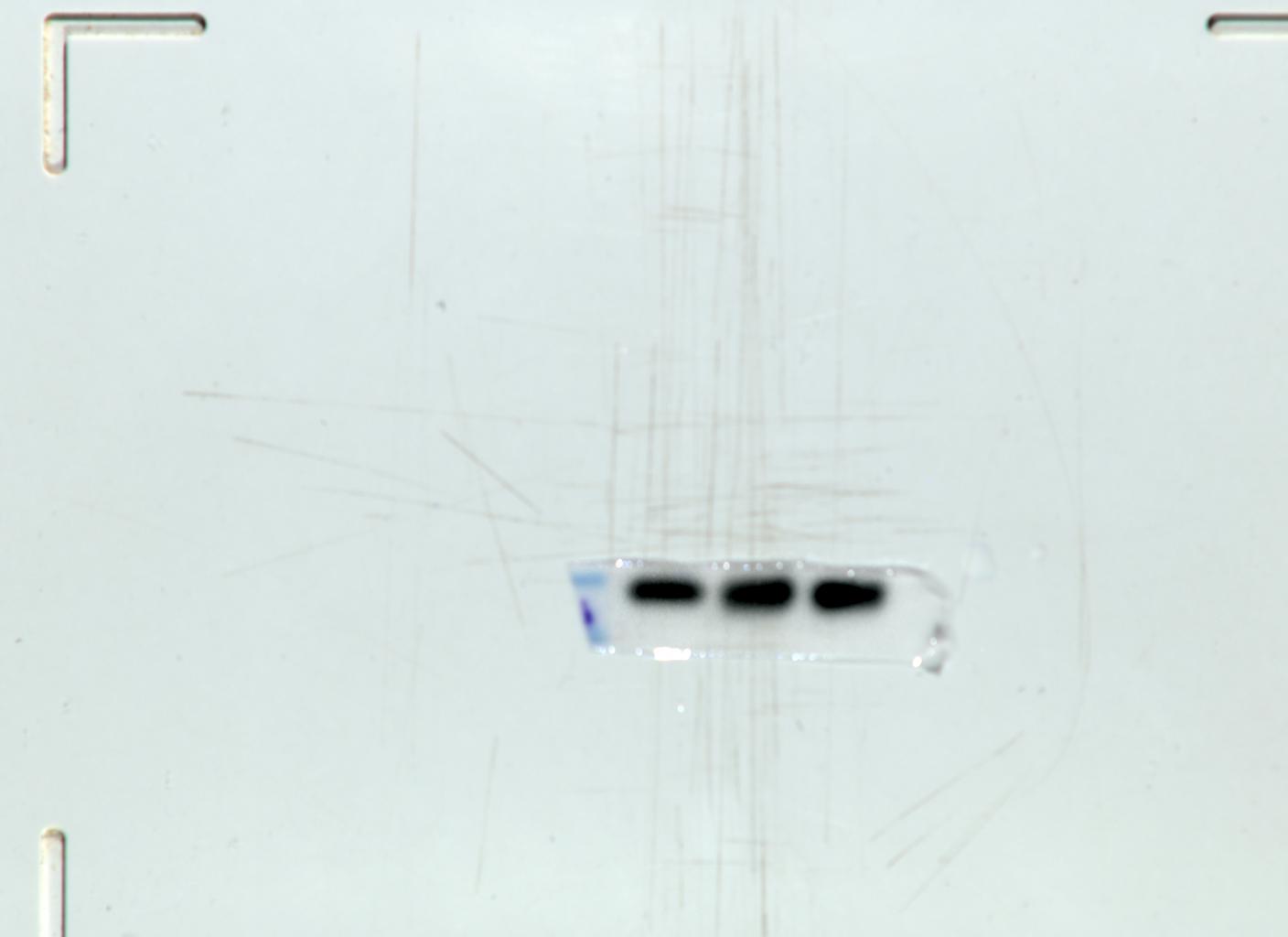

Supplement: Supplementary file 1 [file DataSheet1.ZIP › Raw Data 2/WB/figure 5A/lu-g 22.jpg]

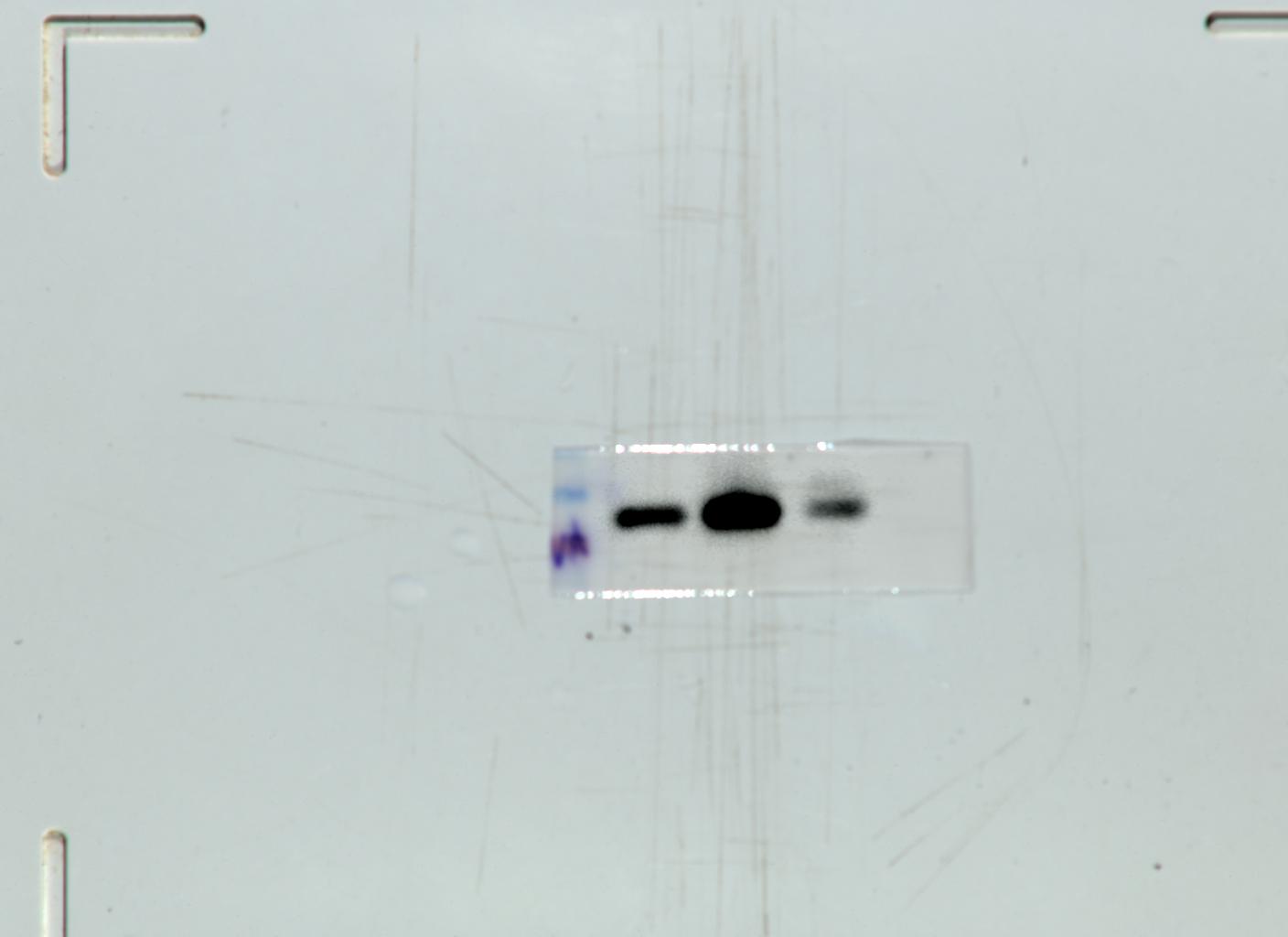

Supplement: Supplementary file 1 [file DataSheet1.ZIP › Raw Data 2/WB/figure 5A/nox2 19.jpg]

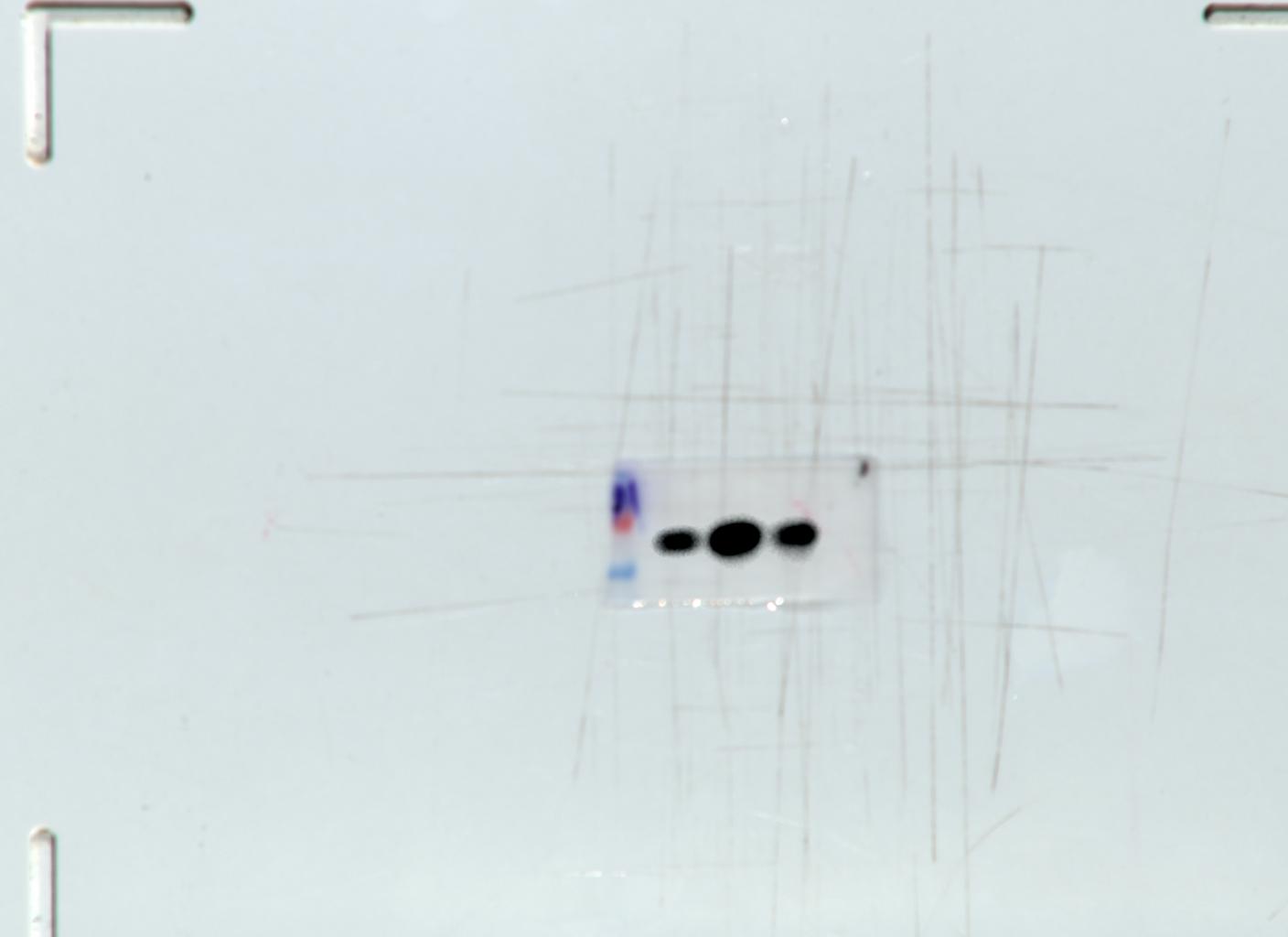

Supplement: Supplementary file 1 [file DataSheet1.ZIP › Raw Data 2/WB/figure 5A/nox2 14.jpg]

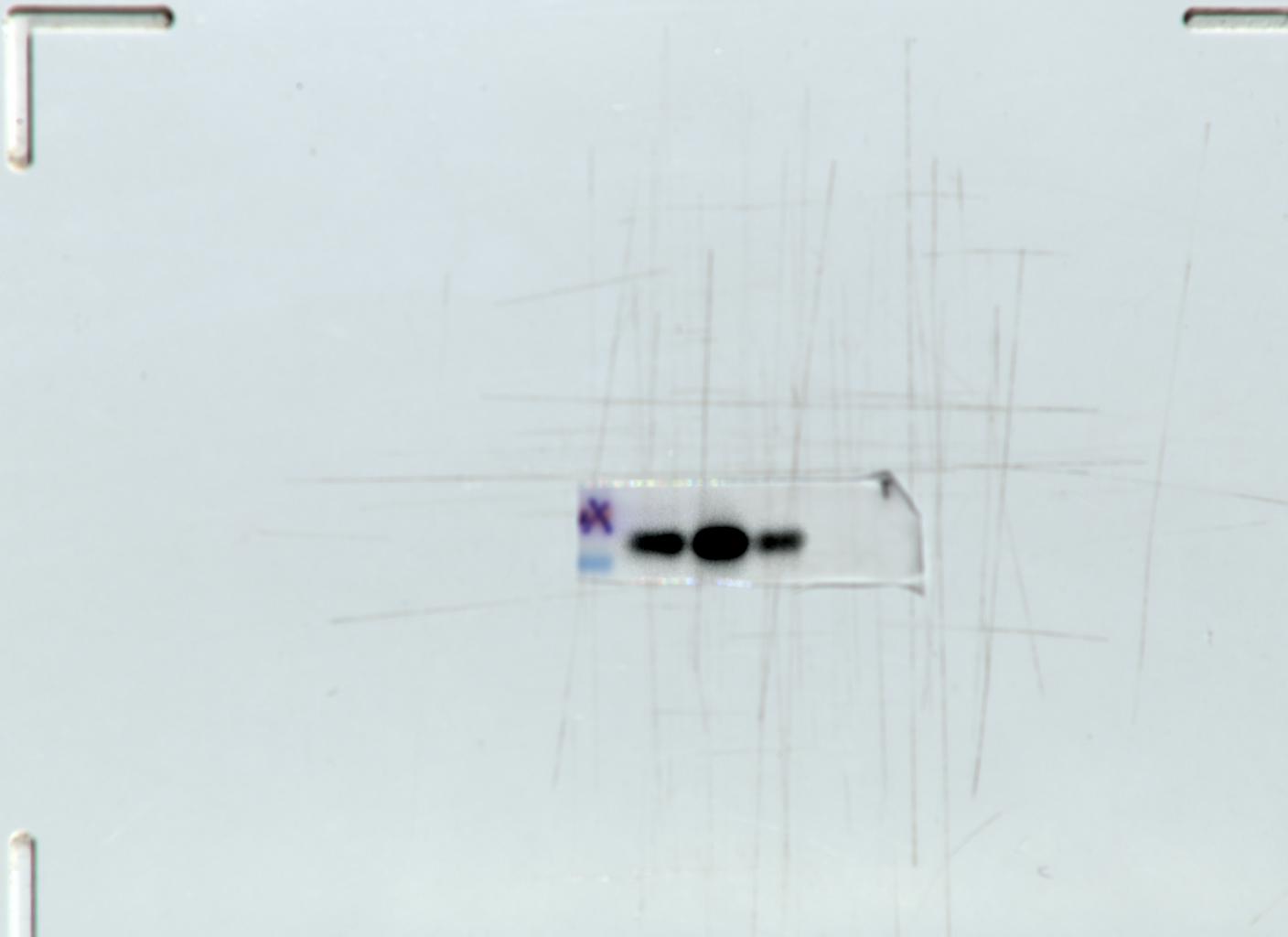

Supplement: Supplementary file 1 [file DataSheet1.ZIP › Raw Data 2/WB/figure 5A/nox2 15.jpg]

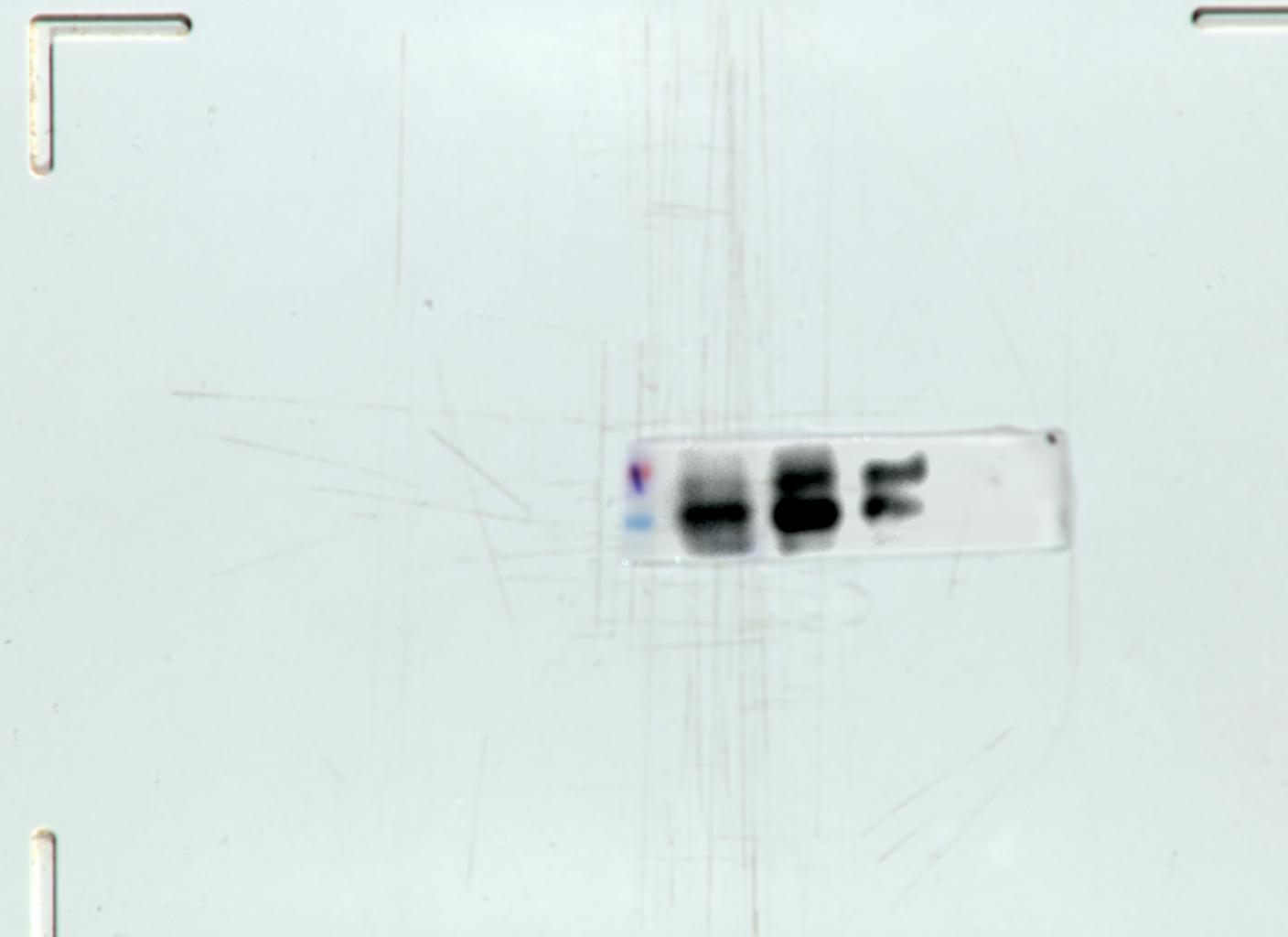

Supplement: Supplementary file 1 [file DataSheet1.ZIP › Raw Data 2/WB/figure 5A/nox4 16.jpg]

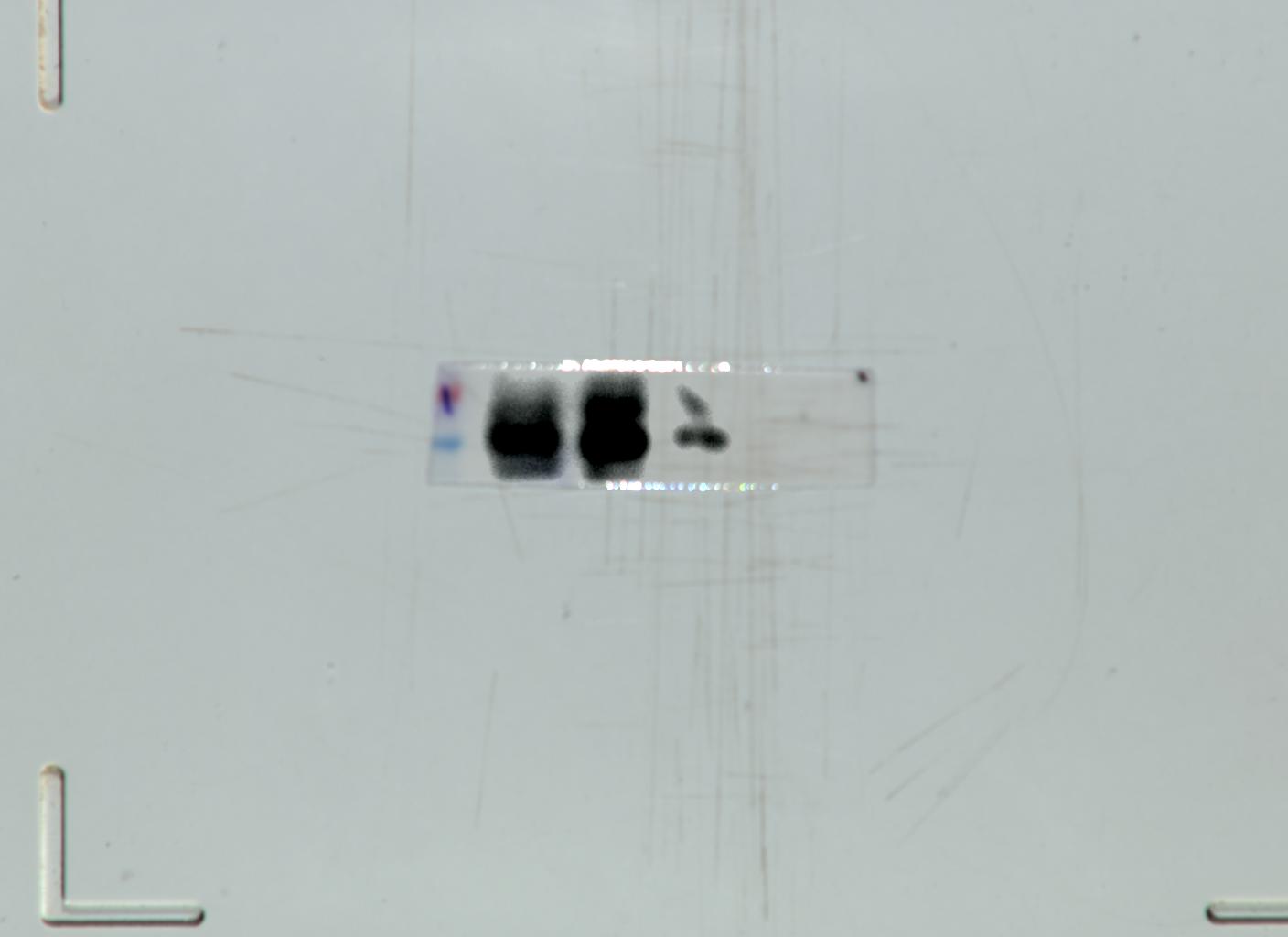

Supplement: Supplementary file 1 [file DataSheet1.ZIP › Raw Data 2/WB/figure 5A/nox4 17.jpg]

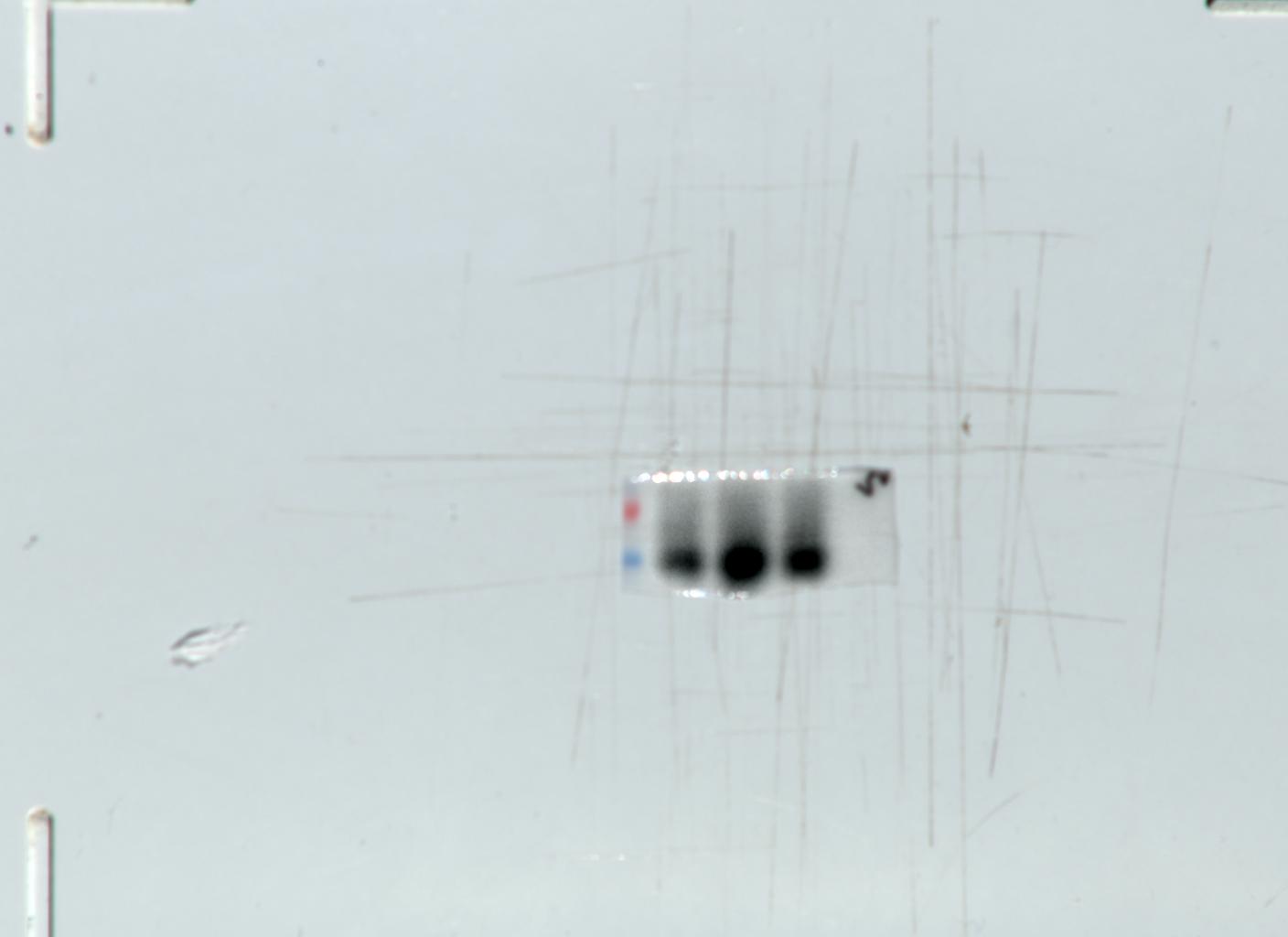

Supplement: Supplementary file 1 [file DataSheet1.ZIP › Raw Data 2/WB/figure 5A/nox4 18.jpg]

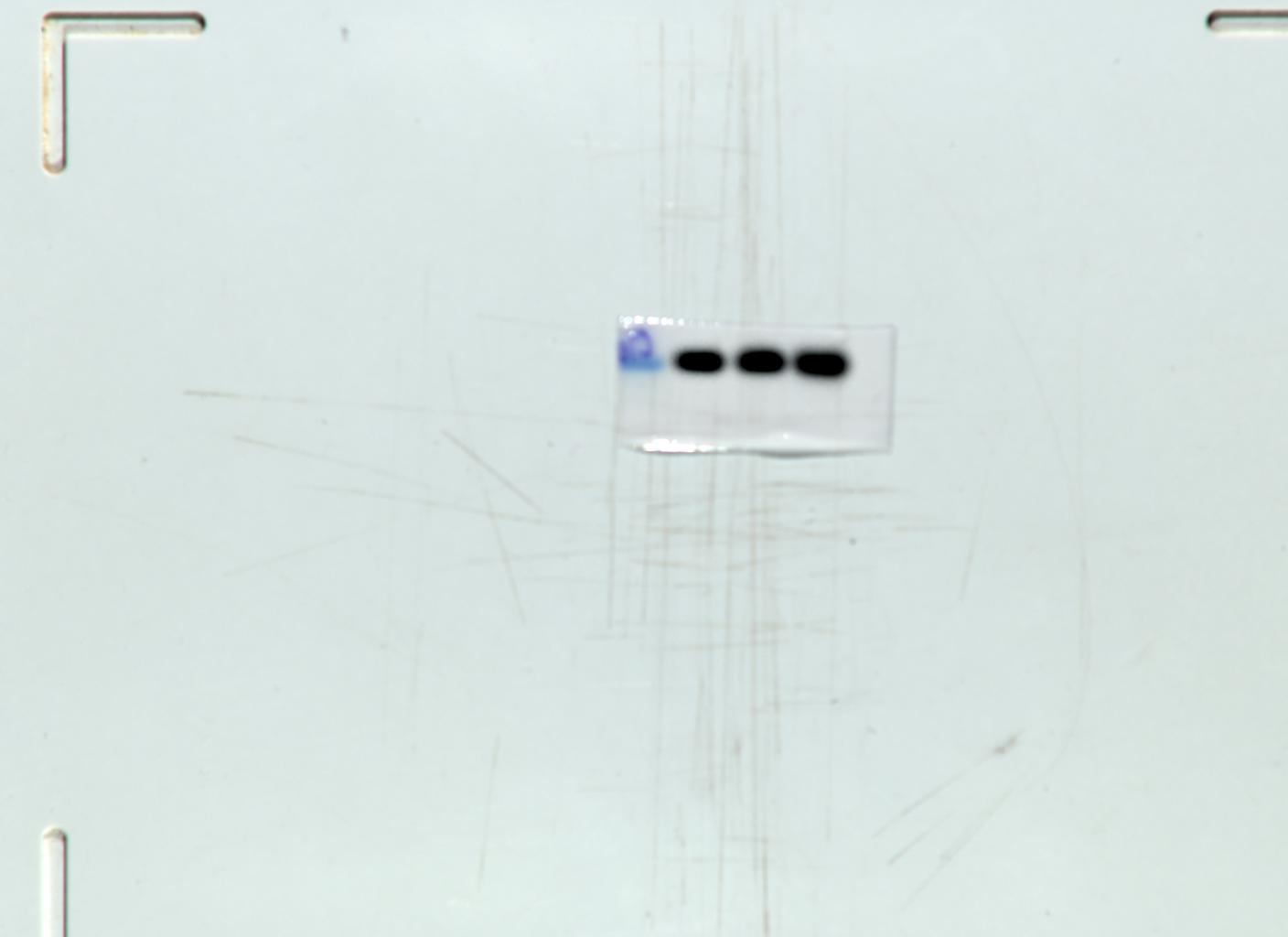

Supplement: Supplementary file 1 [file DataSheet1.ZIP › Raw Data 2/WB/figure 6B/gap 11.jpg]

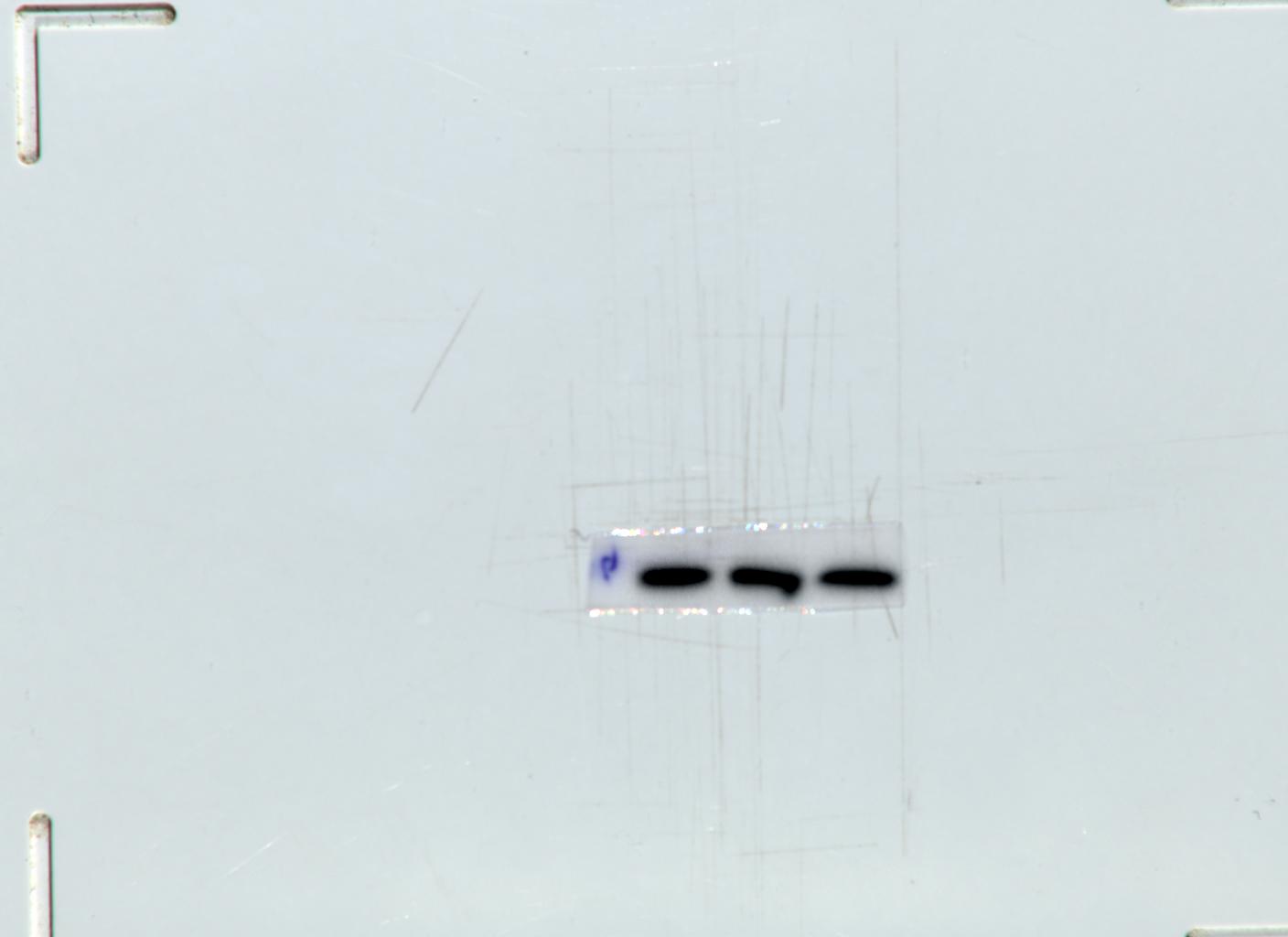

Supplement: Supplementary file 1 [file DataSheet1.ZIP › Raw Data 2/WB/figure 6B/gap 12.jpg]

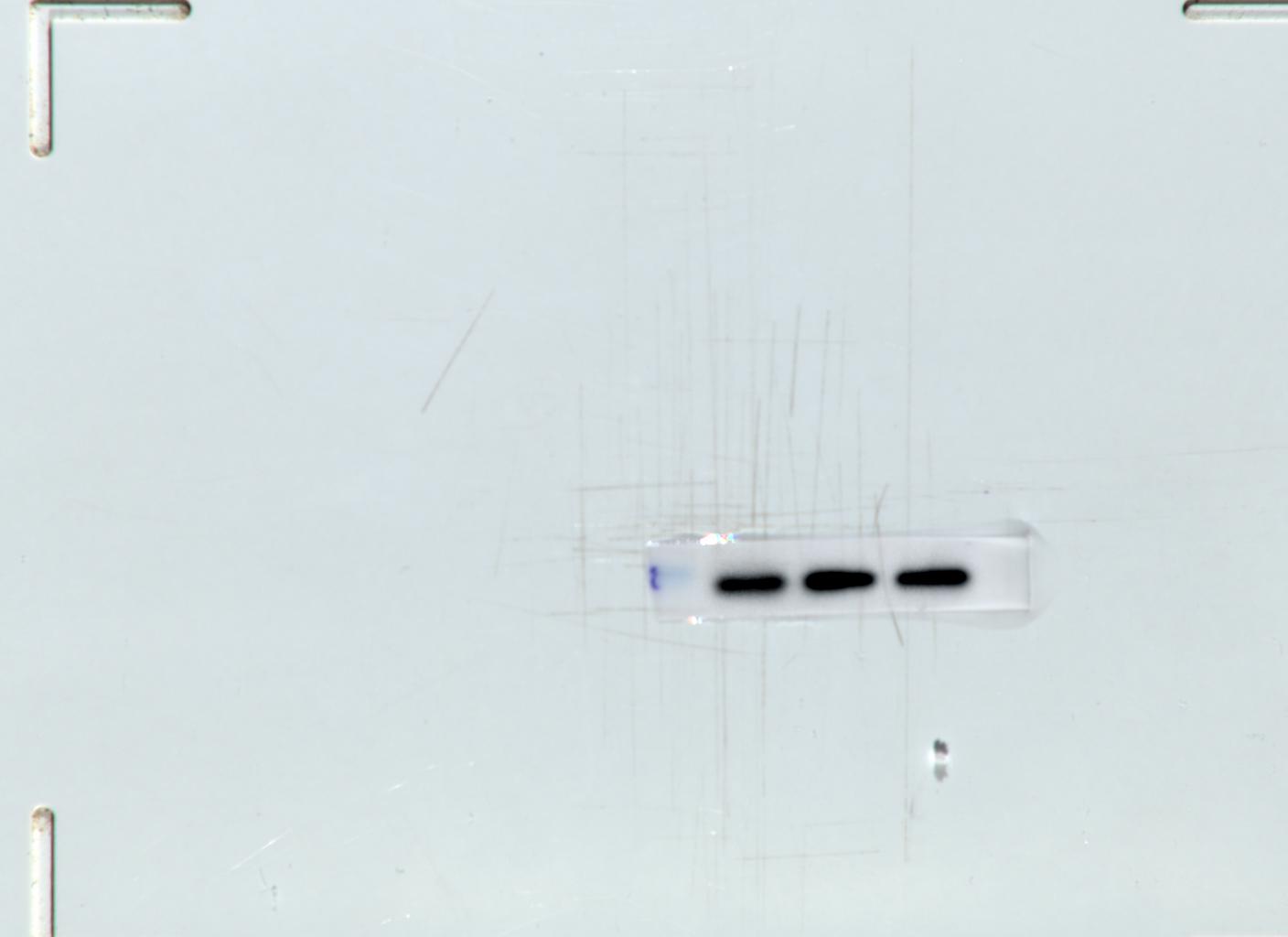

Supplement: Supplementary file 1 [file DataSheet1.ZIP › Raw Data 2/WB/figure 6B/gap 13.jpg]

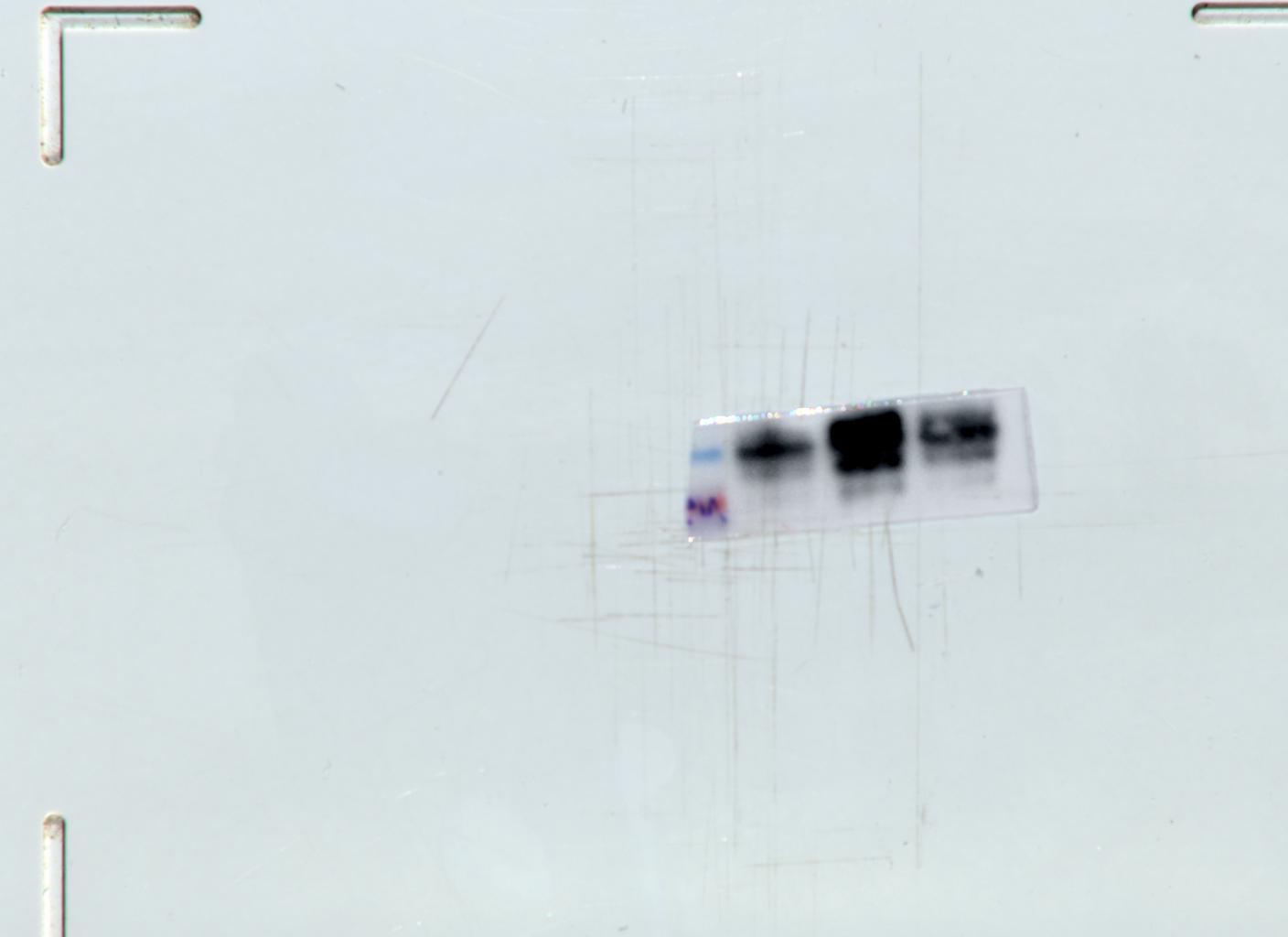

Supplement: Supplementary file 1 [file DataSheet1.ZIP › Raw Data 2/WB/figure 6B/nfkb 12.jpg]

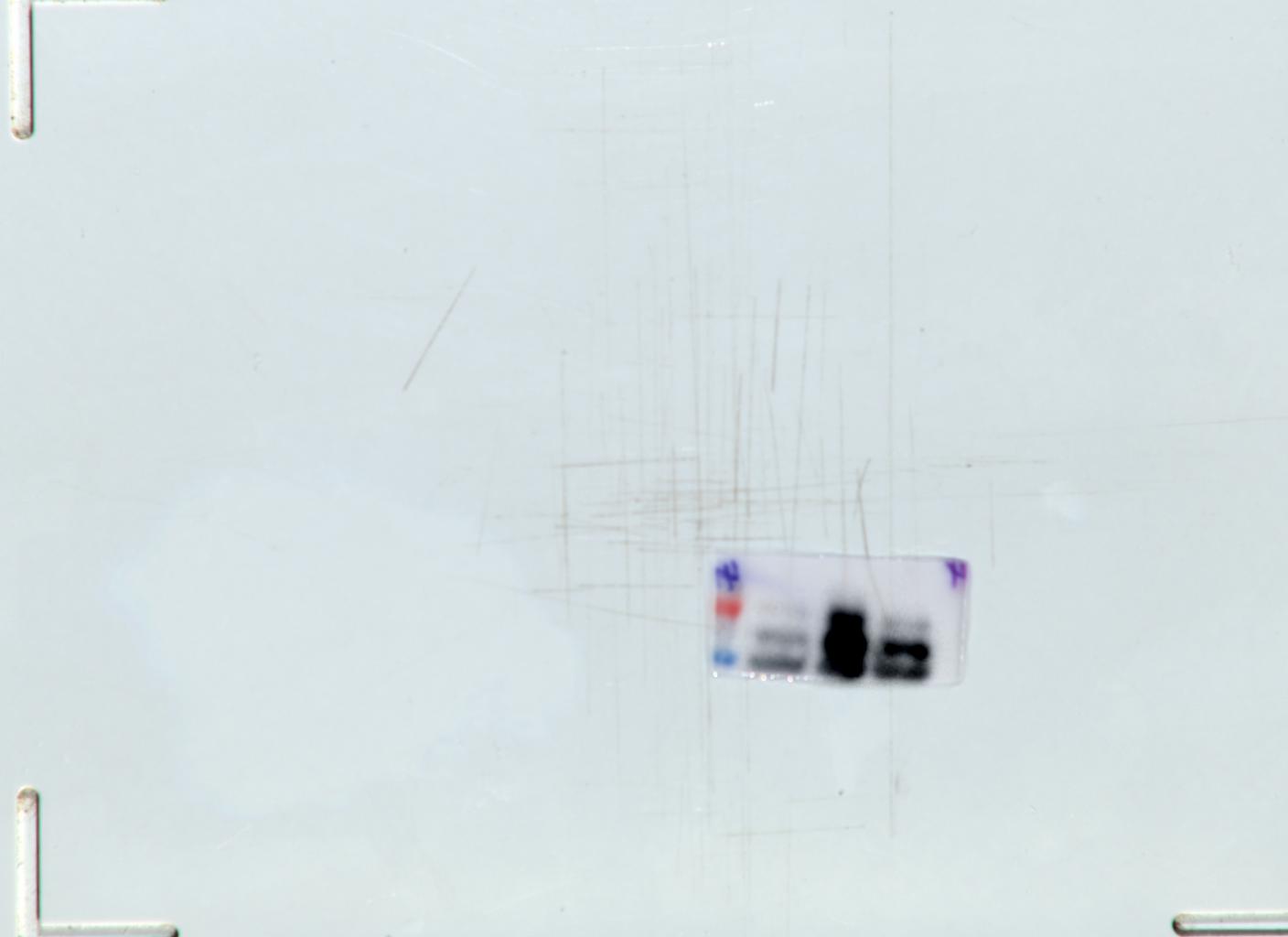

Supplement: Supplementary file 1 [file DataSheet1.ZIP › Raw Data 2/WB/figure 6B/nfkb 11.jpg]

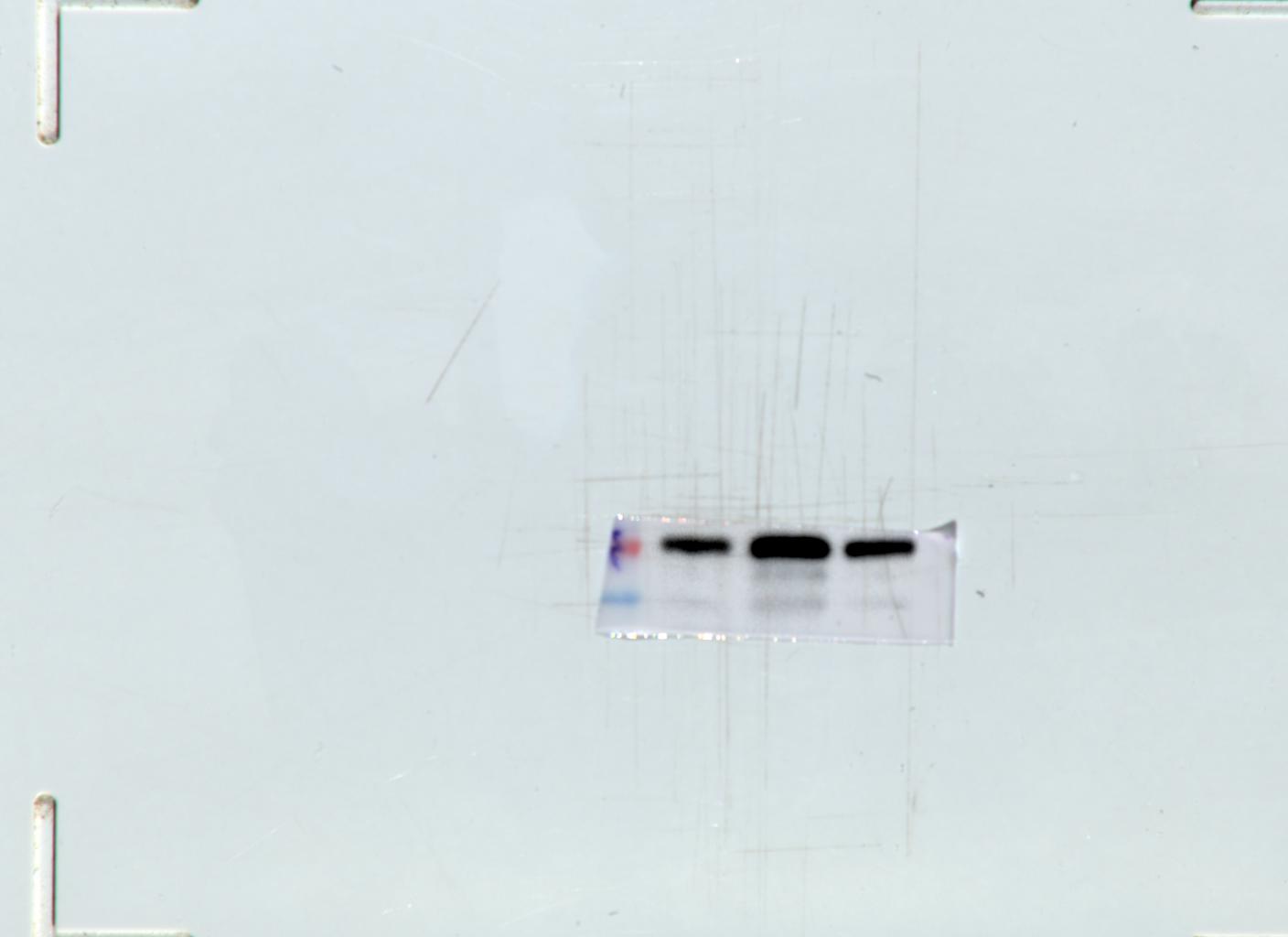

Supplement: Supplementary file 1 [file DataSheet1.ZIP › Raw Data 2/WB/figure 6B/nfkb 13.jpg]
